# Supplementary material for: Mitochondrial translocation of TFEB regulates complex I and inflammation
Source: EMBO Rep. 2024 Jan 23;25(2):704–24. doi: 10.1038/s44319-024-00058-0 (PMC10897448; doi:10.1038/s44319-024-00058-0)
Supplement: Supplementary file 1 — Appendix [file 44319_2024_58_MOESM1_ESM.pdf]

Table of Contents

Page 1	Appendix Fig S1
Page 2	Appendix Fig S2
Page 3	Appendix Fig S3
Page 4	Appendix Fig S4
Page 5	Appendix Fig S4 Legend
Page 6	Appendix Fig S4 Legend (contd)
Page 7	Appendix Fig S5
Page 8	Appendix Fig S6
Page 9	Appendix Fig S7
Page 10	Appendix Fig S7 Legend
Page 11	Appendix Fig S7 Legend (contd)

Appendix Fig S1

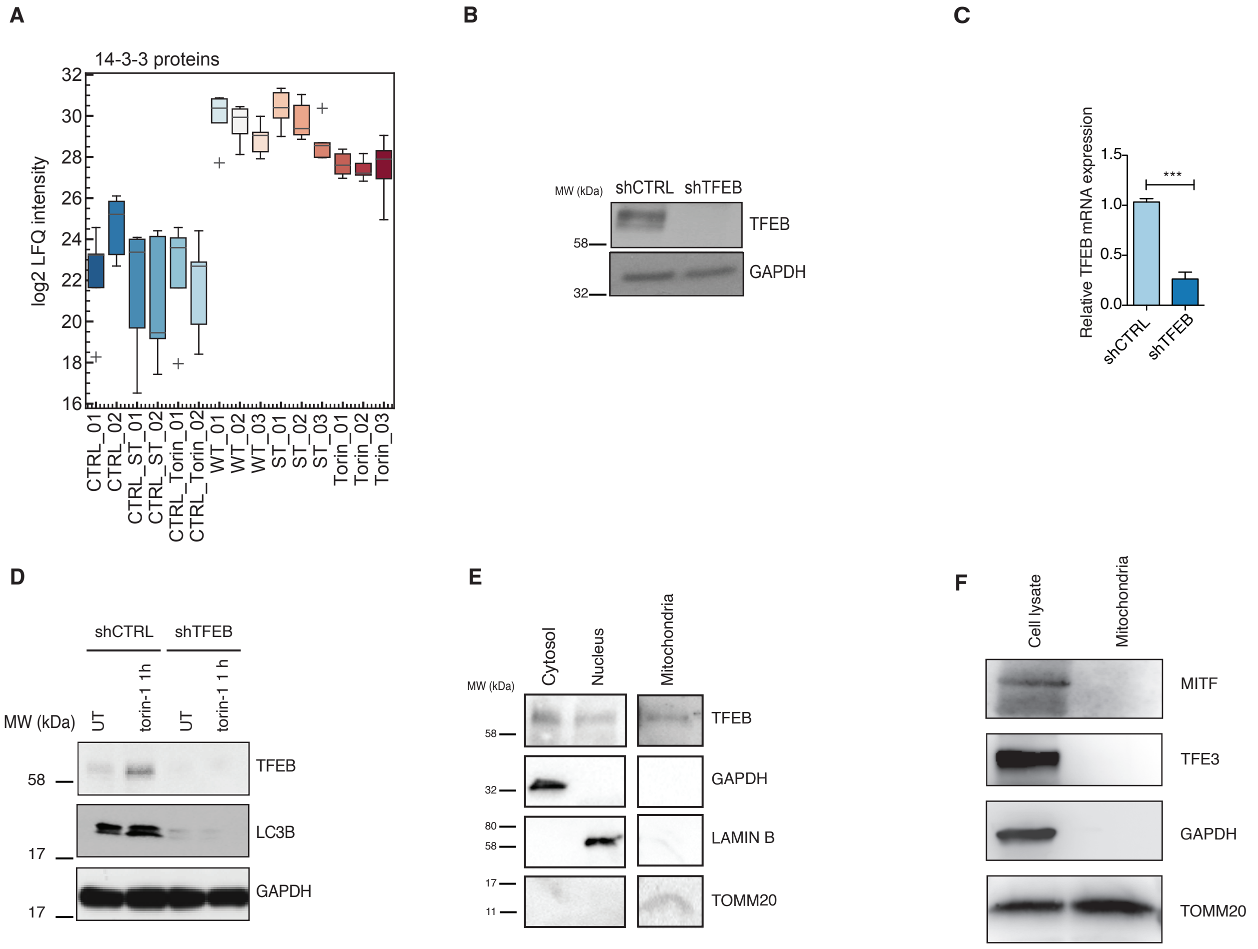

Appendix Figure S1.
(A) Boxplot of 14-3-3 proteins in the core TFEB interactome. Data represent the means ± S.E.M. The central band of each box is the median value, and the box defines the 25th (lower) and 75th (higher) quantile. The whiskers represent the minimum and maximum value in the data excluding outliers (> 1.5 * inter quantile range distance to median—indicated by a cross). Data are from 3 biological replicates.
(B TFEB and GAPDH Western Blot
(C) TFEB mRNA expression (normalized to HPRT) in control (shCTRL) and TFEB-depleted (shTFEB) HeLa cells. Western blot and qPCR are representative of three independent experiments showing similar results. Shown are mean ± SEM, n=3 biological replicates. unpaired t-test was conducted, and statistical significance is denoted as ***p<0.005.
(D) LC3B and endogenous TFEB levels in shCTRL and shTFEB cells in Untreated conditions (UT) and upon torin-1 treatment for 1 hour (1 h). The experiment was repeated independently three times showing similar results.
(E) TFEB localization in subcellular fractions isolated from human monocyte derived macrophages. LAMINB, TOMM20, and
GAPDH served as controls for nucleus, mitochondria, and cytoplasm, respectively. Western blot is representative of two independent experiments showing similar results.
(F) MITF and TFE3 expression in 10% of total lysate (positive control) and in highly enriched mitochondria isolated from HeLA cells using TOMM22-magnetic beads.
TOMM20 was used to confirm the enrichment of mitochondria.

Appendix Fig. S2

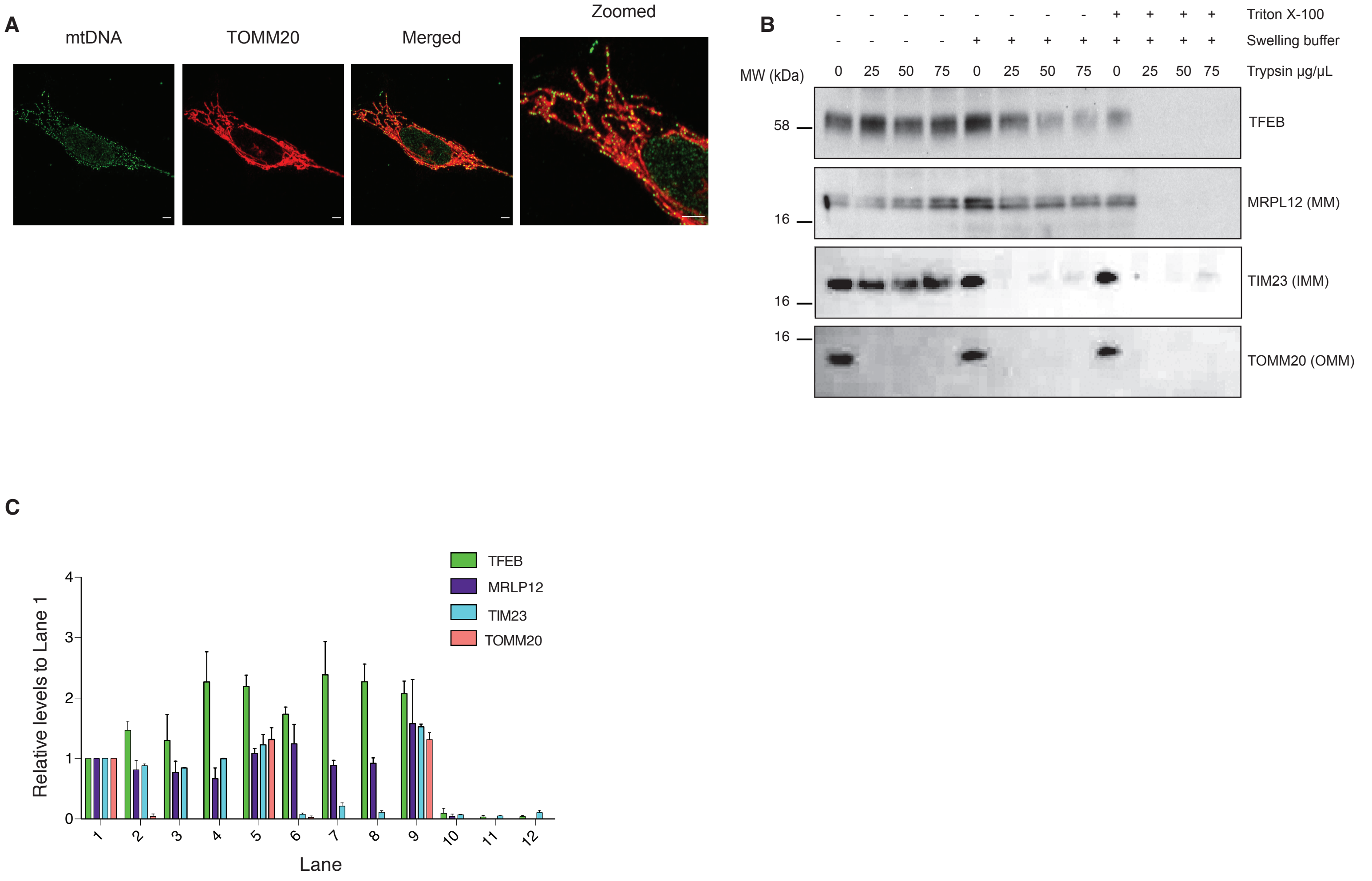

Appendix Figure S2. (A) mtDNA and TOMM20 staining in HeLa cells to confirm mitochondrial localization of mtDNA. Scale bar = 10 μm.

(B) Protease protection assay on mitochondria isolated from HEK293T cells after treatment with increasing concentrations of trypsin (0 – 75 μg) in the presence or absence of swelling buffer. Samples were analyzed for the outer mitochondrial membrane (OMM) protein TOMM20, the inner mitochondrial membrane (IMM), protein TIM23, and the mitochondrial matrix (MM) protein MRPL12. MRPL12 and endogenous TFEB were exposed to trypsin after mitochondria were lysed with Triton X-100. The experiment was repeated twice with similar results.

(C) Densitometric analysis of protein levels in (B). Band intensities were normalized to the first lane (untreated mitochondria) of the respective protein. Data represents 2 biological replicates and error bars denote mean ± SEM.

Appendix Fig S3

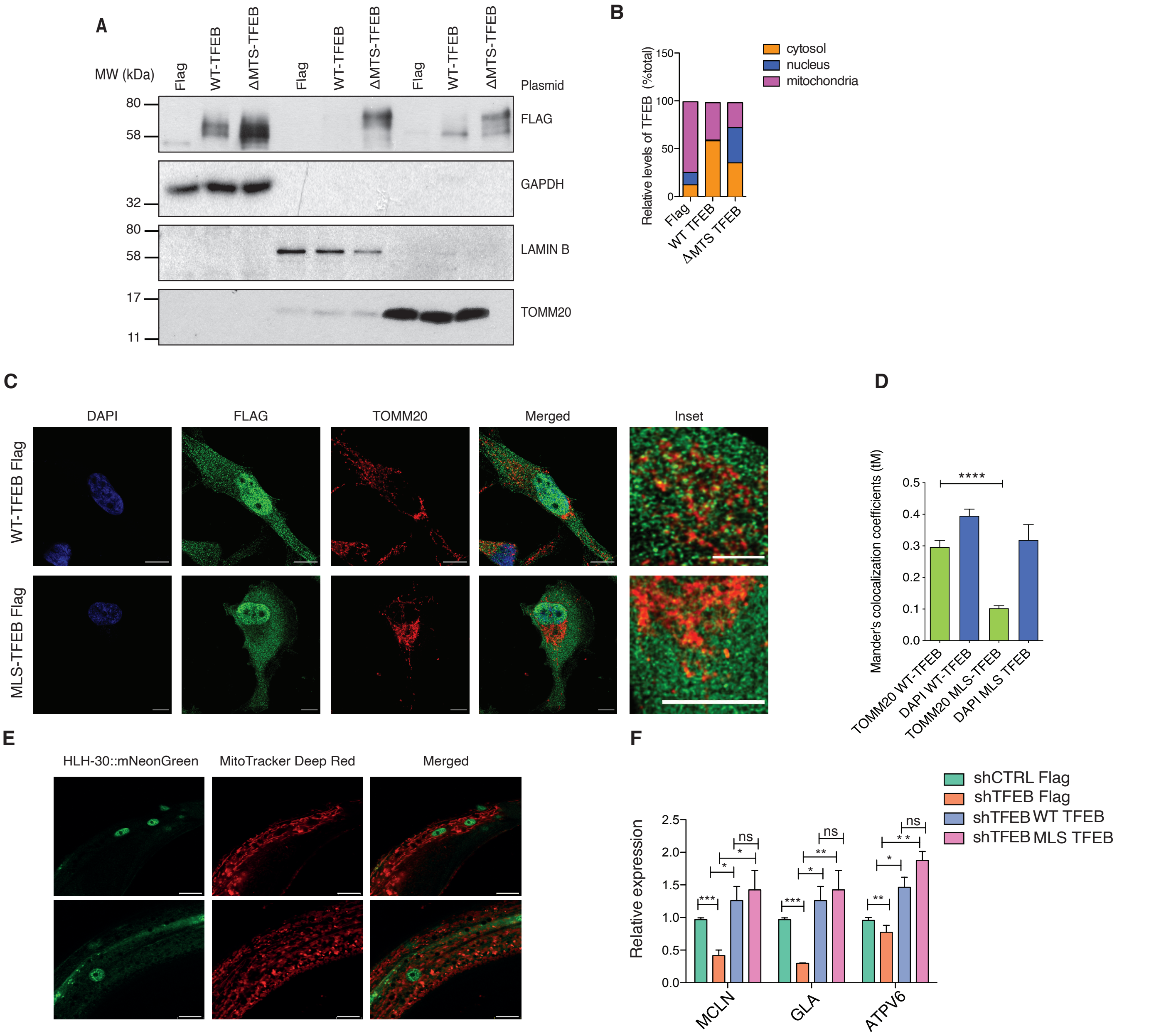

Appendix Figure S3. (A) Analysis of subcellular fractions isolated from cells expressing FLAG alone, FLAG-tagged WT-TFEB or FLAG-tagged ΔMTS-TFEB. LAMINB, TOMM20 and GAPDH served as controls for nucleus, mitochondria and cytosol, respectively. Relative abundance of TFEB in the various fractions. The experiment was repeated three times with similar results.

(B) Relative abundance of TFEB in the various fractions of the immunoblot shown in (A). Data represents 2 technical replicates.

(C) TOMM20 staining in HeLa cells transfected with FLAG-tagged WT-TFEB or FLAG-tagged MLS-TFEB. Scale bar= 10 um.

(D) Ten cells per group were analyzed. Colocalization was evaluated in ROIs (25x25 pixels) as described in Methods section. Green bars show the Mander's colocalization coefficients using the calculated thresholds (tM) for the green (FLAG) and the red (TOMM20) channels. Blue bars show the Mander's colocalization coefficients using the calculated thresholds (tM) for the green (FLAG) and the blue (DAPI) channels. Shown are mean ± SEM, data represents 4 biological replicates. Unpaired t-test was conducted and statistical significance is denoted as ****$p<0.0001$.

(E) HLH-30::mNeonGreen expressed in muscle and hypodermal cells of C. elegans (wild type, day 1 adult) and mitochondria stained with MitoTracker Deep Red. Scale bar = 10um.

(F) mRNA levels of TFEB target genes (relative to HPRT) from shCTRL and shTFEB transfected with FLAG, WT-TFEB- FLAG or MLS-TFEB-FLAG. Shown are mean ± SEM, n=3 biological replicates. Unpaired t-test was conducted between each group, and statistical significance is denoted as *$p<0.05$; **$p<0.01$; ***$p<0.005$.

Appendix Fig S4

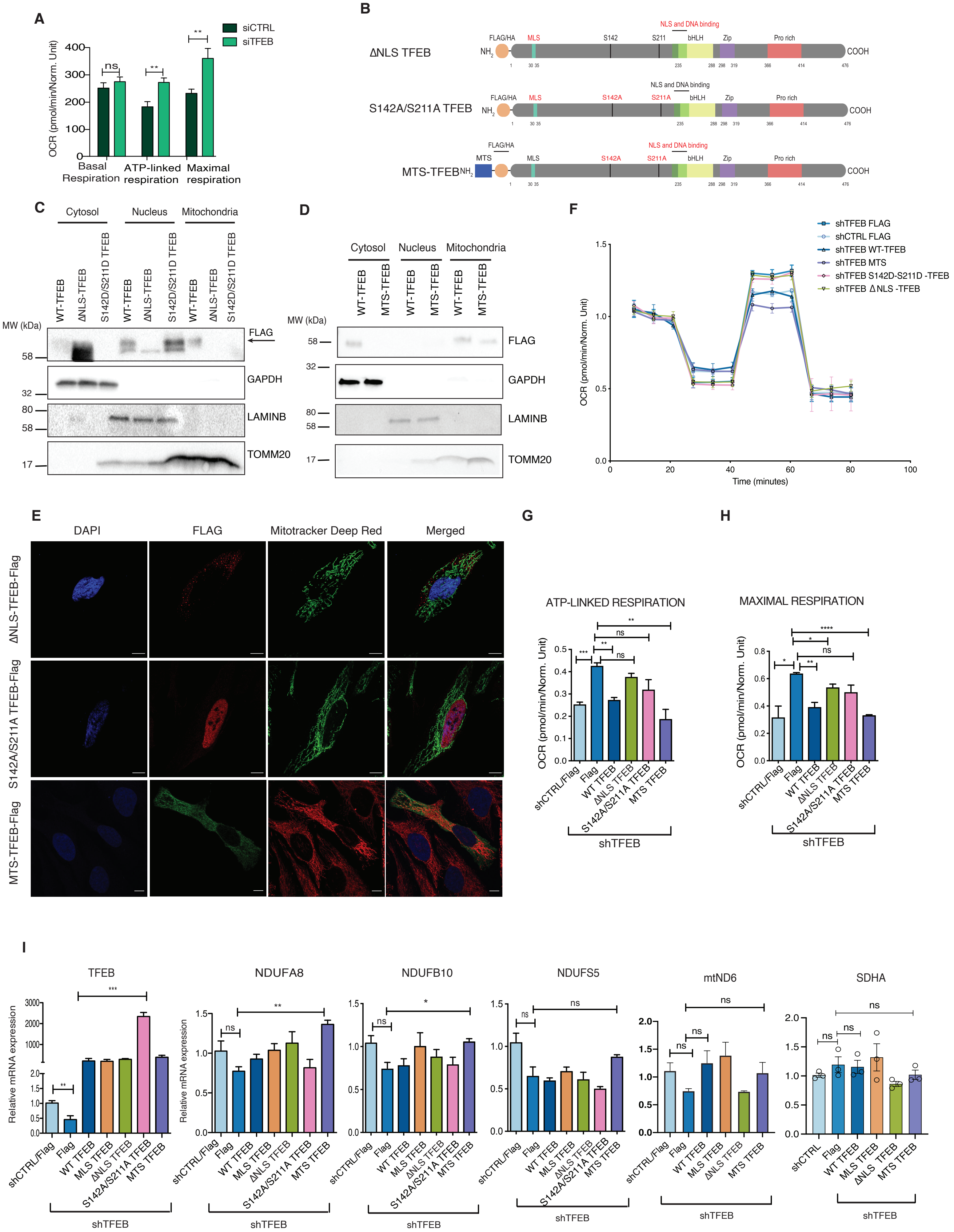

Appendix Figure S4. (A) Basal respiration rate, ATP-linked respiration and Maximal respiration in HEK293T transiently transfected with control siRNA (siCTRL), siRNA against TFEB (siTFEB). Shown are mean ± SEM, n=3 biological replicates. Unpaired t-test was done, and statistical significance represent as $^{**}p<0.01$.

(B) Schematic representation of the ΔNLS-TFEB FLAG plasmid in which the basic residues (R245–R248) were mutated to alanine, to prevent TFEB nuclear translocation; and the MLS motif was mutated to prevent TFEB mitochondrial translocation; S142A/S211A-TFEB FLAG plasmid (in which the Ser142 and Ser211 residues were mutated to alanine, to prevent TFEB cytosolic retention; and the MLS motif was mutated to prevent TFEB mitochondrial translocation); MTS-TFEB FLAG plasmid (in which MTS sequence of SOD is tagged).

(C) Western Blot of subcellular fractions isolated from cells expressing FLAG-tagged WT-TFEB or FLAG-tagged ΔNLS-TFEB or FLAG-tagged S142A/S211A-TFEB. LAMINB, TOMM20 and GAPDH served as controls for nucleus, mitochondria and cytoplasm, respectively. The experiment was repeated two times with similar results.

(D) Western Blot of subcellular fractions isolated from cells expressing FLAG-tagged WT-TFEB or FLAG-tagged MTS-TFEB. The experiment was repeated two times with similar results.

(E) HeLa cells transfected with FLAG-tagged ΔNLS-TFEB, FLAG-tagged S142A/S211A-TFEB and FLAG-tagged MTS-TFEB were stained using anti-FLAG and anti-TOMM20. Scale bar= 10 μm.

(F) OXPHOS profile normalized to Basal respiration of shCTRL cells and shTFEB cells transfected with FLAG, WT-TFEB FLAG, MLS-TFEB FLAG, ΔNLS-TFEB FLAG and S142A/S211A-TFEB FLAG.

(G, H) ATP-linked respiration and **(H)** Maximal respiration normalized to Basal respiration, in shCTRL and shTFEB cells transfected with FLAG, WT-TFEB FLAG, ∆NLS-TFEB FLAG, S142A/S211A-TFEB FLAG or MTS-TFEB FLAG. Shown are mean ± SEM, n=3 biological replicates. Unpaired t-test was done, and statistical significance represent as *p<0.05; **p<0.01; ***p<0.005; ****p<0.001.

(I) mRNA levels of TFEB and mitochondrial genes NDUFA8, NDUFB10, NDUFS5 and mtND6 (relative to HPRT) from shTFEB HeLa cells transfected with FLAG, WT-TFEB-FLAG, MLS-TFEB-FLAG, ∆NLS-TFEB FLAG, S142A/S211A-TFEB FLAG or MTS-TFEB FLAG. Shown are mean ± SEM, n=3 biological replicates.

Unpaired t-test was done, and statistical significance represent as *p<0.05; **p<0.01; ***p<0.005.

Appendix Fig. S5

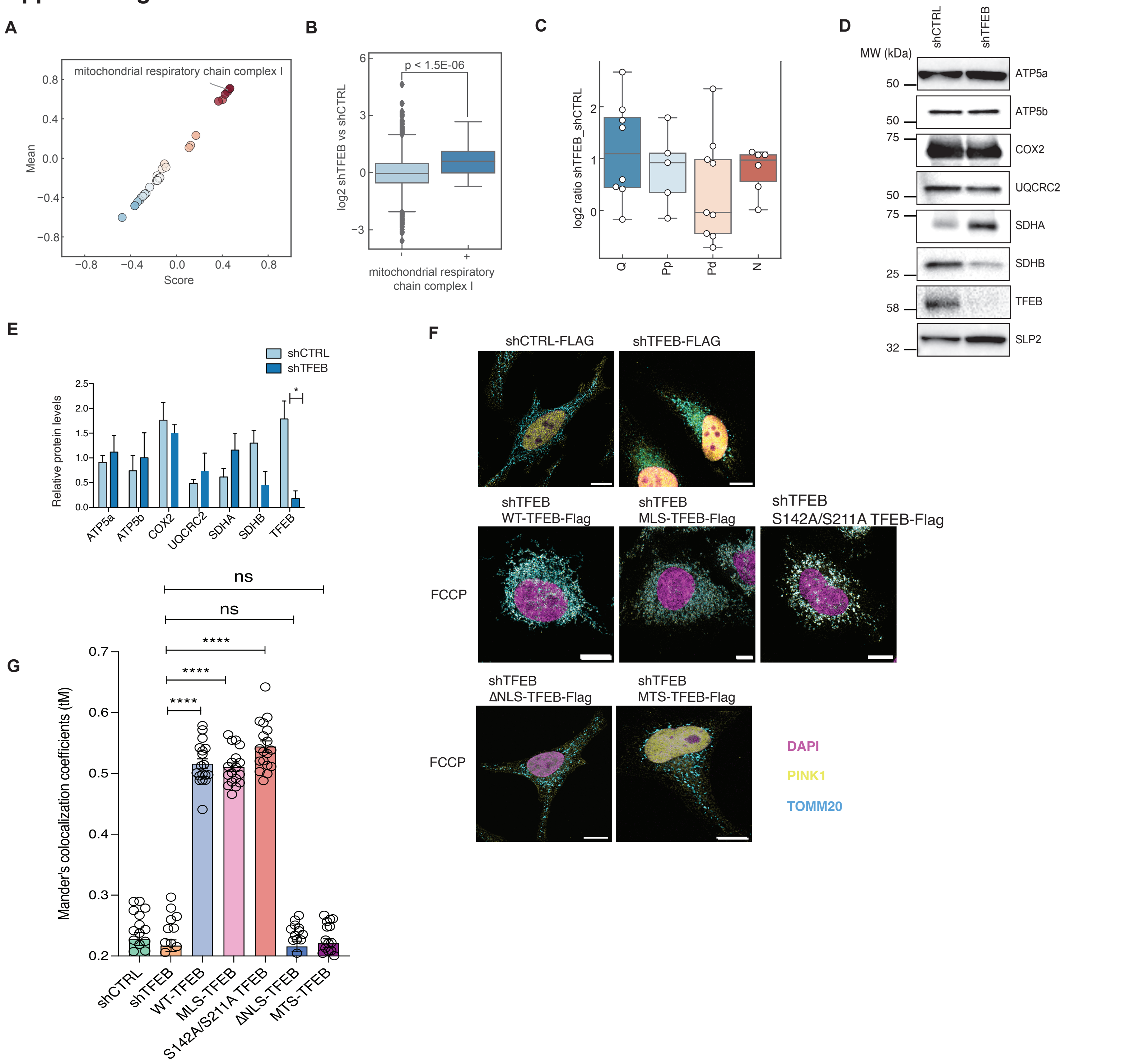

Appendix Figure S5. (A) 1D enrichment of the whole proteome analysis in shCTRL versus shTFEB cells. Proteins were annotated by the Gene Ontology term.

(B) log2 fold change of all detected proteins and proteins associated with Complex I in shCTRL and shTFEB-depleted HeLa cells. The central band of each box is the median value and the box defines the 25th (lower) and 75th (higher) quantile. The whiskers represent the minimum and maximum value in the data excluding the outliers. A Mann-Whitney U-test was applied and the p value is indicated in the figure.

(C) Boxplot and individual points represent proteins of specific complex I modules. Raw data points are indicated by the white circles for each complex module.

(D) ATP5a, ATP5b, COX2, UQCRC2, SDHA, SDHB, TFEB, and SLP2 expression in shCTRL and shTFEB cells.

(E) Mean densitometric analysis of Western blot shown in Figure S5D. Data shown is representative of two independent experiments showing similar results. Error bars indicate SEM.

(F) Confocal microscopy image of shCTRL and shTFEB HeLa cells stained for PINK1 and TOMM20. Scale bar = 10uM

(G) Graph shows colocalization coefficients using the calculated thresholds (tM) for PINK channel. One way ANNOVA followed by Tukey multiple comparison test was done to determine statistical significance and represented as ****p<0.001. Error bar indicate SEM. Data presented is from 3 biological replicates.

Appendix Fig S6

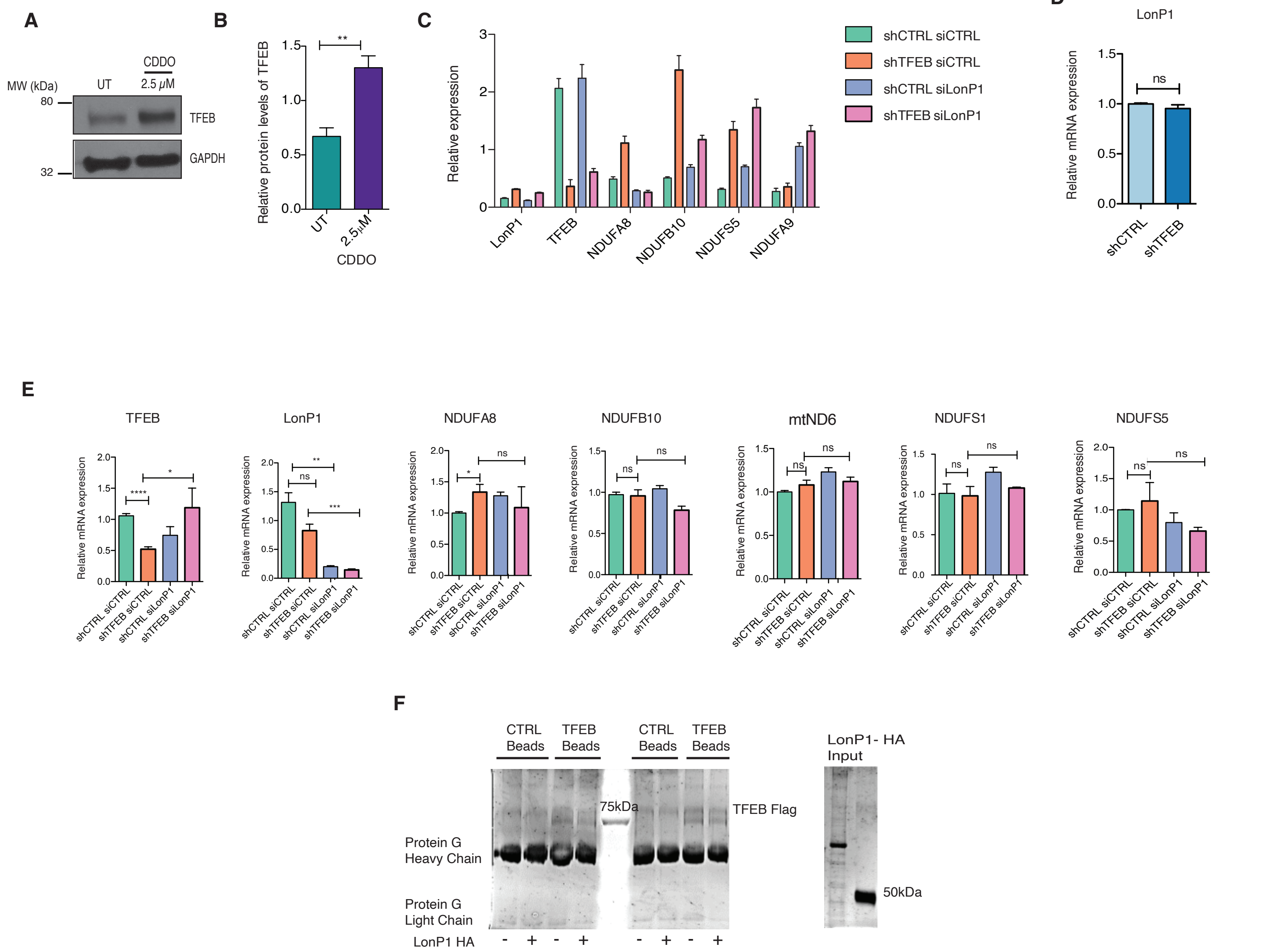

Appendix Figure S6. (A) Immunoblot of TFEB and GAPDH upon treatment with the LONP1 inhibitor CDDO.

(B) Mean densitometric analysis of the immunoblot from S6A. Shown are mean ± SEM. The data represents 3 biological replicates. **p<0.01.

(C) Mean densitometric analysis of the immunoblot from Figure 6C. Shown are mean ± SEM. The experiment was repeated two times with similar results.

(D) mRNA expression of LONP1 (relative to HPRT) in shCTRL and shTFEB cells. Shown are mean ± SEM, n=3 biological replicates. ns indicates statistically not significant p>0.05.

(E) mRNA expression of TFEB, LONP1, NDUFA8, NDUFB10, mtND6, NDUFS1 and NDUFS5 (relative to RPL13A) in shCTRL and shTFEB cells transfected with siCTRL and siLONP1. Shown are mean ± SEM, n=3 biological replicates with 6 technical replicates.

(F) SyproRuby staining of SDS-PAGE depicting the purity of LONP1 and TFEB protein isolation in Figure 6I.

Appendix Fig S7

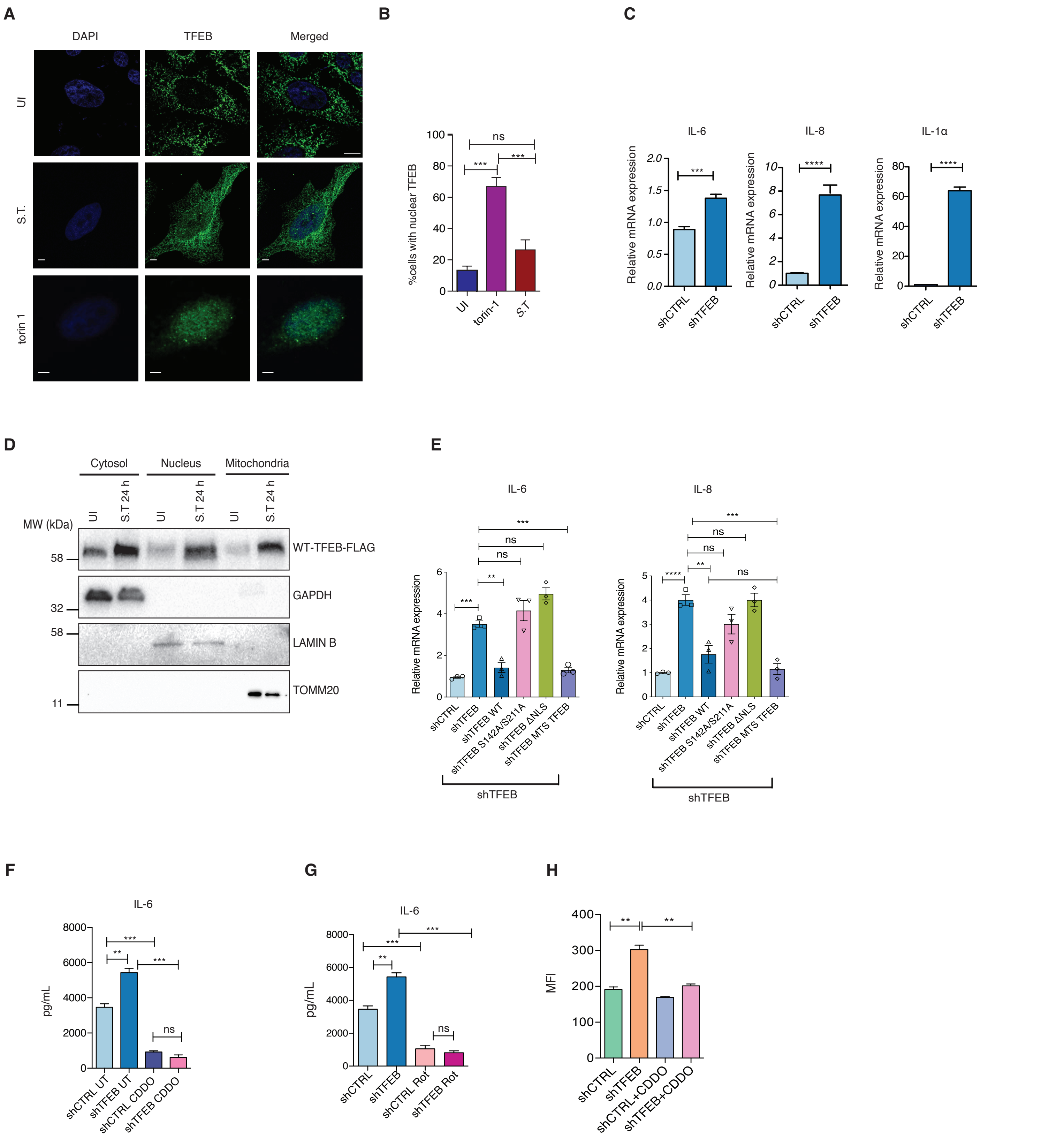

Appendix Figure S7. (A) Endogenous TFEB localization in uninfected cells (UI) *S*. Typhimurium-infected (*S*.T.) and torin-1 (30 minutes) treated HeLa cells. Scale Bar = 4 *μ*m.

(B) HeLa cells were analyzed to calculate the percentage of cells showing nuclear TFEB localization. The experiment was repeated independently three times with similar results. Shown are mean ± SEM from 3 biological replicates. Unpaired t-test was done to determine statistical significance and represented as ***p<0.005; ns p>0.05.

(C) Relative mRNA expression of IL-6, IL-8 and IL-1α (relative to RPL13A) in shCTRL and shTFEB cells. Shown are mean ± SEM, n=4 biological replicates. Unpaired t-test was applied to determine statistical significance and represented as ***p<0.005; ****p<0.001.

(D) WT-TFEB-FLAG subcellular localization upon 24 h of infection with *S.* Typhimurium infection (MOI 100) in HeLa cells. LAMINB, TOMM20, and GAPDH served as controls for nucleus, mitochondria, and cytosol, respectively. Western blot is representative of two independent experiments showing similar results.

(E) Relative mRNA levels of IL-6 and IL-8 (relative to RLP13A) in shCTRL and shTFEB transfected with FLAG, FLAG-tagged WT-TFEB, ΔNLS-TFEB or S142A/S211A-TFEB plasmids and infected for 6 h with *S.* Typhimurium (MOI 100). Shown are mean ± SEM, n=3 biological replicates. Unpaired t-test was applied to determine statistical significance and represented as ns p>0.05; **p<0.01; ***p<0.005; ****p<0.001.

(F) IL-6 expression in supernatants of shCTRL and shTFEB cells untreated (UT) and treated with 2.5 μM of CDDO infected for 24 h with *S.* Typhimurium (MOI 100). Shown are mean ± SEM, n=3 biological replicates. Unpaired t-test was done to determine statistical significance and represented as **p<0.01; ***p<0.005.

(G) IL-6 in supernatants of shCTRL and shTFEB cells untreated (UT) or treated with 500 nM of Rotenone (Rot) infected for 24 h with *S.* Typhimurium. Shown are mean ± SEM, n=3 biological replicates. Unpaired t-test was applied to determine statistical significance and represented as **$p<0.01$; ***$p<0.005$.

(H) MitoSOX-based flow cytometric detection of mitochondrial ROS production in shCTRL and shTFEB cells untreated and treated with CDDO. Shown is the Mean Fluorescence Intensity (MFI). Shown are mean ± SEM, n=3 biological replicates.

All data were subjected to unpaired t-test to determine statistical significance and represented as **$p<0.01$.
