# Supplementary figures and images for: Mitochondrial translocation of TFEB regulates complex I and inflammation (part 1 of 2)
Source: EMBO Rep. 2024 Jan 23;25(2):704–24. doi: 10.1038/s44319-024-00058-0 (PMC10897448; doi:10.1038/s44319-024-00058-0)

**Fig. 1H**

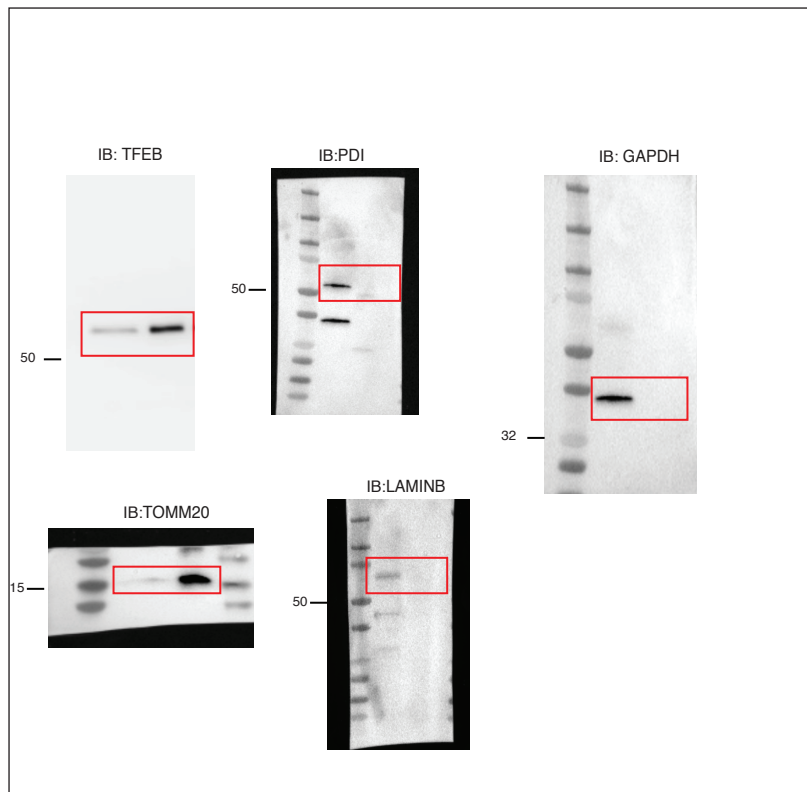

Supplement: Supplementary file 6 — Source Data Fig. 1 [file 44319_2024_58_MOESM6_ESM.zip › Fig 1 Source data/Fig 1H/Fig 1H unprocessed blots.pdf]

**Fig. 1F**

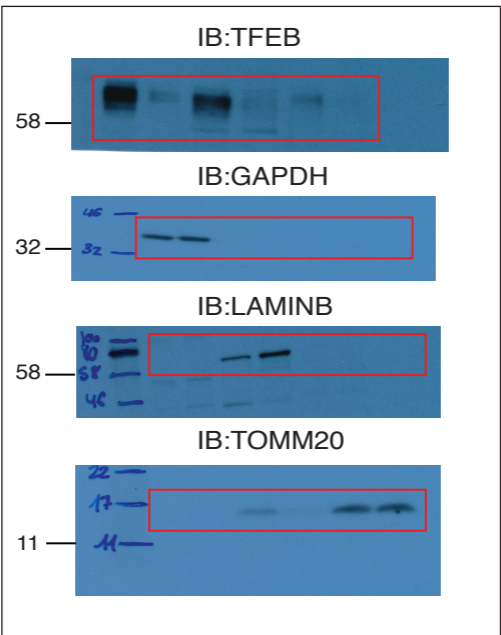

Supplement: Supplementary file 6 — Source Data Fig. 1 [file 44319_2024_58_MOESM6_ESM.zip › Fig 1 Source data/Fig 1F/Fig 1F unprocessed blots.pdf]

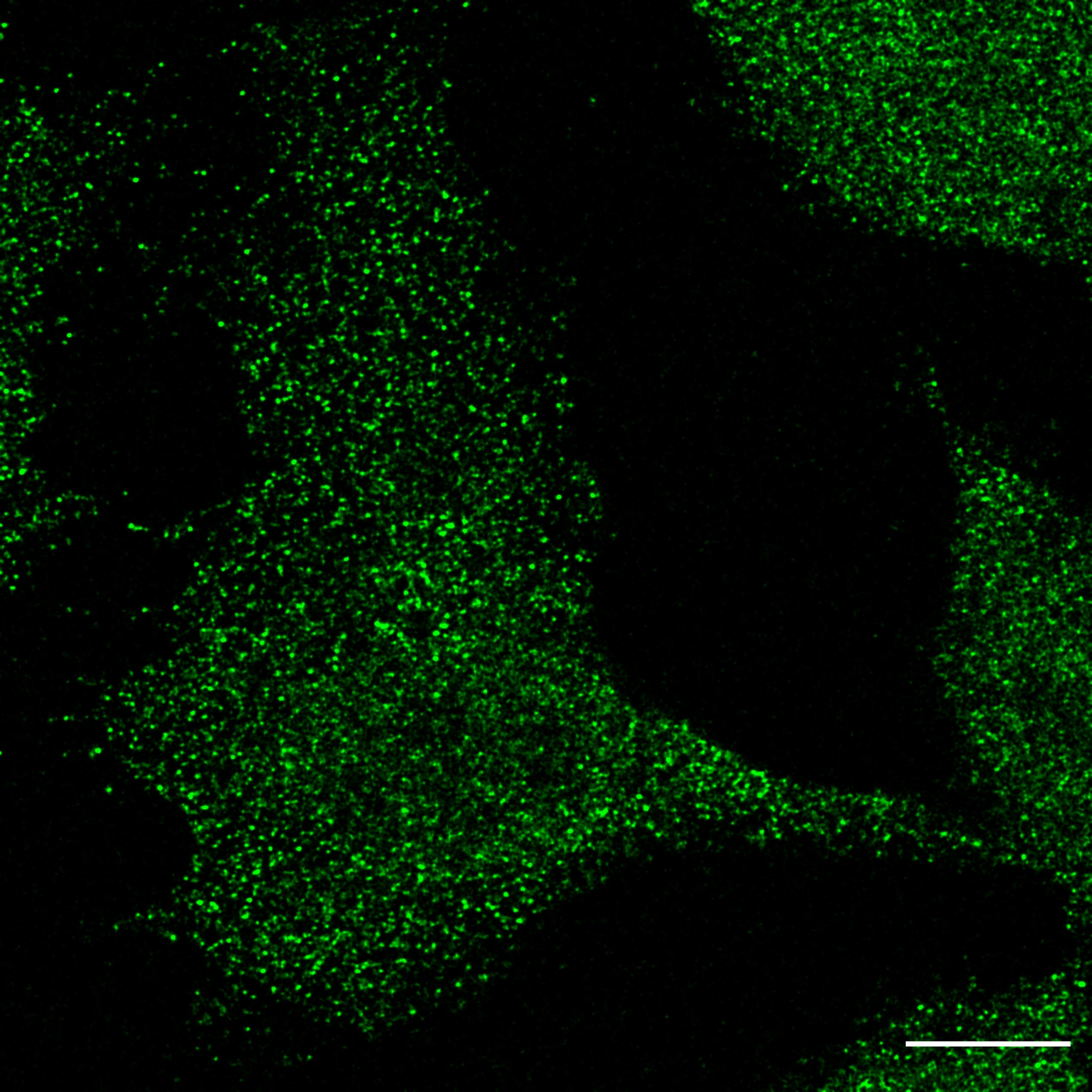

Supplement: Supplementary file 6 — Source Data Fig. 1 [file 44319_2024_58_MOESM6_ESM.zip › Fig 1 Source data/Fig 1D/Fig 1D TFEB.jpg]

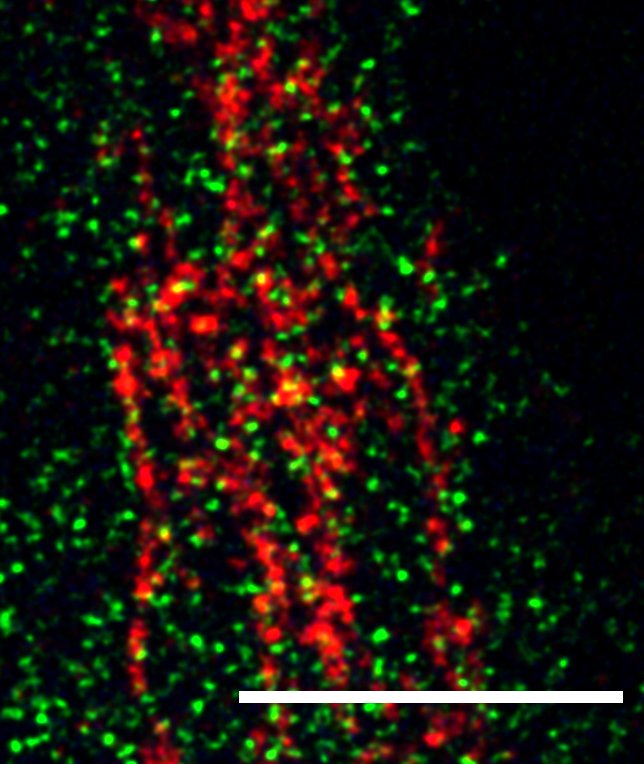

Supplement: Supplementary file 6 — Source Data Fig. 1 [file 44319_2024_58_MOESM6_ESM.zip › Fig 1 Source data/Fig 1D/Fig 1D inset.jpg]

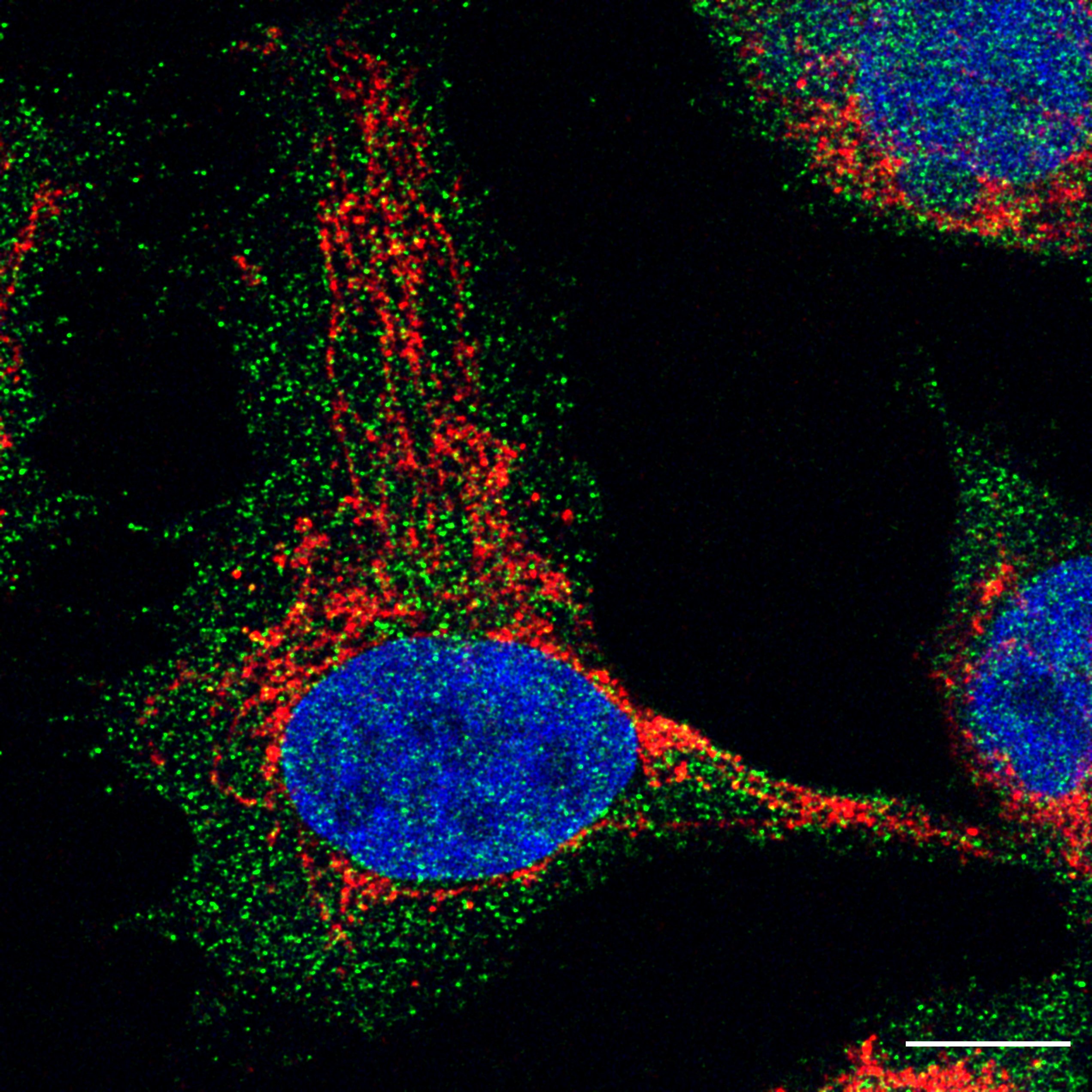

Supplement: Supplementary file 6 — Source Data Fig. 1 [file 44319_2024_58_MOESM6_ESM.zip › Fig 1 Source data/Fig 1D/Fig 1D merged.jpg]

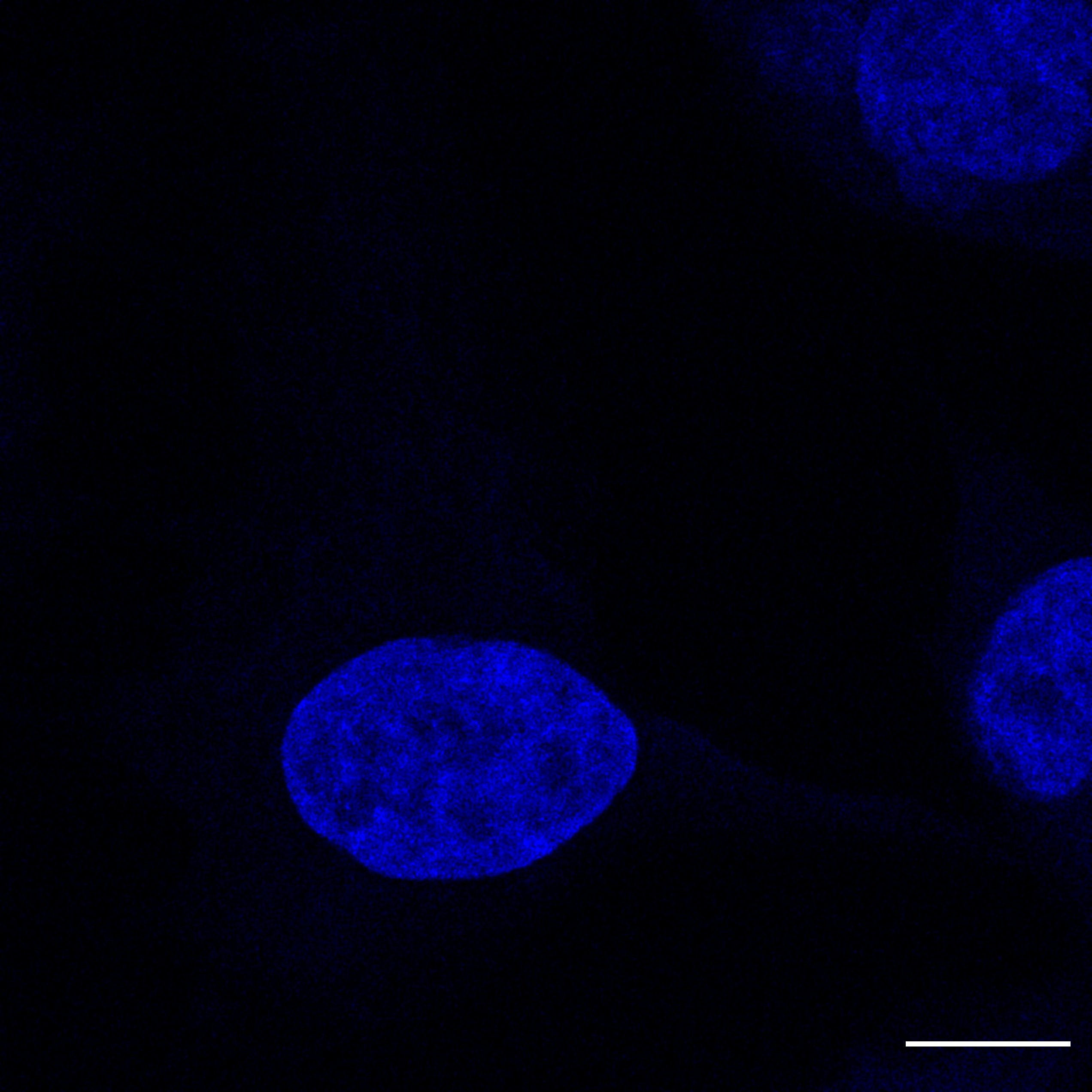

Supplement: Supplementary file 6 — Source Data Fig. 1 [file 44319_2024_58_MOESM6_ESM.zip › Fig 1 Source data/Fig 1D/Fig 1D DAPI.jpg]

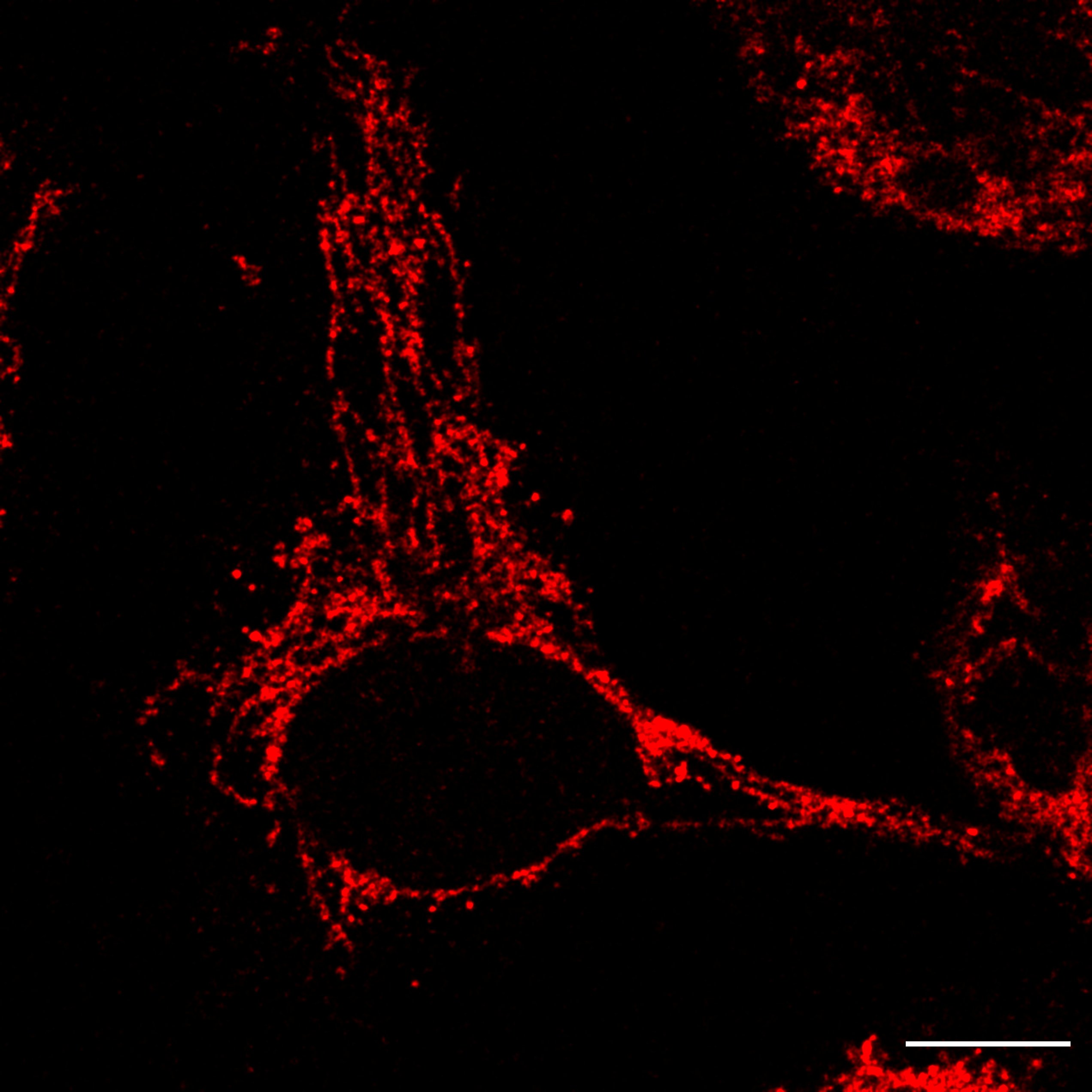

Supplement: Supplementary file 6 — Source Data Fig. 1 [file 44319_2024_58_MOESM6_ESM.zip › Fig 1 Source data/Fig 1D/Fig 1D Mitotracker.jpg]

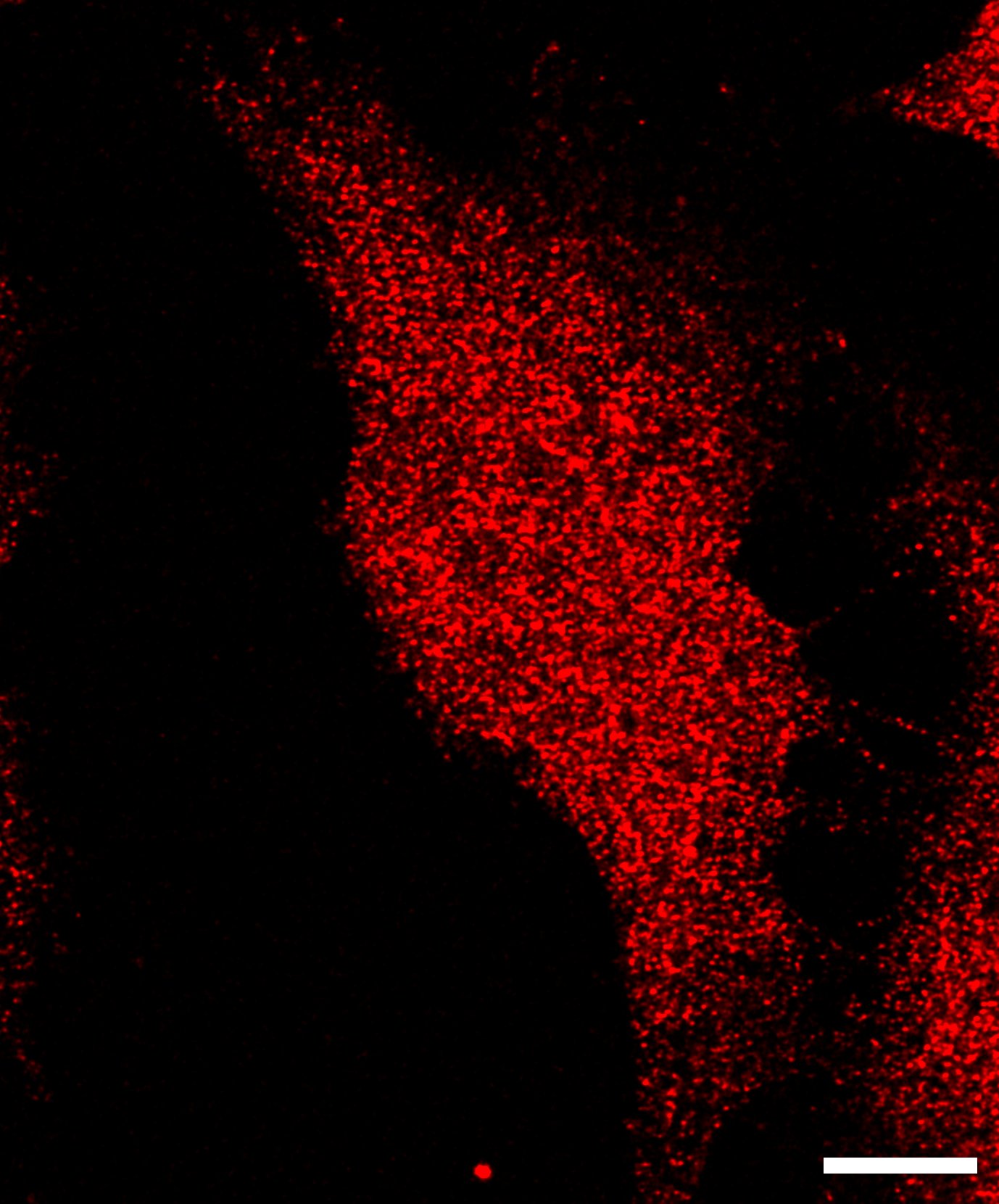

Supplement: Supplementary file 7 — Source Data Fig. 2 [file 44319_2024_58_MOESM7_ESM.zip › Fig 2 Source data/Fig 2A/Fig 2A TFEB.jpg]

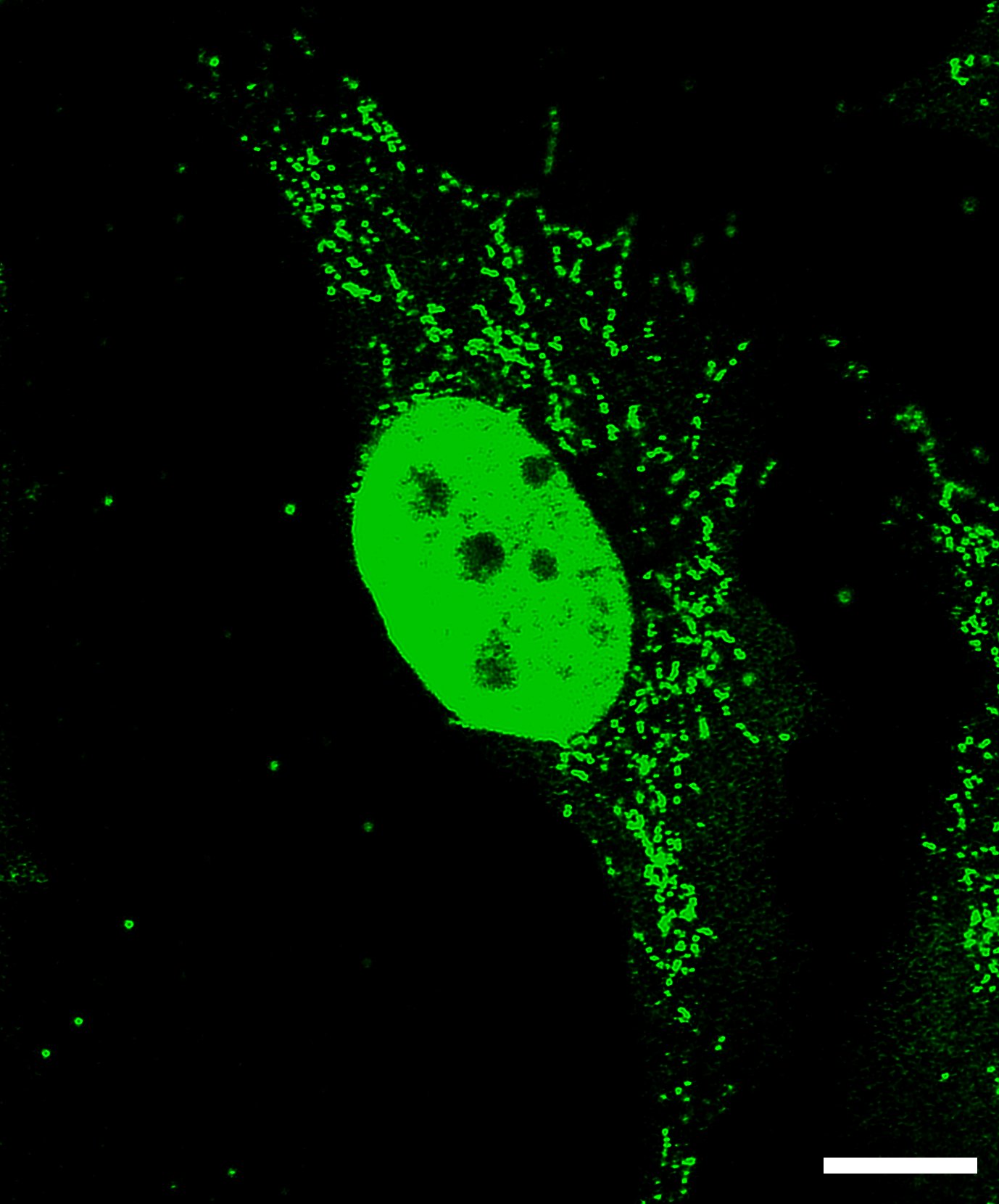

Supplement: Supplementary file 7 — Source Data Fig. 2 [file 44319_2024_58_MOESM7_ESM.zip › Fig 2 Source data/Fig 2A/Fig 2A mtdna.jpg]

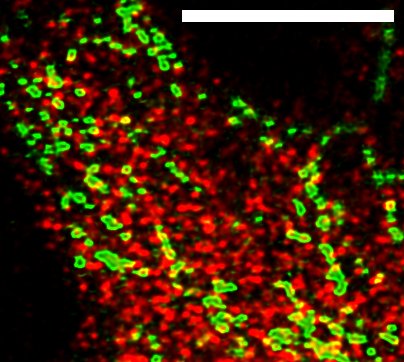

Supplement: Supplementary file 7 — Source Data Fig. 2 [file 44319_2024_58_MOESM7_ESM.zip › Fig 2 Source data/Fig 2A/Fig 2 inset.jpg]

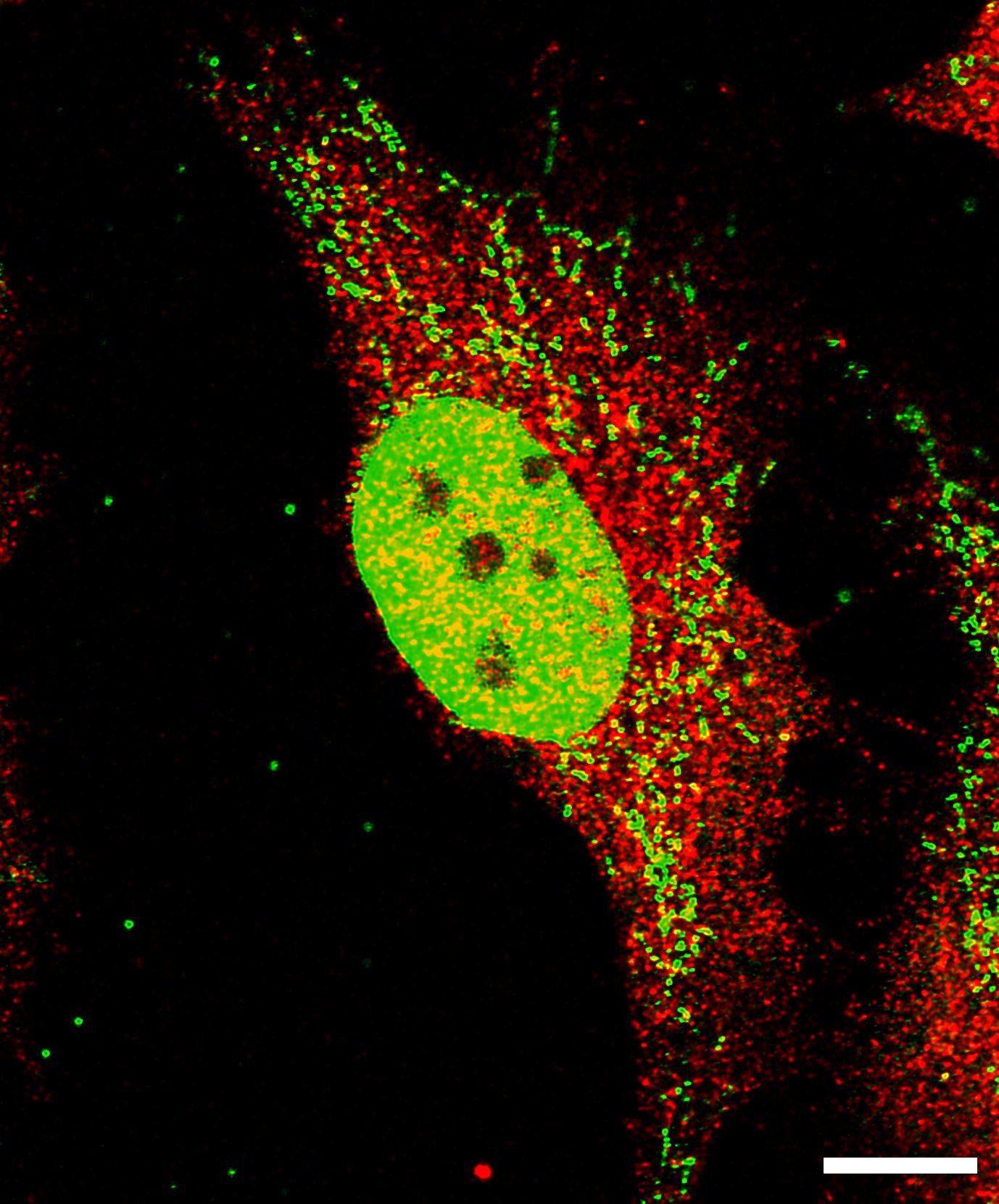

Supplement: Supplementary file 7 — Source Data Fig. 2 [file 44319_2024_58_MOESM7_ESM.zip › Fig 2 Source data/Fig 2A/Fig 2 Merged.jpg]

**Fig. 2B**

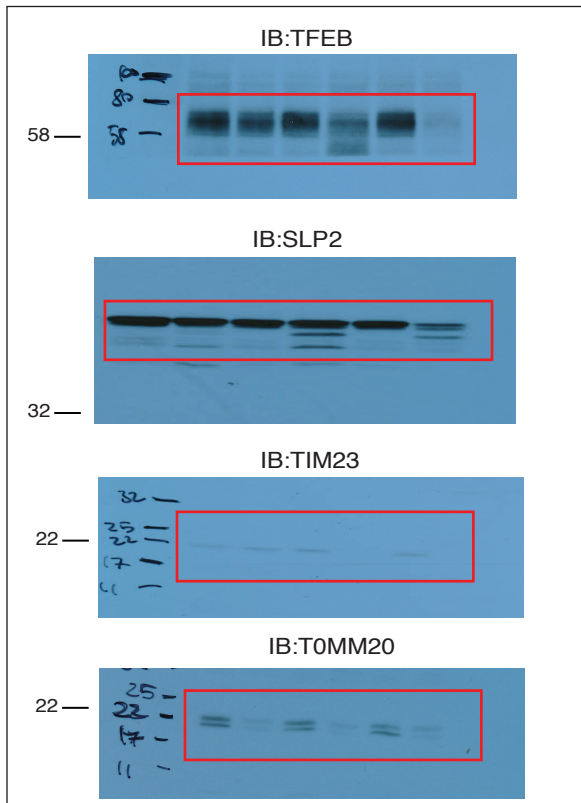

Supplement: Supplementary file 7 — Source Data Fig. 2 [file 44319_2024_58_MOESM7_ESM.zip › Fig 2 Source data/Fig 2B/Fig 2B Unprocessed Blots.pdf]

**Fig. 3D**

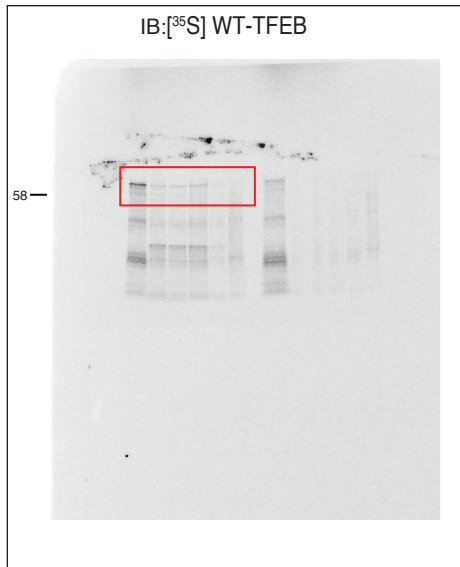

**Fig. 3D**

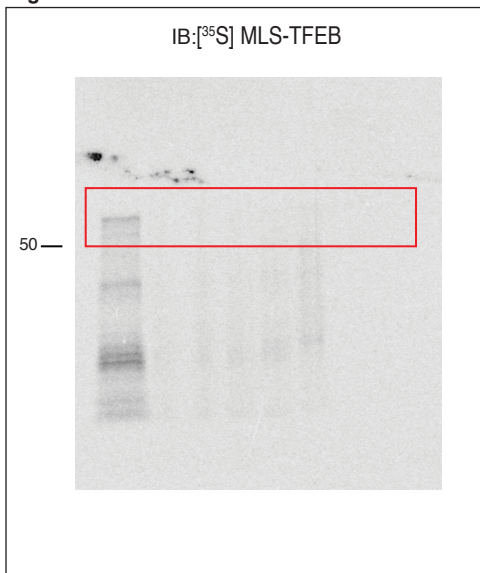

Supplement: Supplementary file 8 — Source Data Fig. 3 [file 44319_2024_58_MOESM8_ESM.zip › Fig 3 Source data/Fig 3D/Fig 3D unprocessed blots.pdf]

**Fig. 3G**

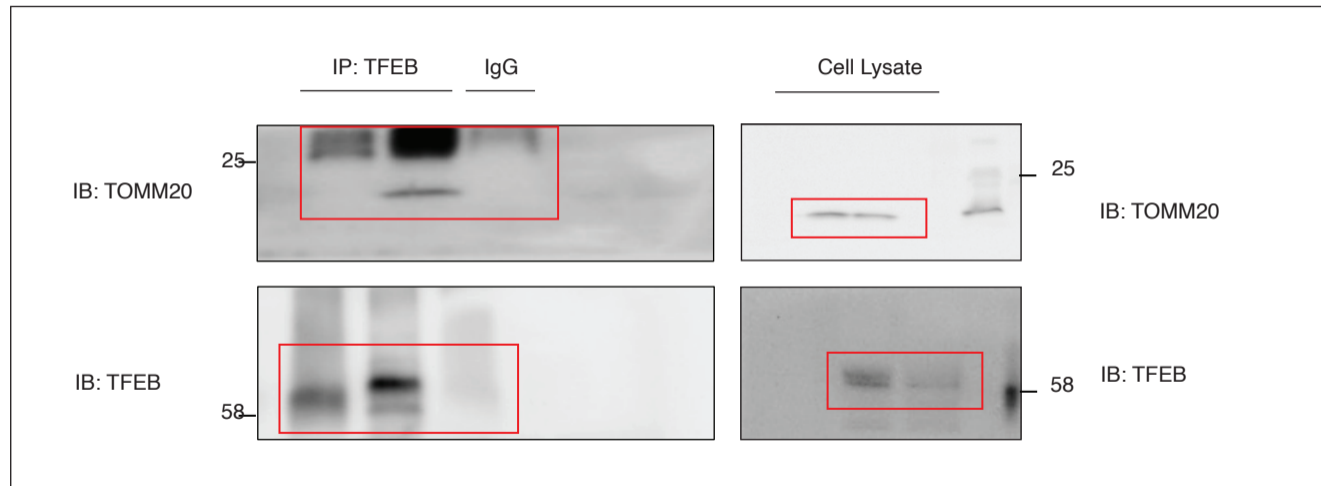

Supplement: Supplementary file 8 — Source Data Fig. 3 [file 44319_2024_58_MOESM8_ESM.zip › Fig 3 Source data/Fig 3L/Fig 3L unprocessed blots.pdf]

**Fig. 3B**

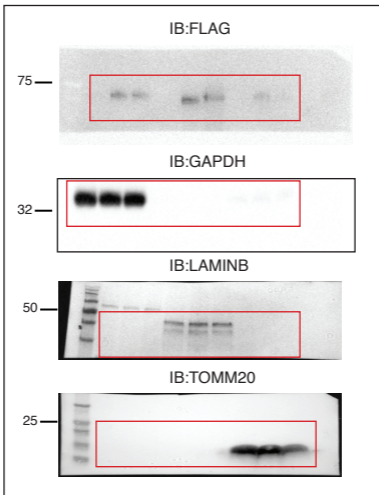

Supplement: Supplementary file 8 — Source Data Fig. 3 [file 44319_2024_58_MOESM8_ESM.zip › Fig 3 Source data/Fig 3B/Fig 3B Unprocessed blots.pdf]

**Fig. 3G**

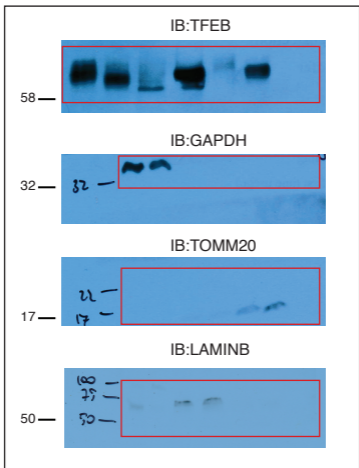

Supplement: Supplementary file 8 — Source Data Fig. 3 [file 44319_2024_58_MOESM8_ESM.zip › Fig 3 Source data/Fig 3G/Fig 3G unprocessed blots.pdf]

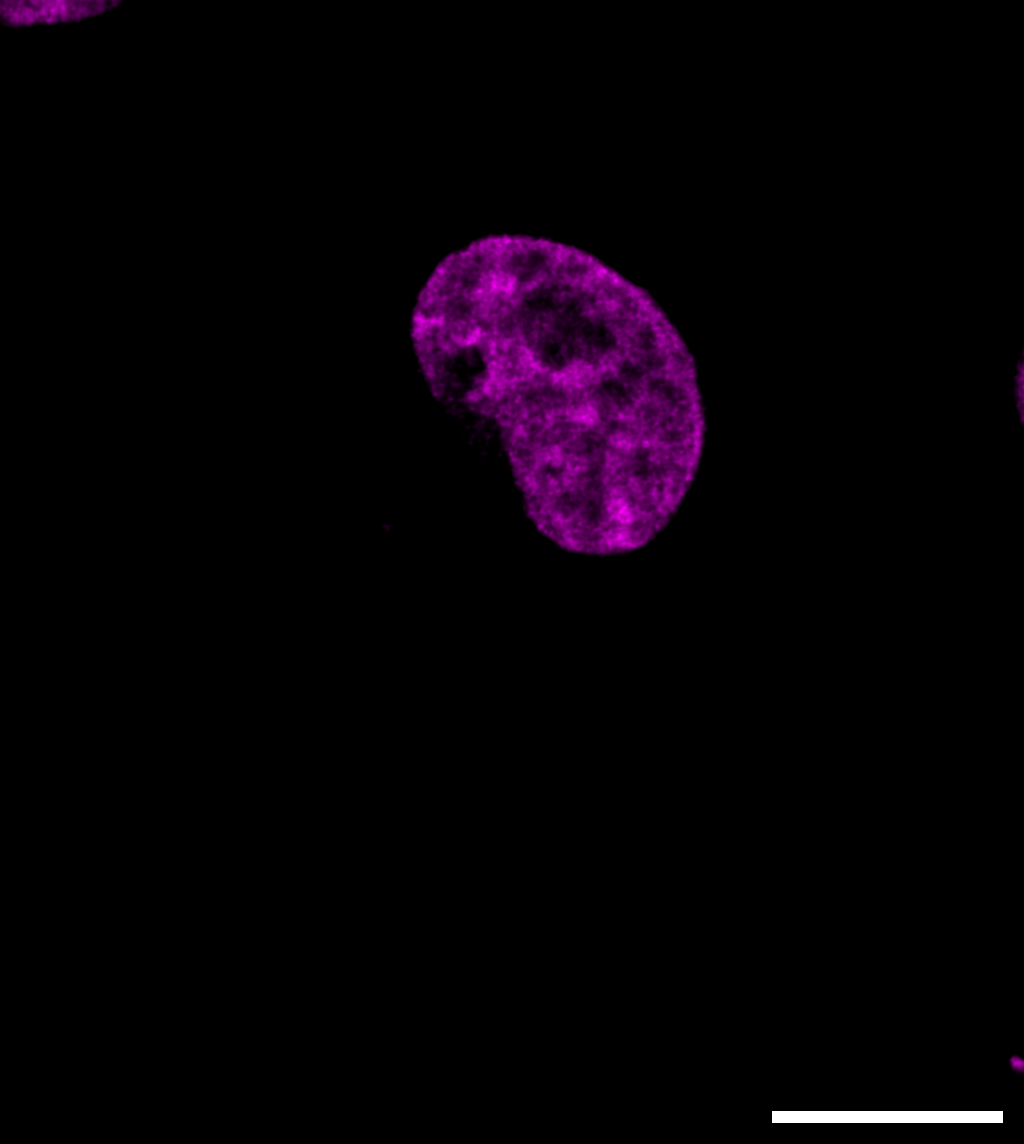

Supplement: Supplementary file 8 — Source Data Fig. 3 [file 44319_2024_58_MOESM8_ESM.zip › Fig 3 Source data/Fig 3I/Fig 3I - untreated - DAPI.png]

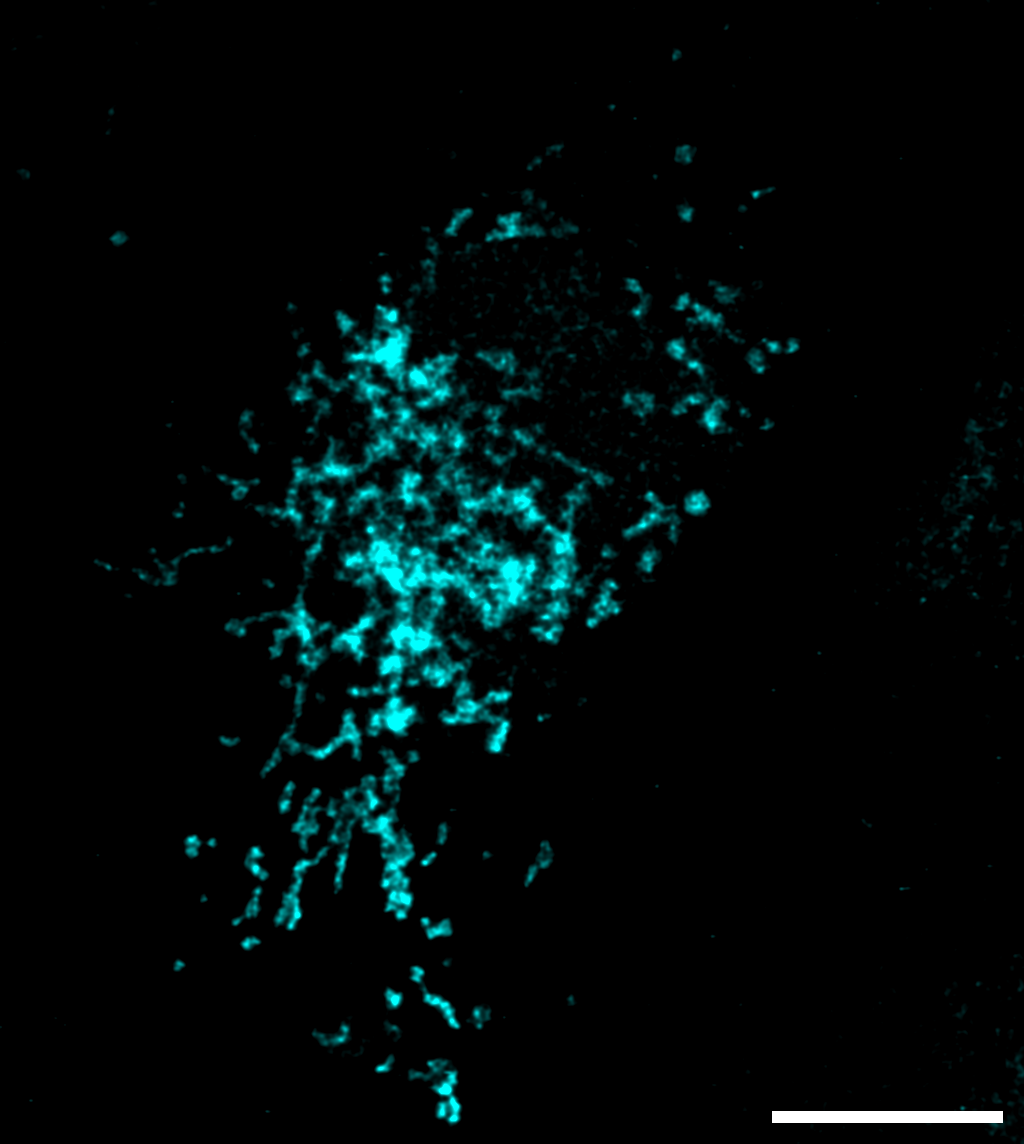

Supplement: Supplementary file 8 — Source Data Fig. 3 [file 44319_2024_58_MOESM8_ESM.zip › Fig 3 Source data/Fig 3I/Fig 3I - untreated -TOMM20.png]

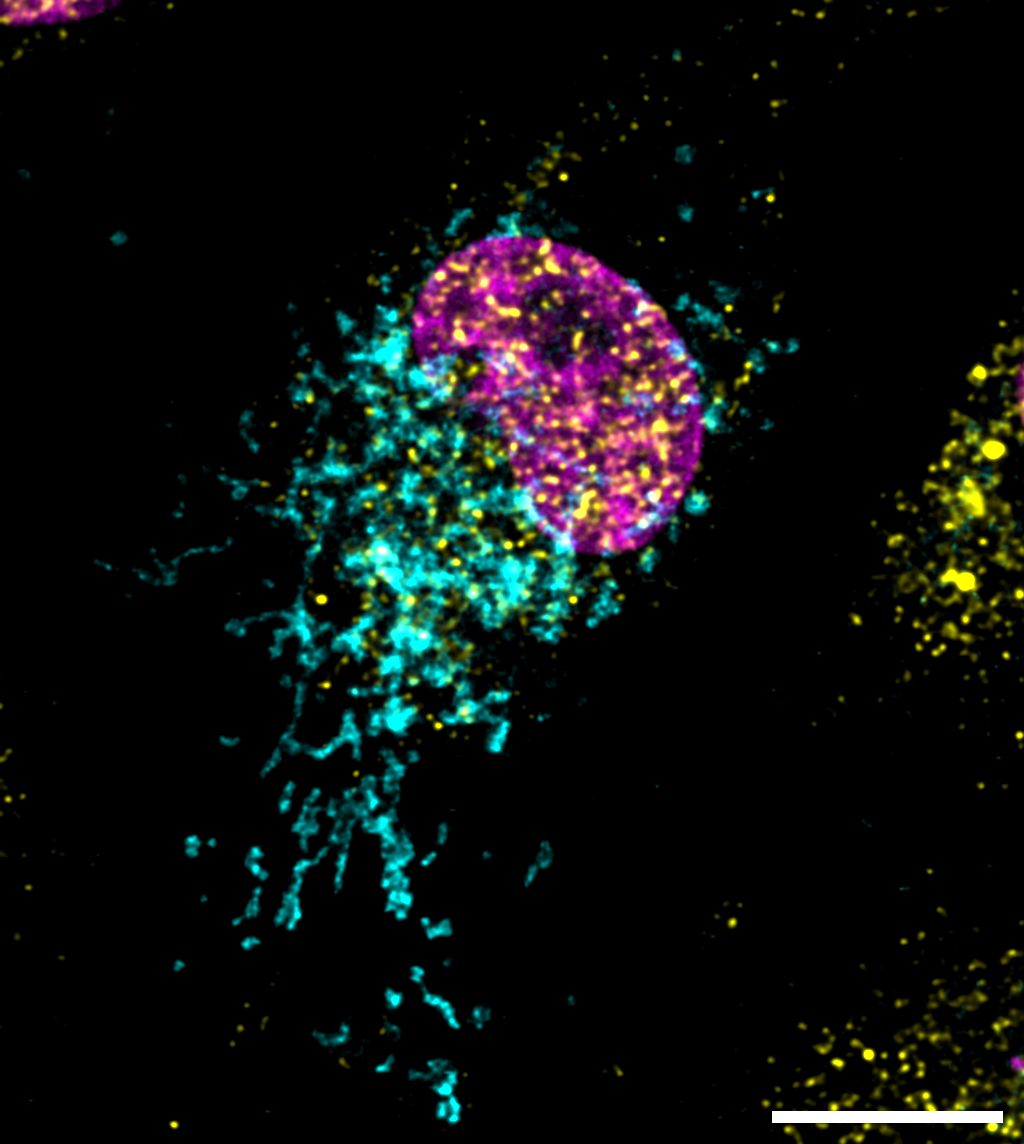

Supplement: Supplementary file 8 — Source Data Fig. 3 [file 44319_2024_58_MOESM8_ESM.zip › Fig 3 Source data/Fig 3I/Fig 3I - untreated -merged.png]

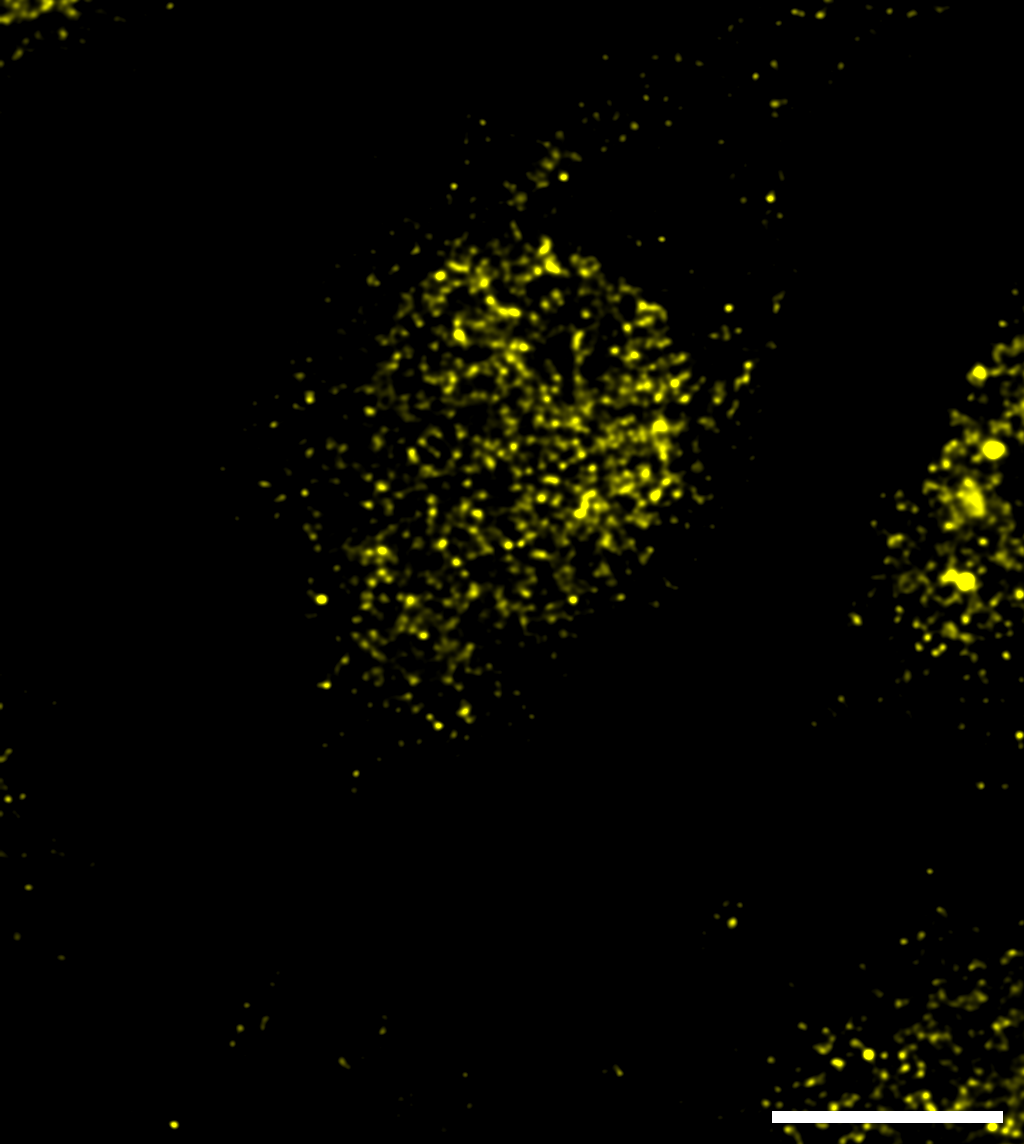

Supplement: Supplementary file 8 — Source Data Fig. 3 [file 44319_2024_58_MOESM8_ESM.zip › Fig 3 Source data/Fig 3I/Fig 3I - untreated - TFEB.png]

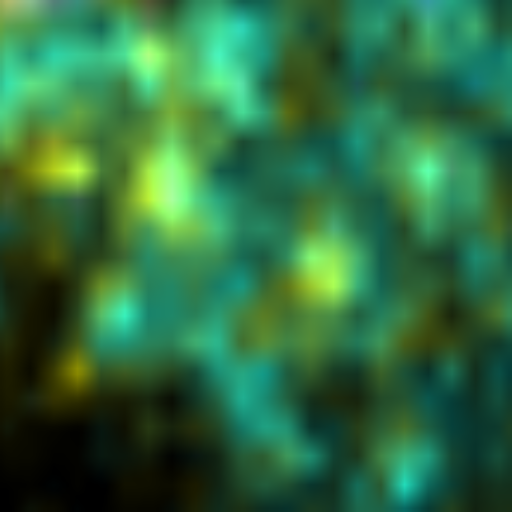

Supplement: Supplementary file 8 — Source Data Fig. 3 [file 44319_2024_58_MOESM8_ESM.zip › Fig 3 Source data/Fig 3I/Fig 3I - untreated - inset.png]

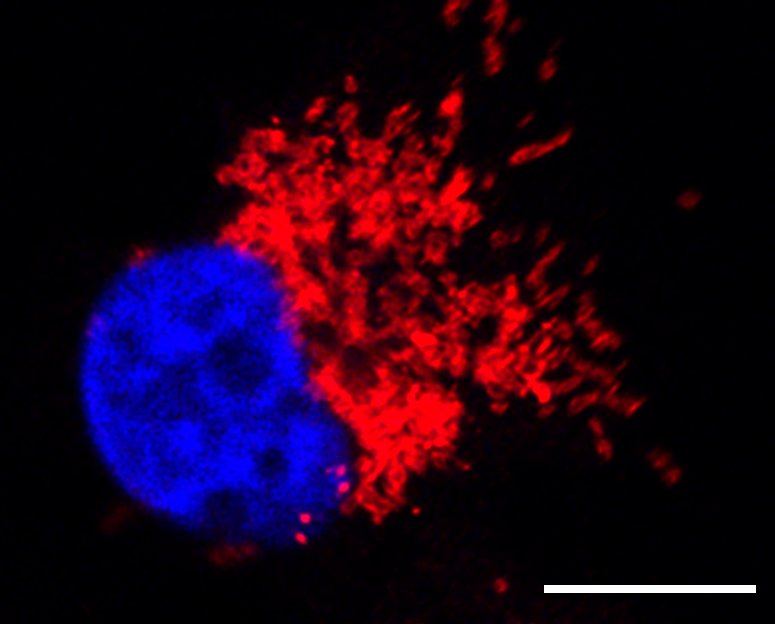

Supplement: Supplementary file 9 — Source Data Fig. 4 [file 44319_2024_58_MOESM9_ESM.zip › Fig 4 Source data/Fig 4C/Fig 4C shTFEBFLAG - Merged.jpg]

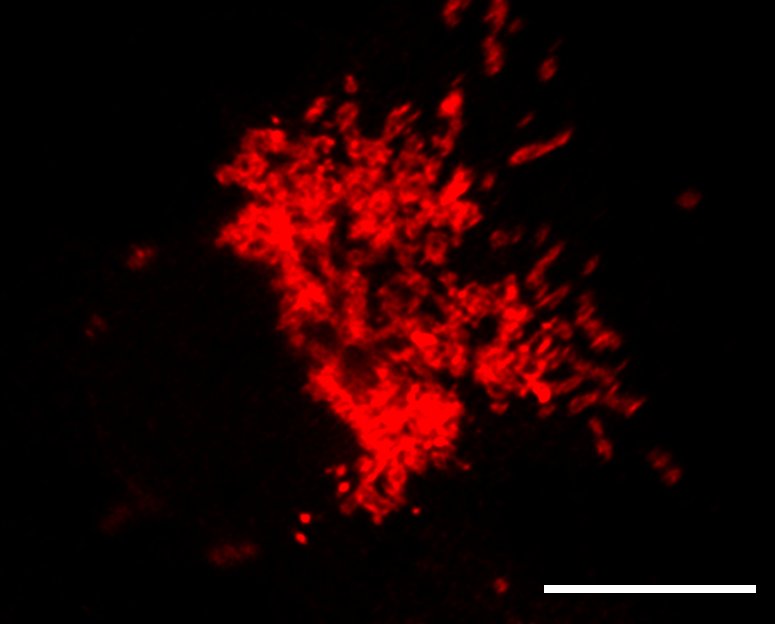

Supplement: Supplementary file 9 — Source Data Fig. 4 [file 44319_2024_58_MOESM9_ESM.zip › Fig 4 Source data/Fig 4C/Fig 4C shTFEBFLAG - TOMM20.jpg]

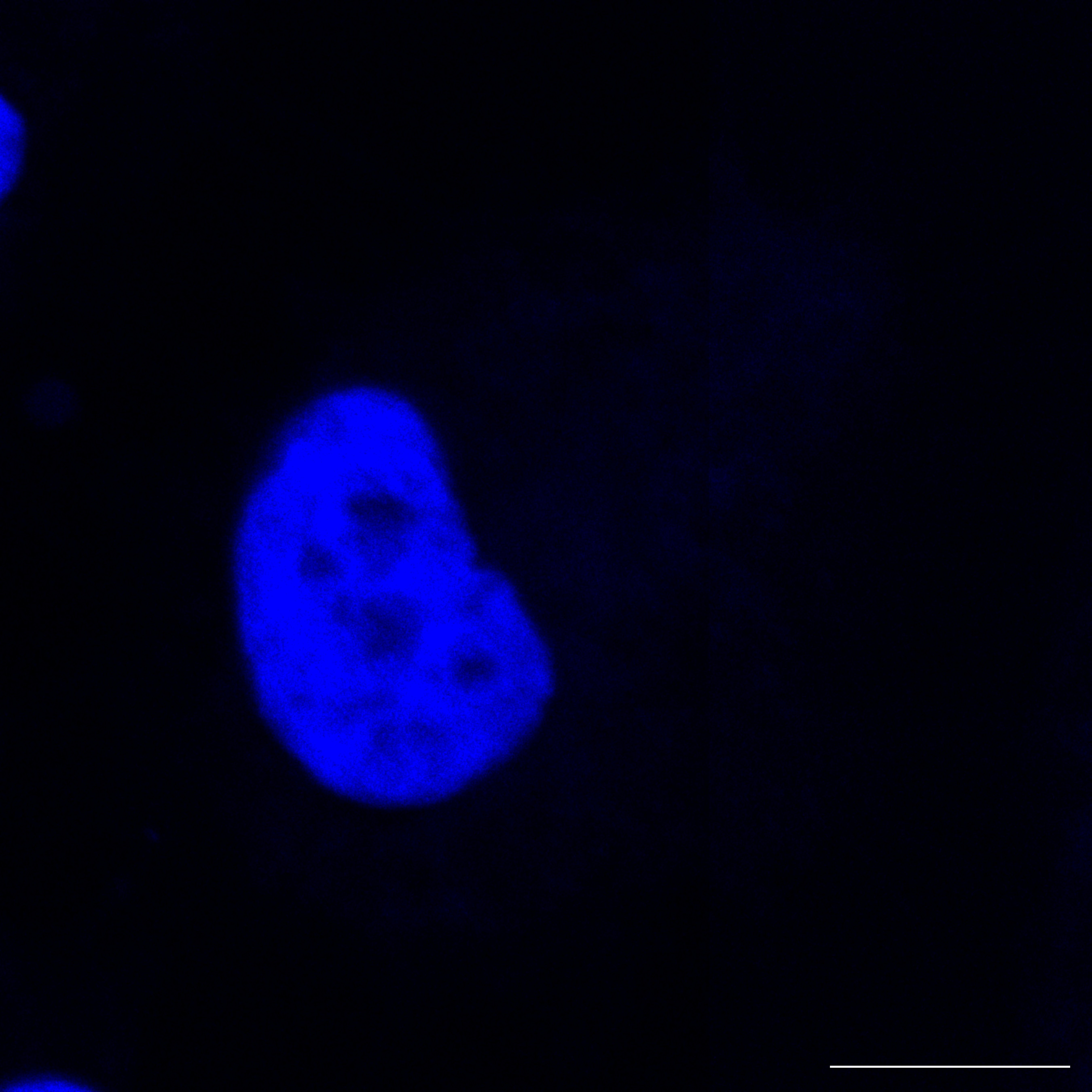

Supplement: Supplementary file 9 — Source Data Fig. 4 [file 44319_2024_58_MOESM9_ESM.zip › Fig 4 Source data/Fig 4C/Fig 4C shTFEBMLSFLAG - DAPI.jpg]

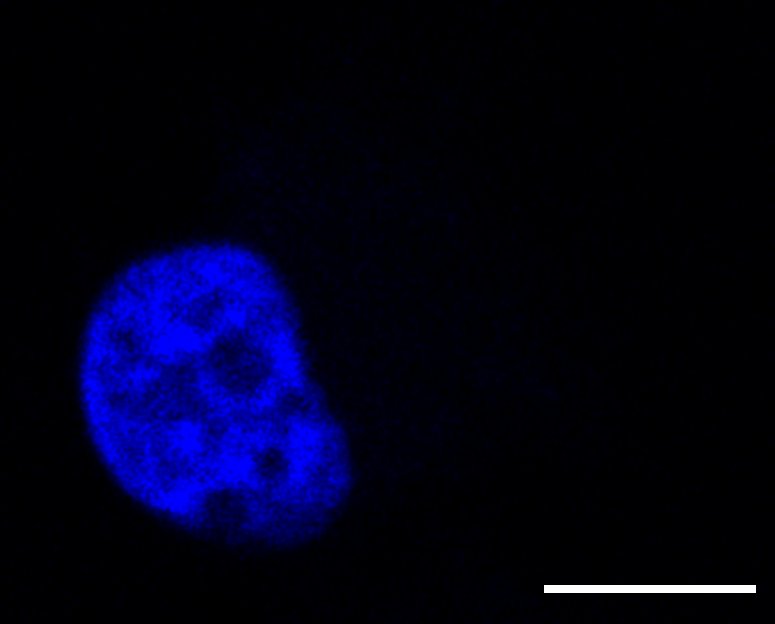

Supplement: Supplementary file 9 — Source Data Fig. 4 [file 44319_2024_58_MOESM9_ESM.zip › Fig 4 Source data/Fig 4C/Fig 4C shTFEBFLAG - DAPI.jpg]

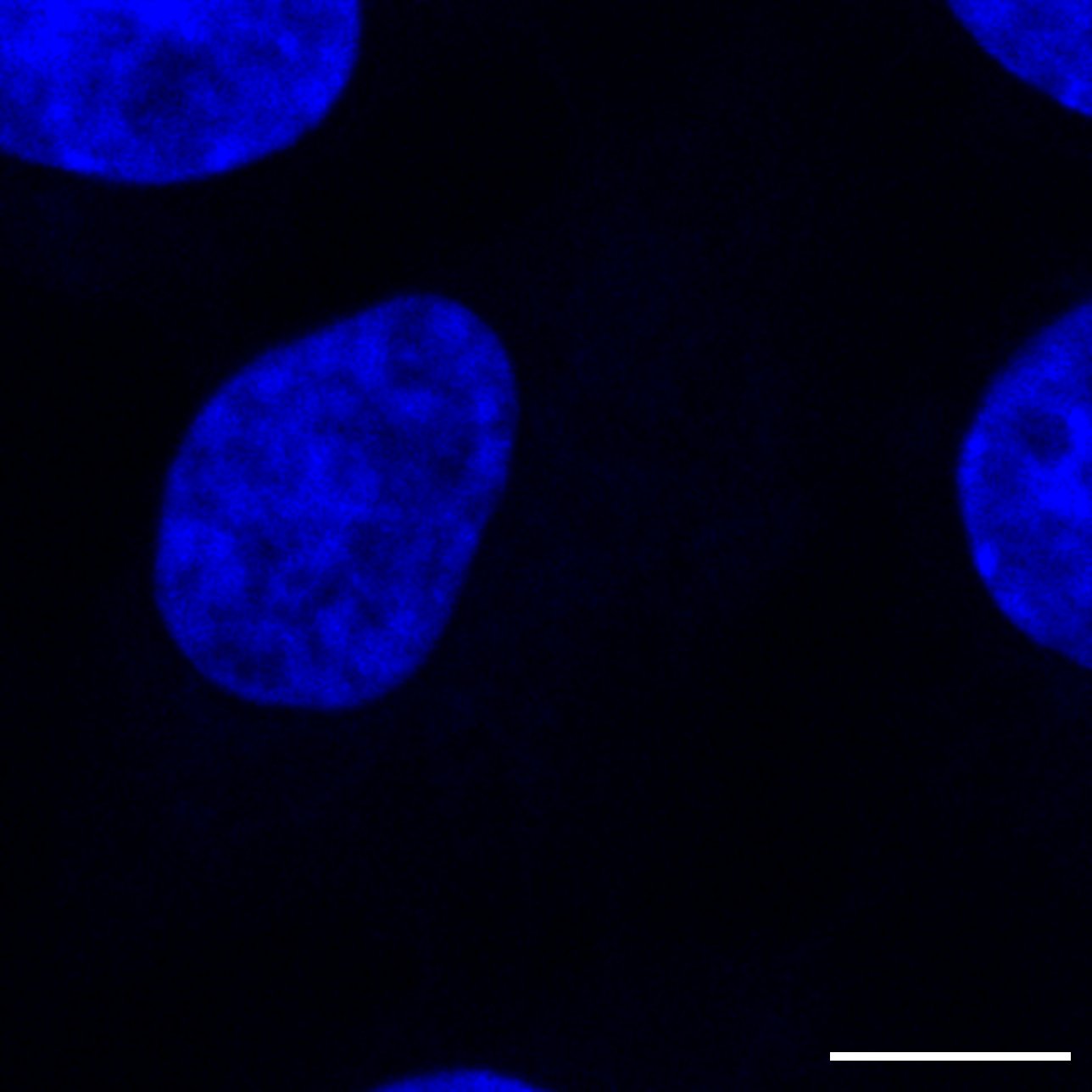

Supplement: Supplementary file 9 — Source Data Fig. 4 [file 44319_2024_58_MOESM9_ESM.zip › Fig 4 Source data/Fig 4C/Fig 4C WTFLAG - DAPI.jpg]

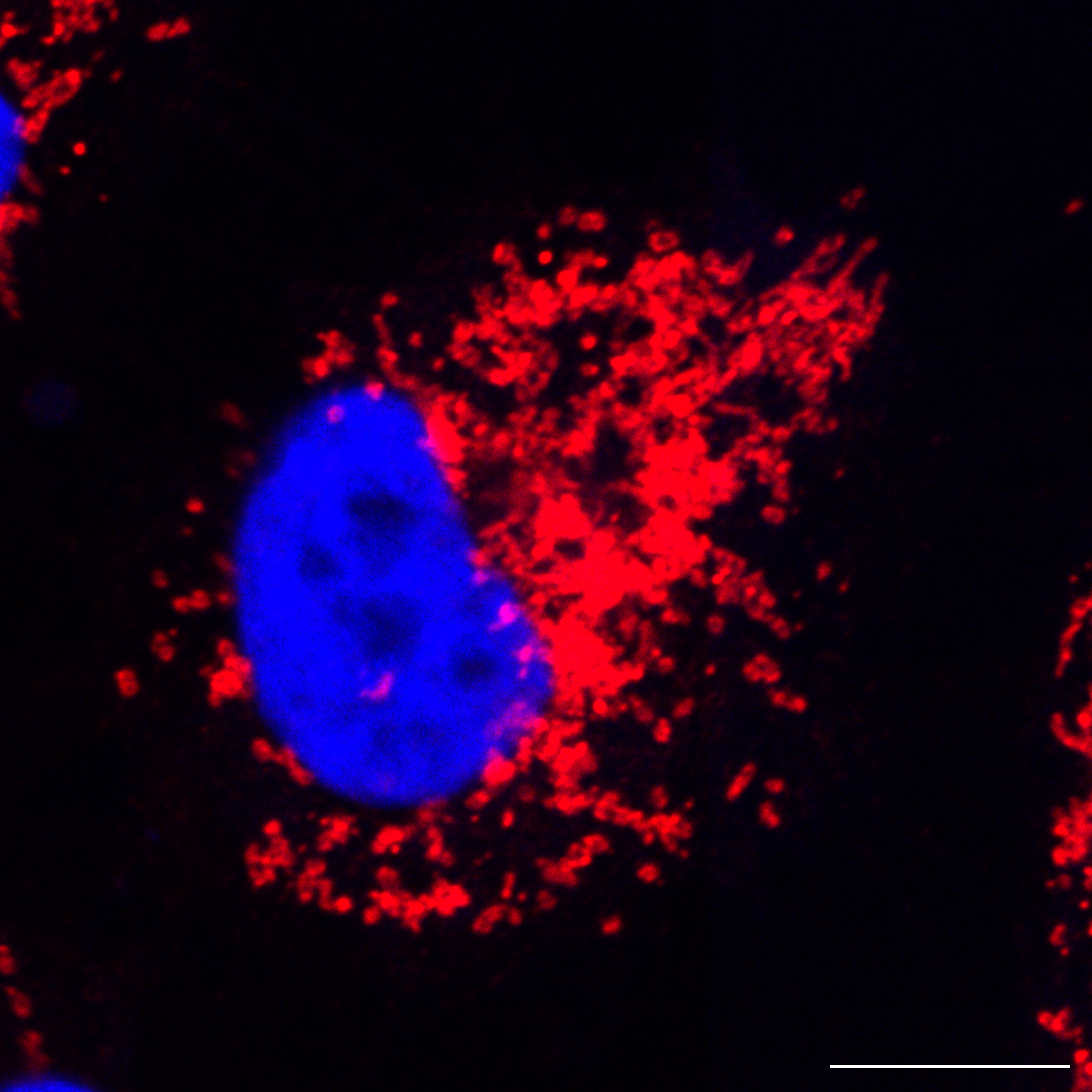

Supplement: Supplementary file 9 — Source Data Fig. 4 [file 44319_2024_58_MOESM9_ESM.zip › Fig 4 Source data/Fig 4C/Fig 4C MLSTFEBFLAG - Merged.jpg]

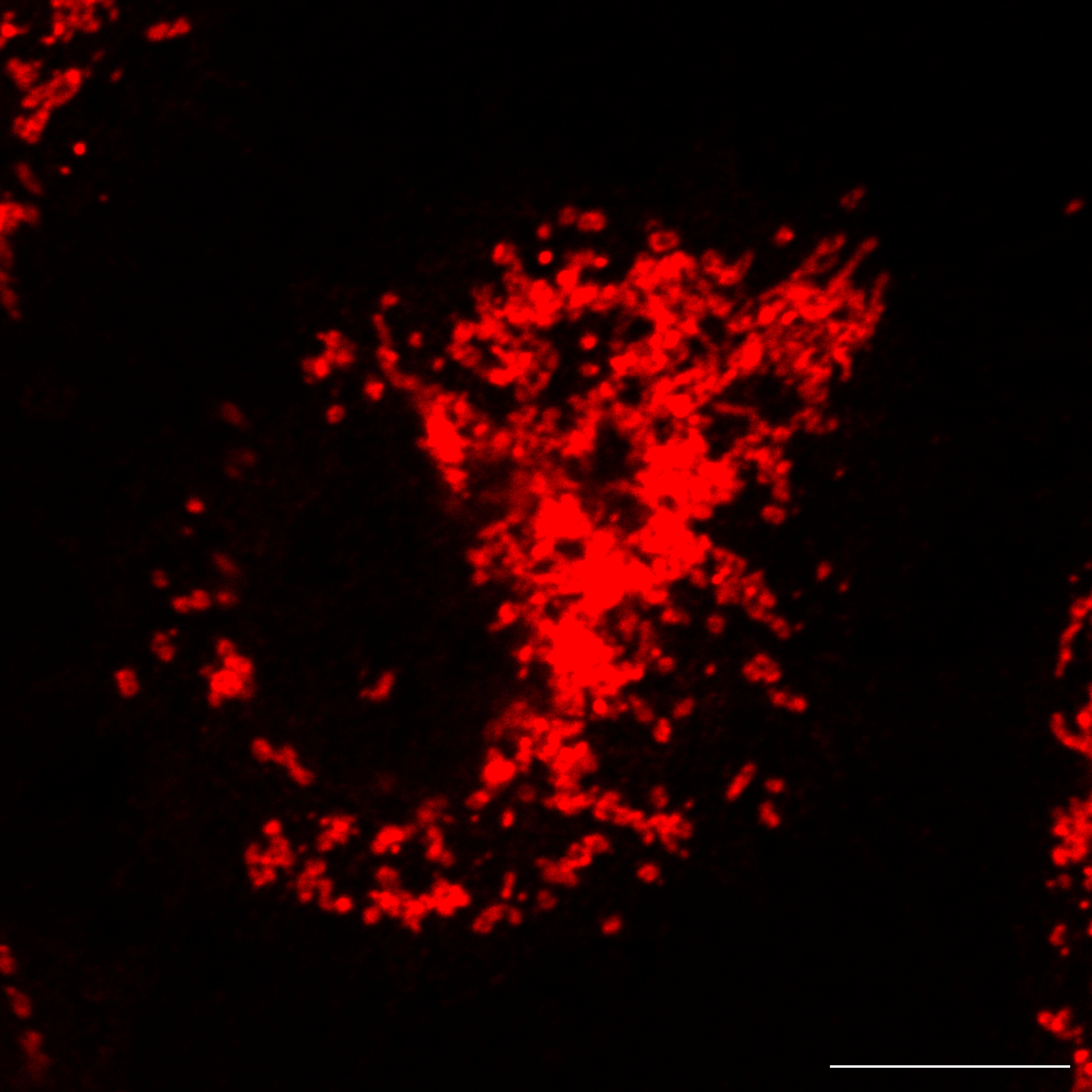

Supplement: Supplementary file 9 — Source Data Fig. 4 [file 44319_2024_58_MOESM9_ESM.zip › Fig 4 Source data/Fig 4C/Fig 4C MLSTFEBFLAG - TOMM20.jpg]

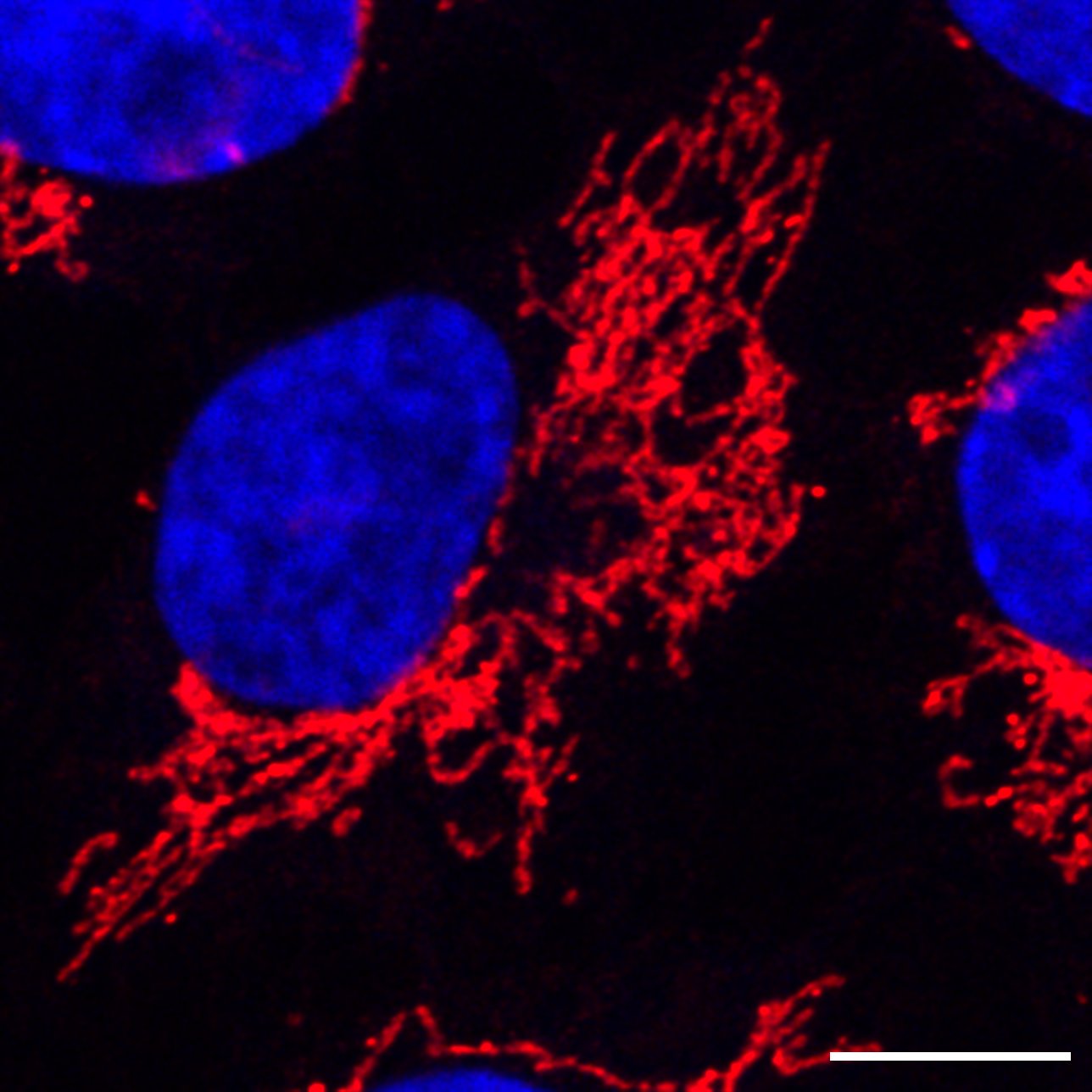

Supplement: Supplementary file 9 — Source Data Fig. 4 [file 44319_2024_58_MOESM9_ESM.zip › Fig 4 Source data/Fig 4C/Fig 4C WTFLAG - Merged.jpg]

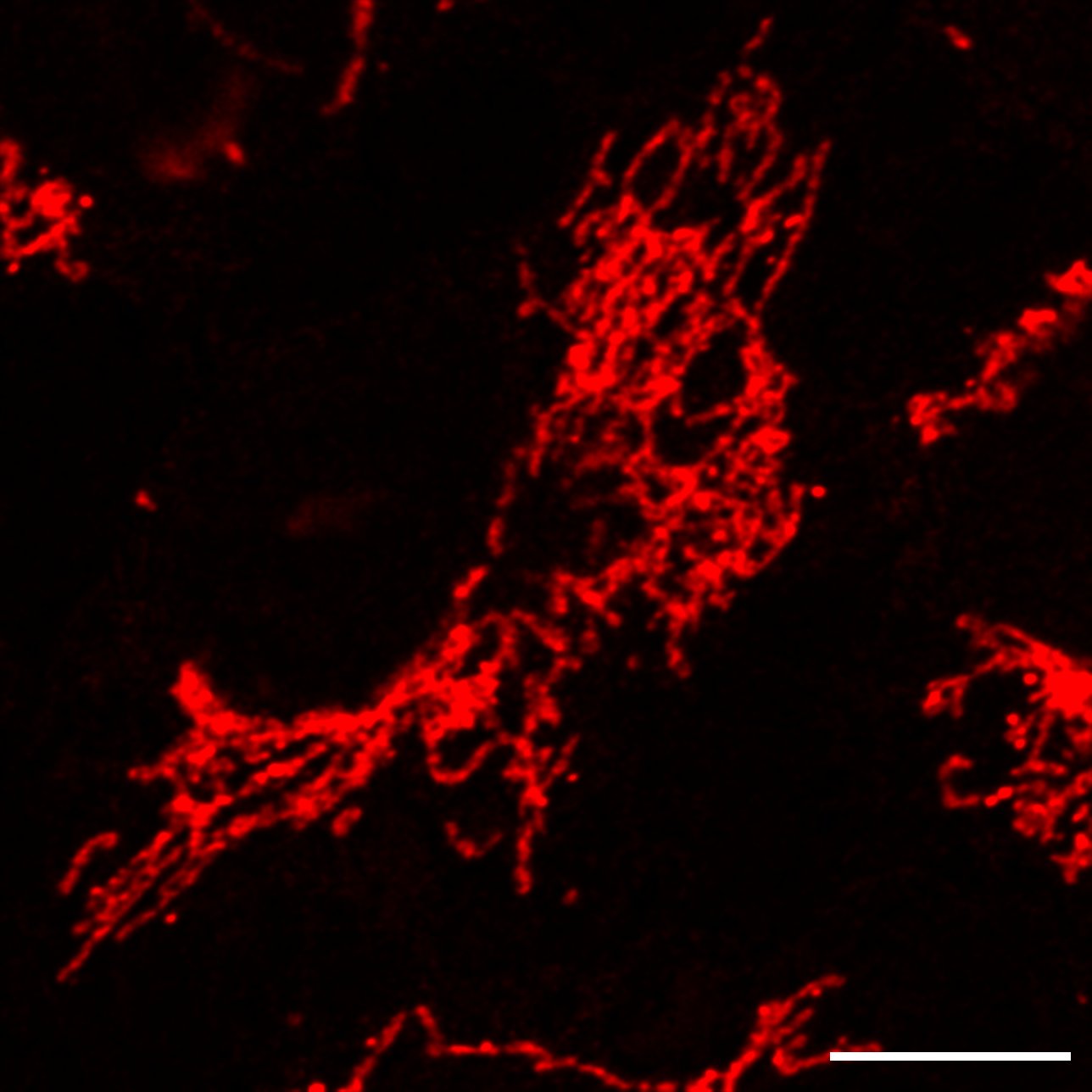

Supplement: Supplementary file 9 — Source Data Fig. 4 [file 44319_2024_58_MOESM9_ESM.zip › Fig 4 Source data/Fig 4C/Fig 4C WTFLAG - TOMM20.jpg]

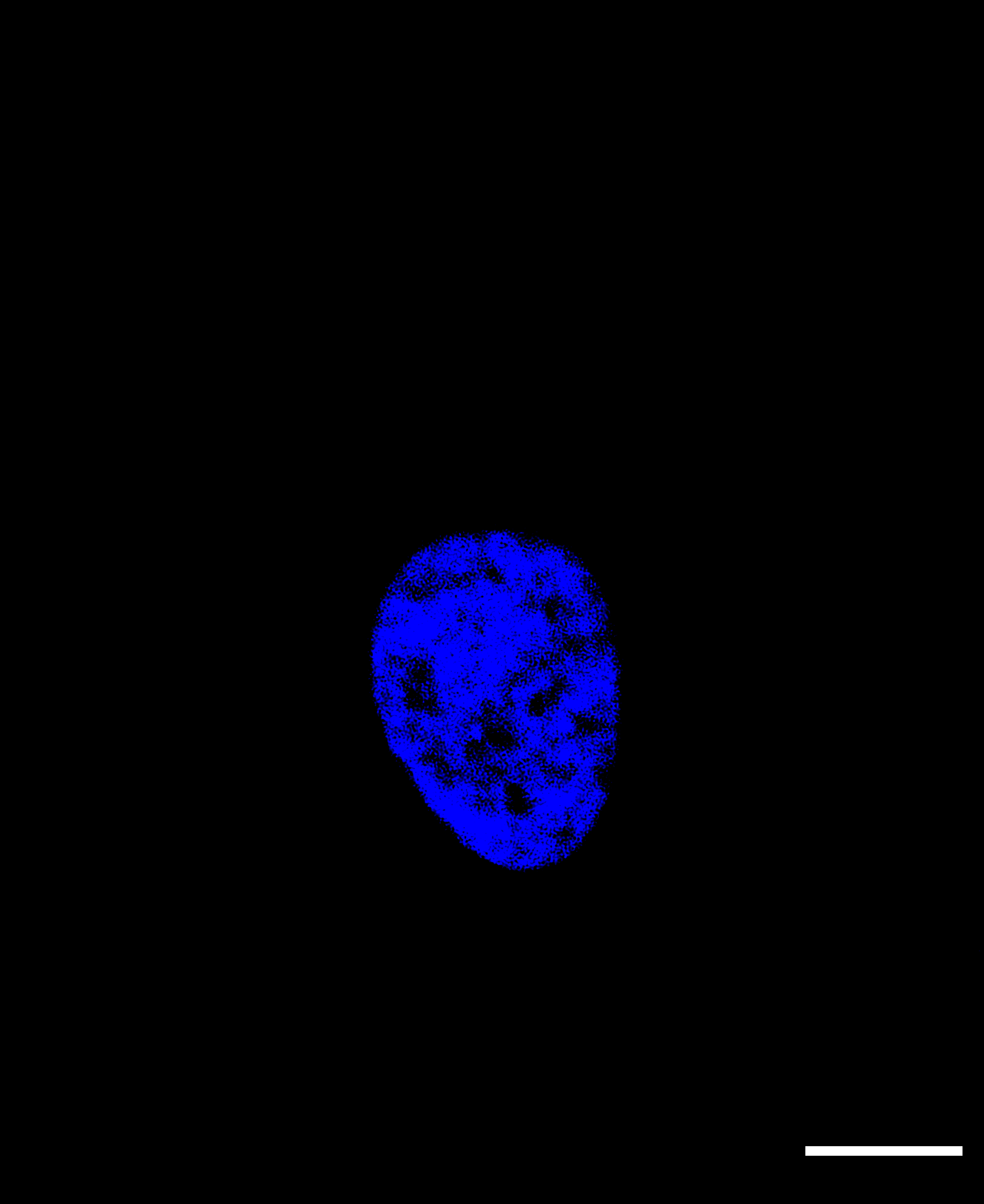

Supplement: Supplementary file 9 — Source Data Fig. 4 [file 44319_2024_58_MOESM9_ESM.zip › Fig 4 Source data/Fig 4A/Fig 4A shTFEB - DAPI.tif]

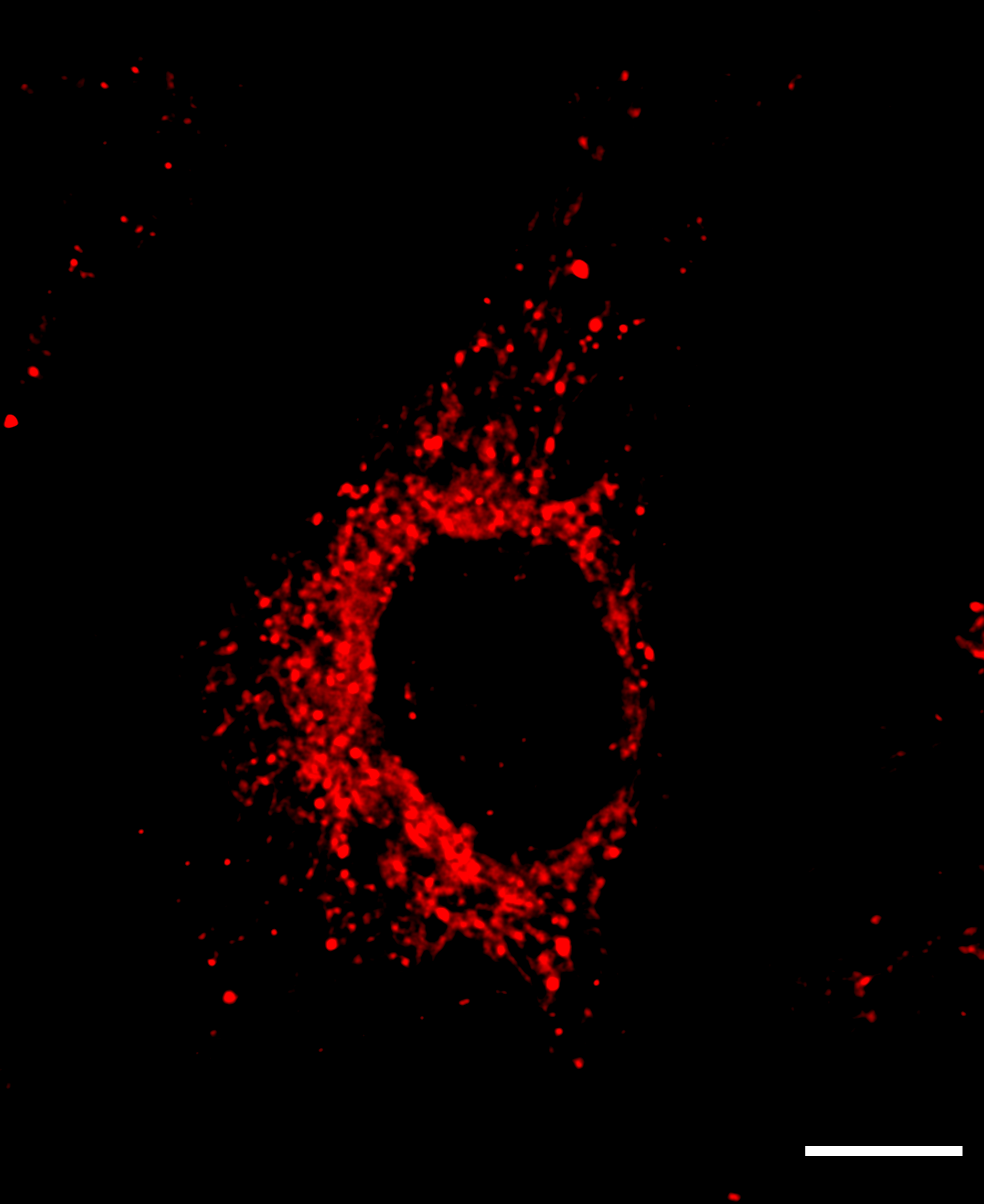

Supplement: Supplementary file 9 — Source Data Fig. 4 [file 44319_2024_58_MOESM9_ESM.zip › Fig 4 Source data/Fig 4A/Fig 4A shTFEB - TOMM20.tif]

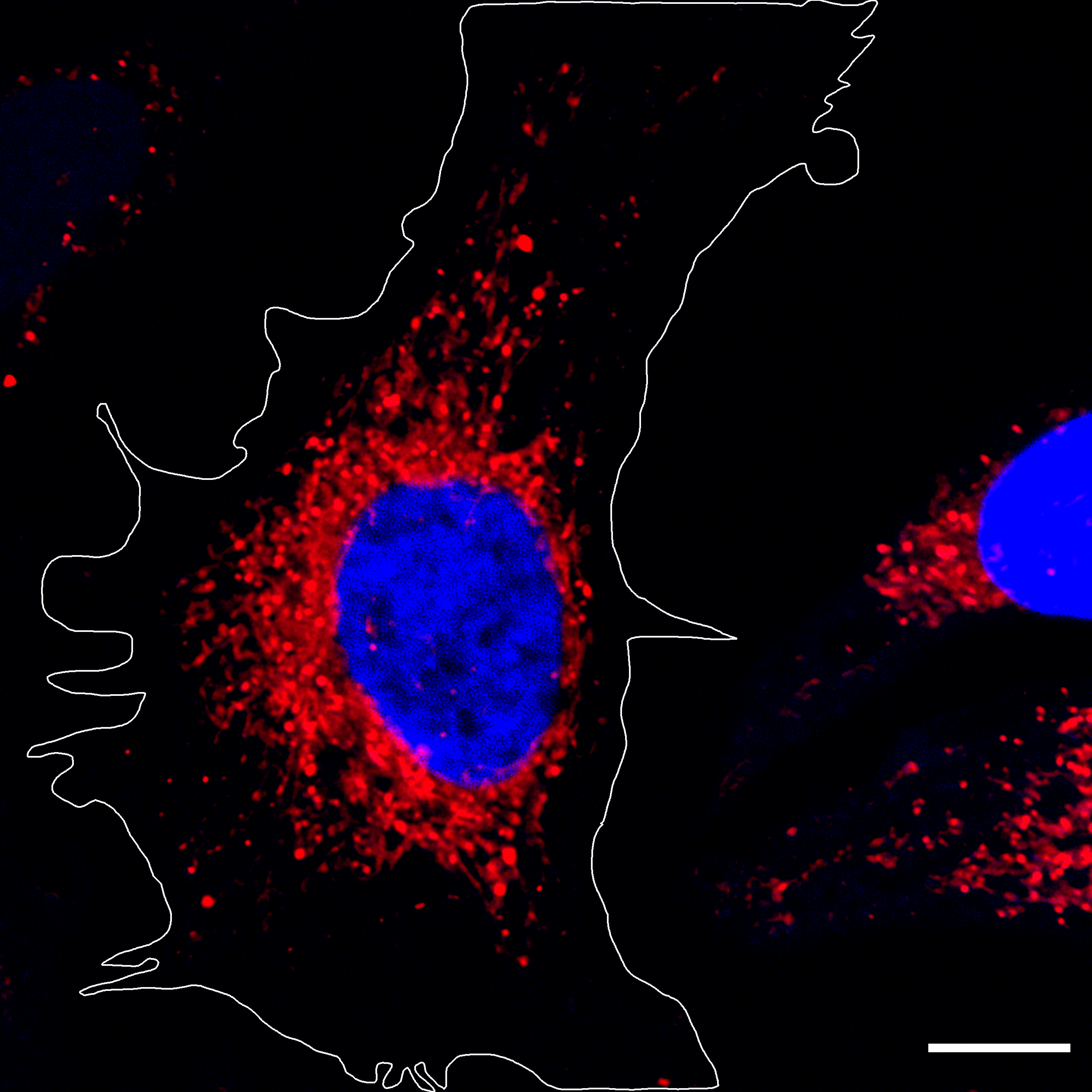

Supplement: Supplementary file 9 — Source Data Fig. 4 [file 44319_2024_58_MOESM9_ESM.zip › Fig 4 Source data/Fig 4A/Fig 4A shTFEB - Merged.tif]

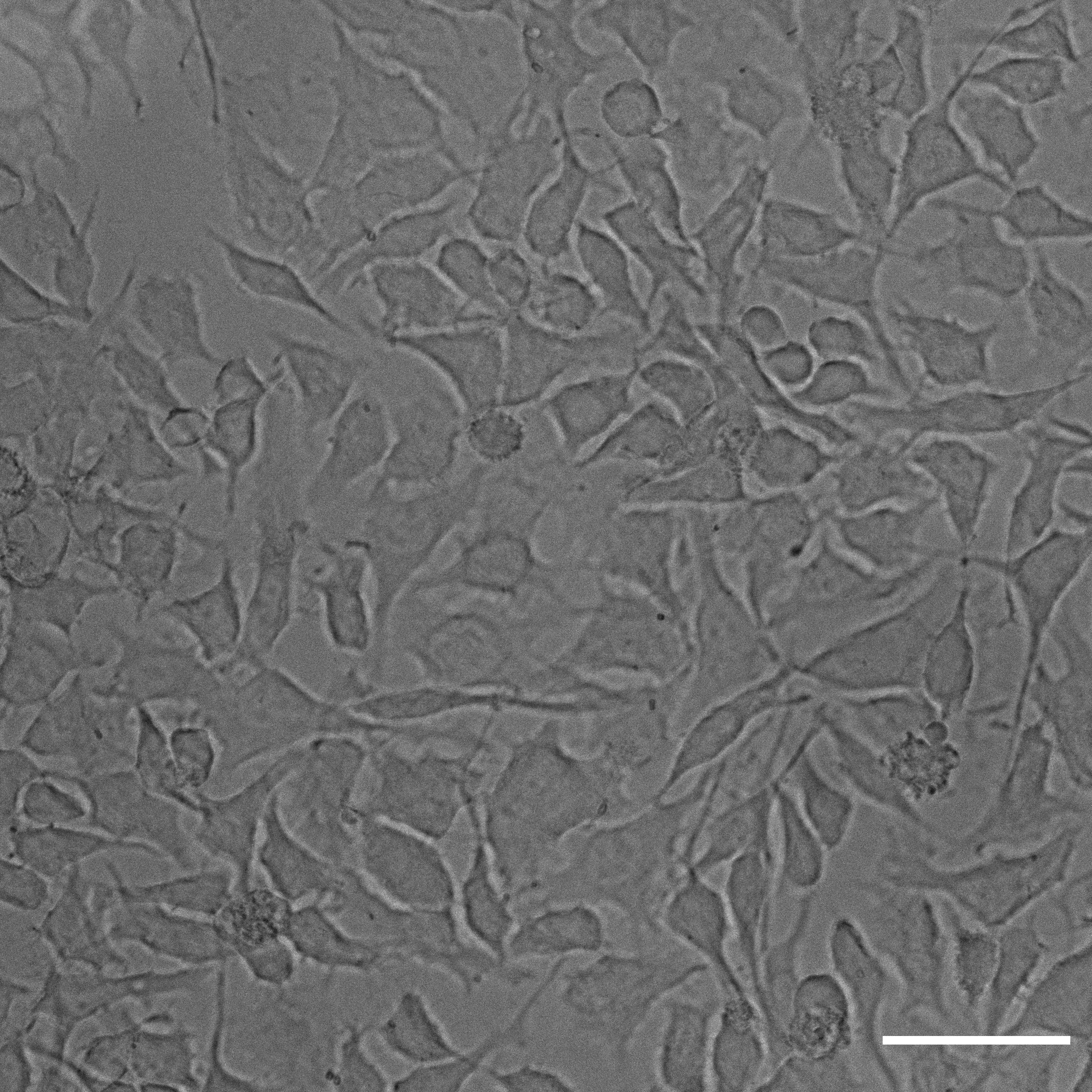

Supplement: Supplementary file 9 — Source Data Fig. 4 [file 44319_2024_58_MOESM9_ESM.zip › Fig 4 Source data/Fig 4A/Fig 4A shTFEB - Phase Contrast.tif]

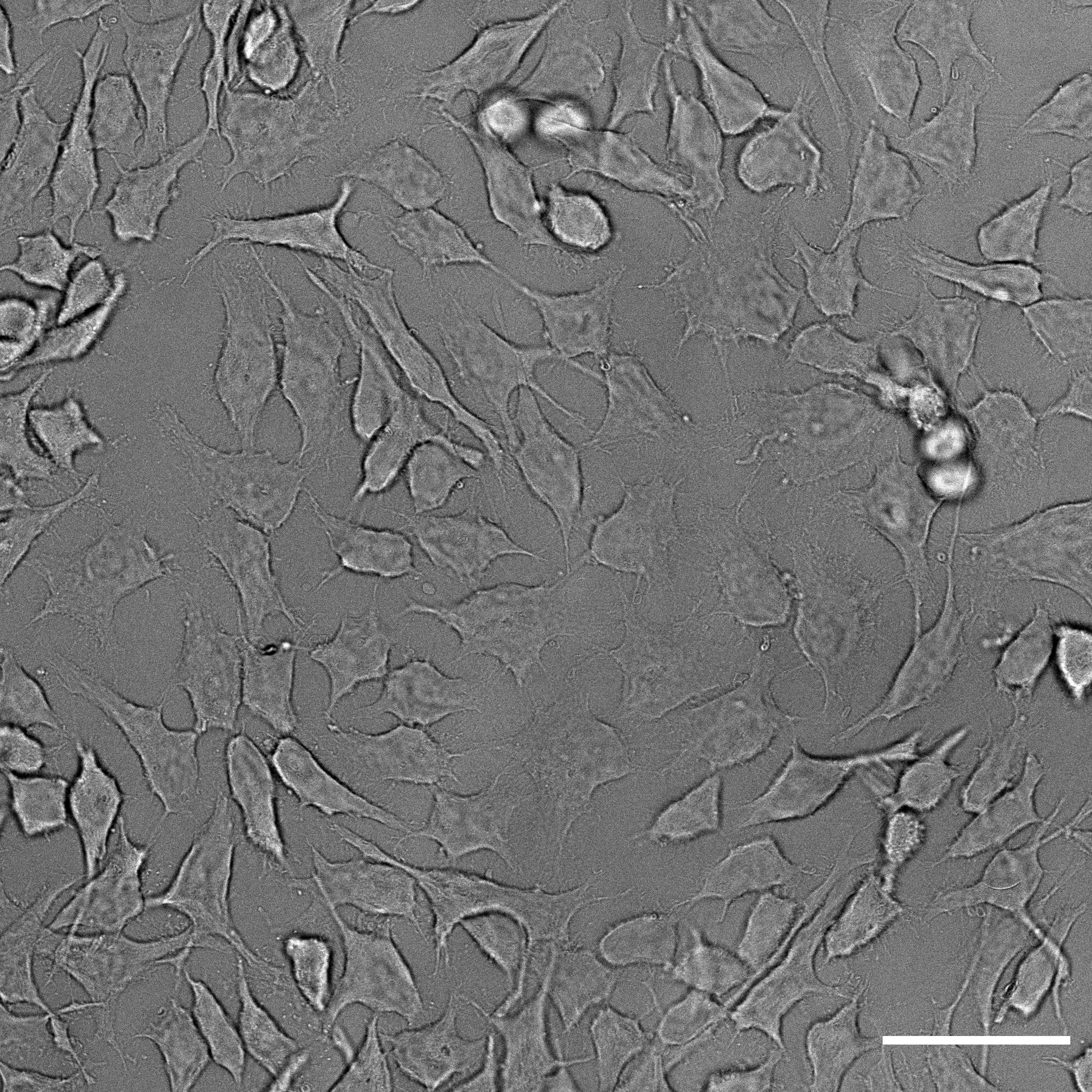

Supplement: Supplementary file 9 — Source Data Fig. 4 [file 44319_2024_58_MOESM9_ESM.zip › Fig 4 Source data/Fig 4A/Fig 4A shCTRL - Phase Contrast.tif]

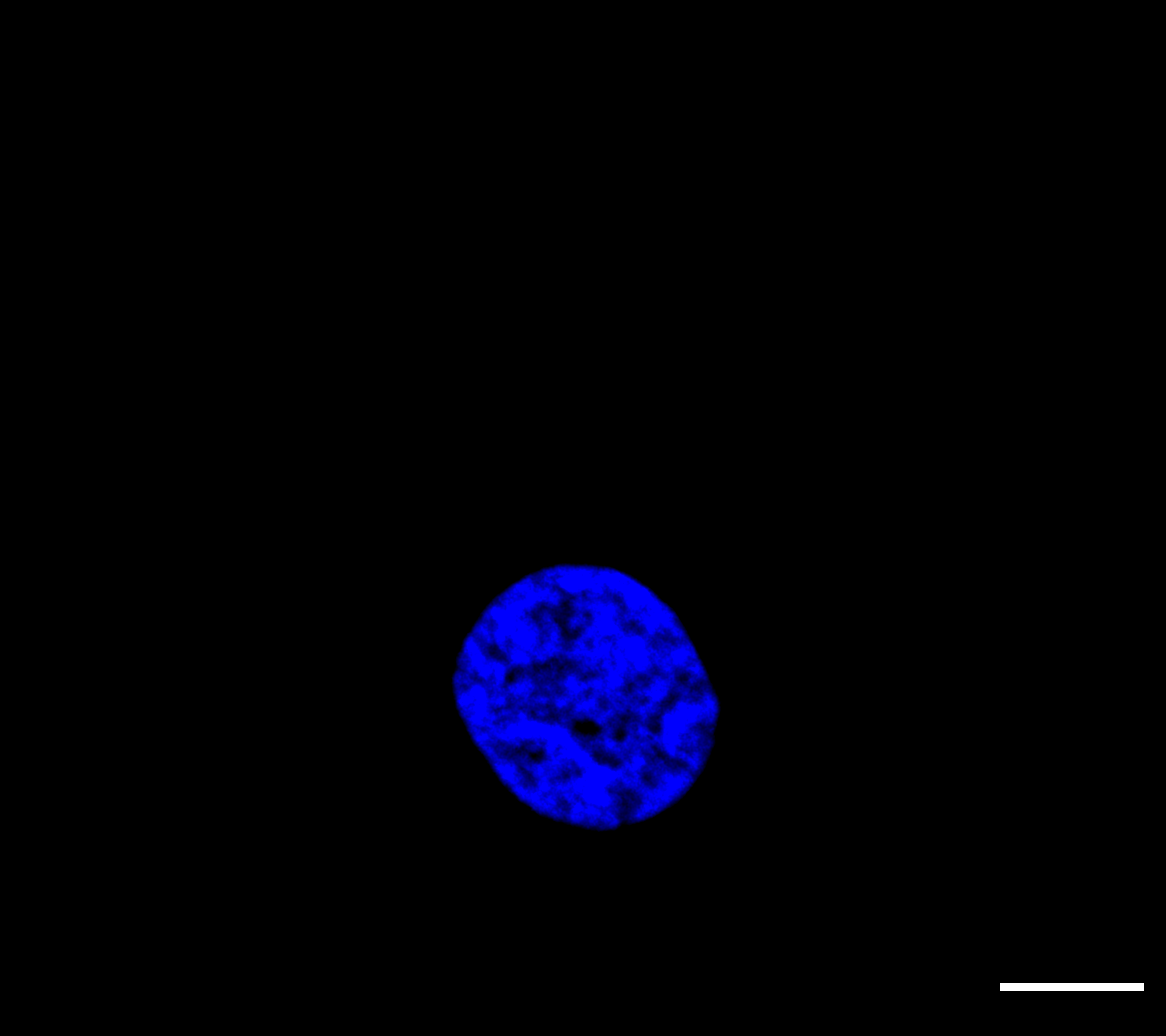

Supplement: Supplementary file 9 — Source Data Fig. 4 [file 44319_2024_58_MOESM9_ESM.zip › Fig 4 Source data/Fig 4A/Fig 4A shCTRL - DAPI.tif]

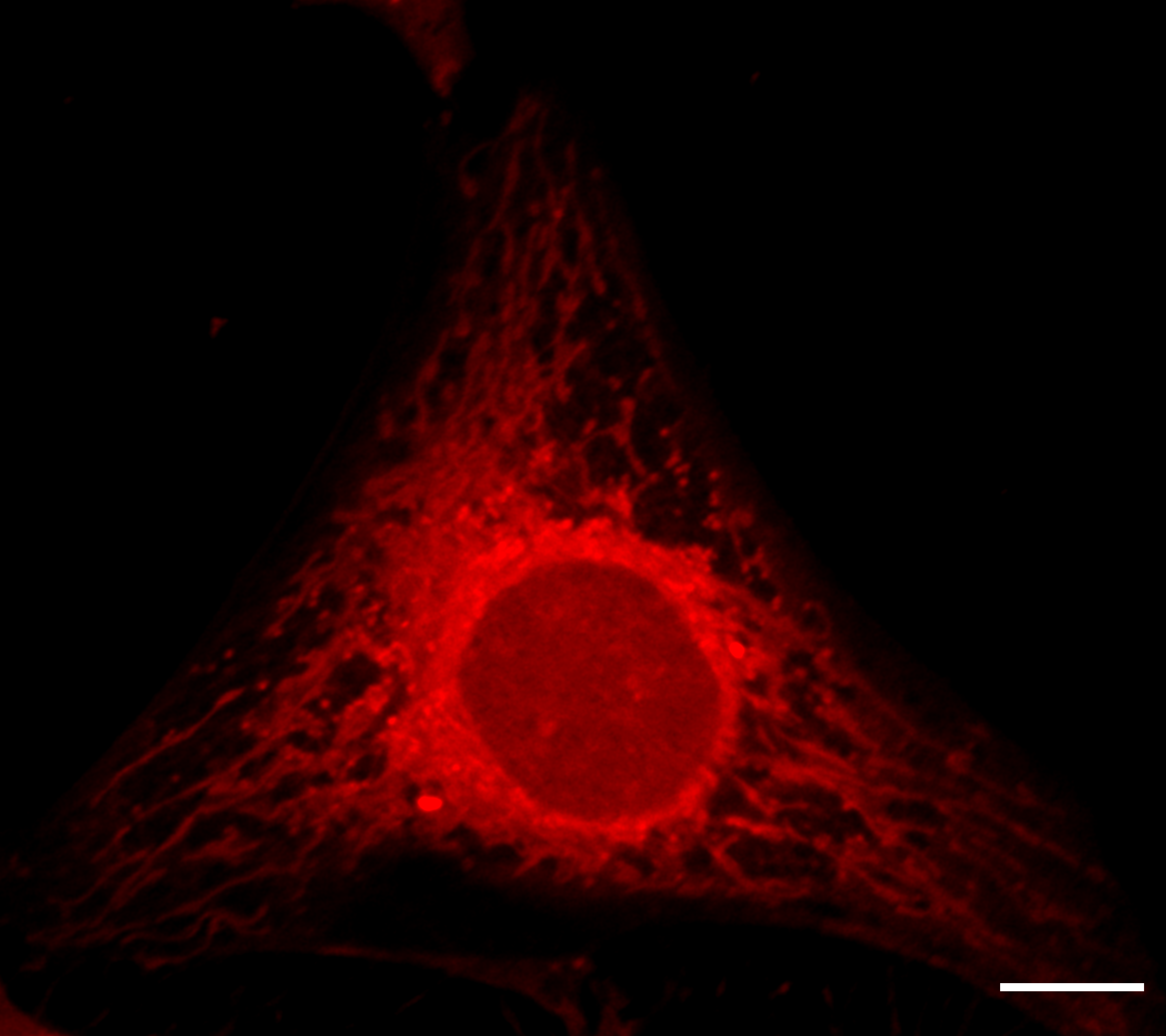

Supplement: Supplementary file 9 — Source Data Fig. 4 [file 44319_2024_58_MOESM9_ESM.zip › Fig 4 Source data/Fig 4A/Fig 4A shCTRL - TOMM20.tif]

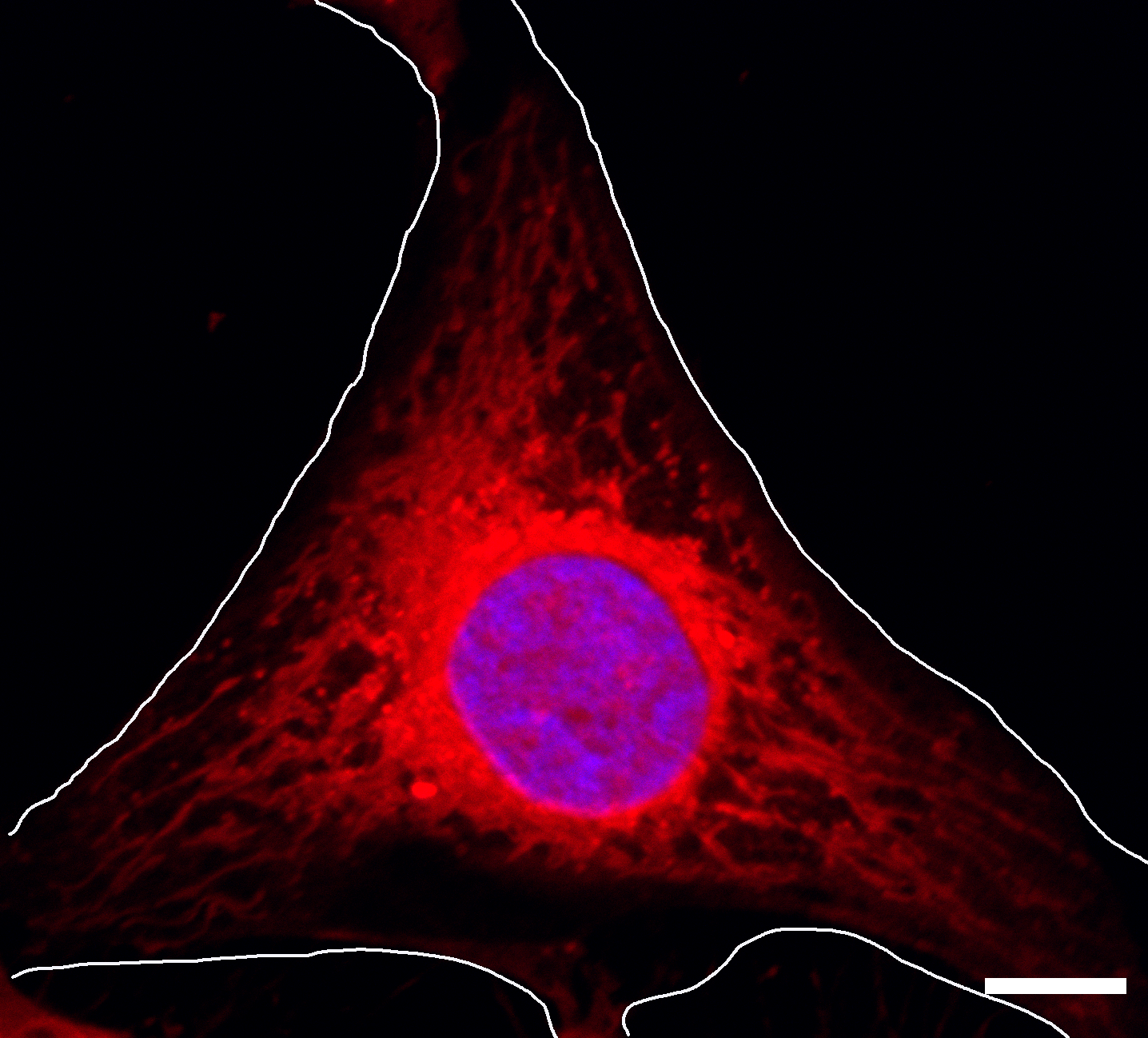

Supplement: Supplementary file 9 — Source Data Fig. 4 [file 44319_2024_58_MOESM9_ESM.zip › Fig 4 Source data/Fig 4A/Fig 4A shCTRL - Merged.tif]

**Fig. 5G**

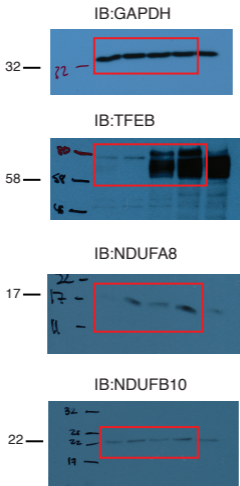

Supplement: Supplementary file 10 — Source Data Fig. 5 [file 44319_2024_58_MOESM10_ESM.zip › Fig 5 Source data/Fig 5G/Fig 5G Unprocessed blots.pdf]

**Fig. 5E**

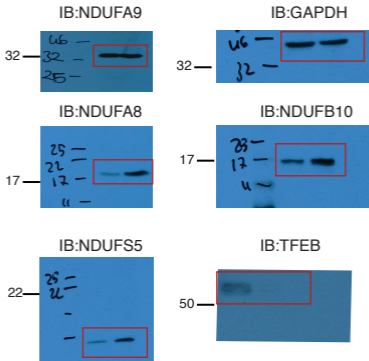

Supplement: Supplementary file 10 — Source Data Fig. 5 [file 44319_2024_58_MOESM10_ESM.zip › Fig 5 Source data/Fig 5E/Fig 5E unprocessed blots.pdf]

**Fig. 5B**

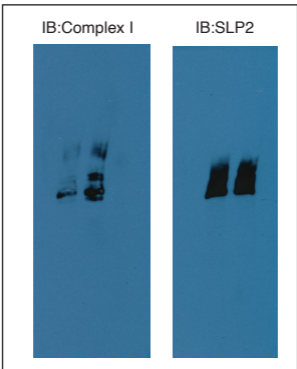

Supplement: Supplementary file 10 — Source Data Fig. 5 [file 44319_2024_58_MOESM10_ESM.zip › Fig 5 Source data/Fig 5B/Fig 5B unprocessed blots.pdf]

**Fig. 5C**

Blue native:Complex I

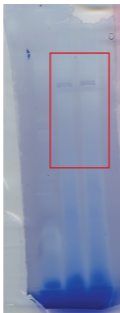

Supplement: Supplementary file 10 — Source Data Fig. 5 [file 44319_2024_58_MOESM10_ESM.zip › Fig 5 Source data/Fig 5C/Fig 5C unprocessed blots.pdf]

**Fig. 6I**

IB:LONP1-HA

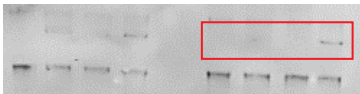

IB:LONP1-HA

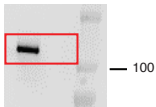

IP:TFEB-FLAG

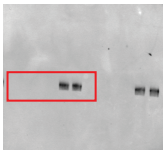

Supplement: Supplementary file 11 — Source Data Fig. 6 [file 44319_2024_58_MOESM11_ESM.zip › Fig 6 Source data/Fig 6I/Fig 6I unprocessed blots.pdf]

**Fig. 6G**

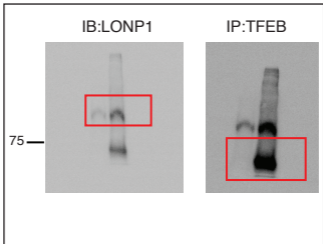

Supplement: Supplementary file 11 — Source Data Fig. 6 [file 44319_2024_58_MOESM11_ESM.zip › Fig 6 Source data/Fig 6G/Fig 6G unprocessed blots.pdf]

**Fig. 6A**

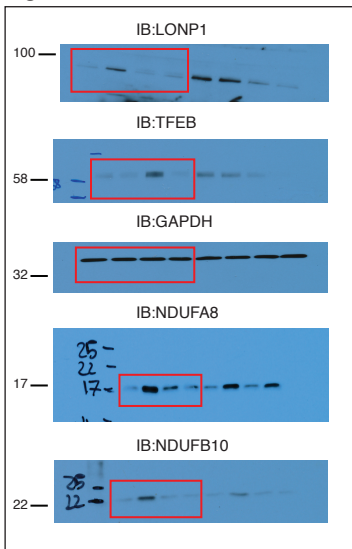

Supplement: Supplementary file 11 — Source Data Fig. 6 [file 44319_2024_58_MOESM11_ESM.zip › Fig 6 Source data/Fig 6A/Fig 6A Unprocessed blots.pdf]

**Fig. 6H**

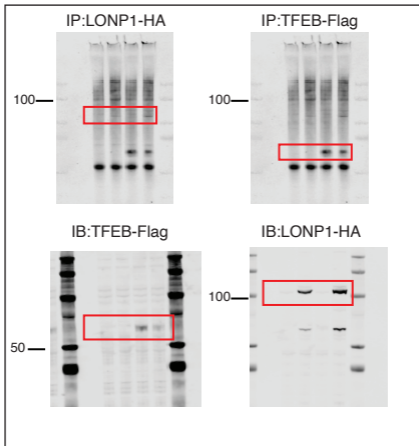

Supplement: Supplementary file 11 — Source Data Fig. 6 [file 44319_2024_58_MOESM11_ESM.zip › Fig 6 Source data/Fig 6H/Fig 6H unprocessed blots.pdf]

**Fig. 6C**

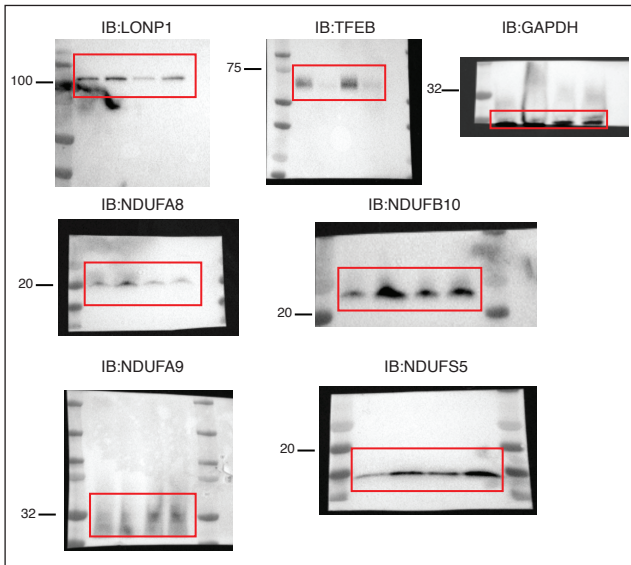

Supplement: Supplementary file 11 — Source Data Fig. 6 [file 44319_2024_58_MOESM11_ESM.zip › Fig 6 Source data/Fig 6C/Fig 6C unprocessed blots.pdf]

**Fig. 7A**

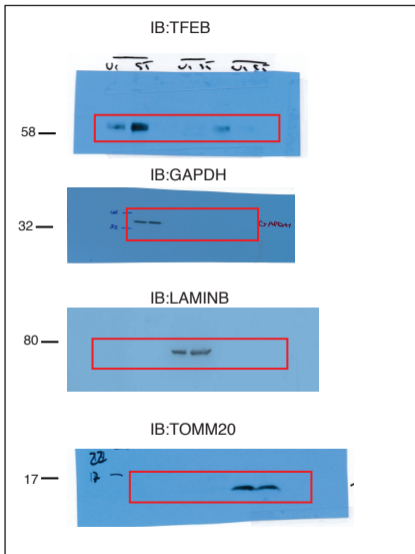

Supplement: Supplementary file 12 — Source Data Fig. 7 [file 44319_2024_58_MOESM12_ESM.zip › Fig 7 Source data/Fig 7A/Fig 7A Unprocessed blots.pdf]

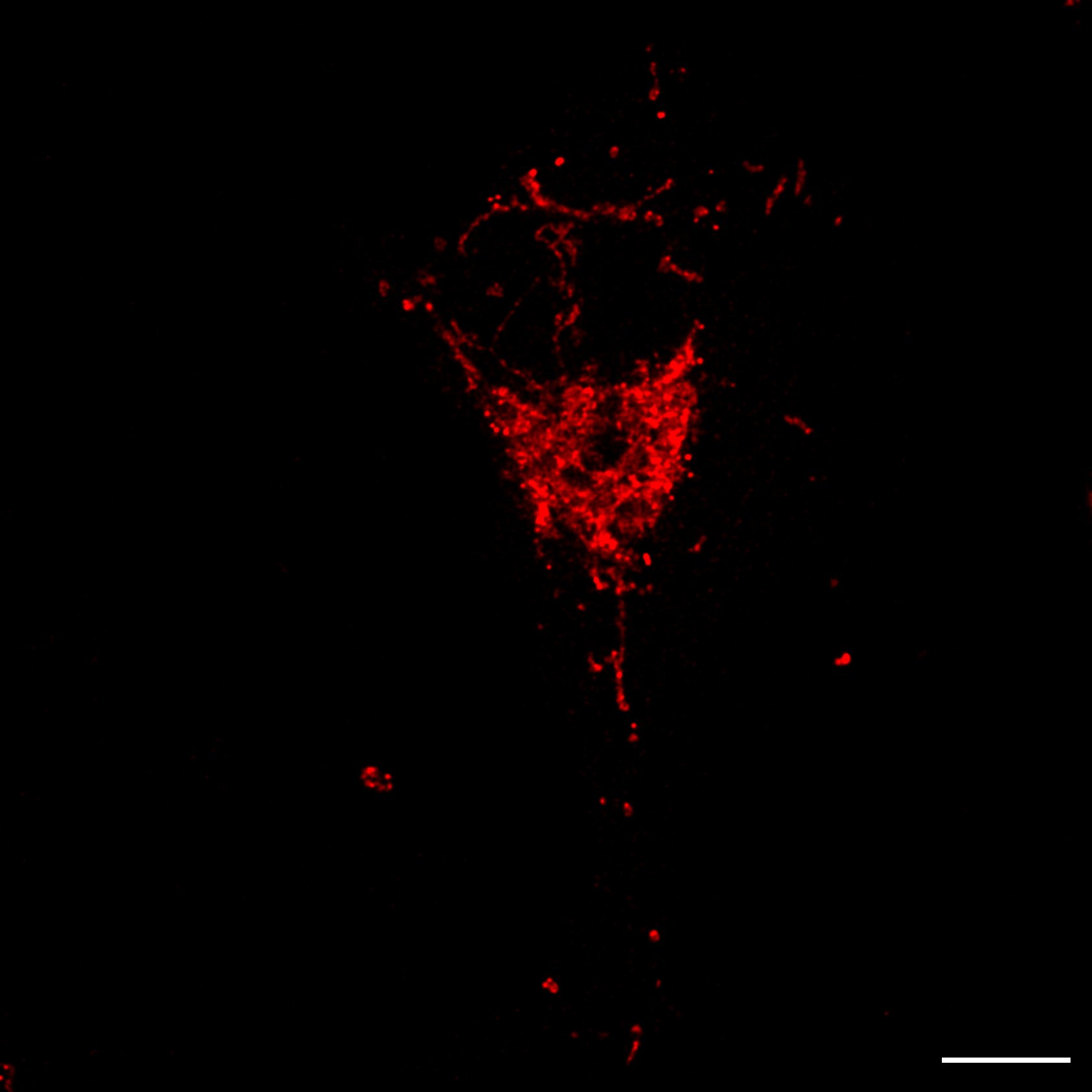

Supplement: Supplementary file 13 — Appendix Figures Source Data [file 44319_2024_58_MOESM13_ESM.zip › Appendix source data/Fig S3 Source data/Fig S3C/Fig S3C MLS-TFEB FLAG - Mitotracker.jpg]

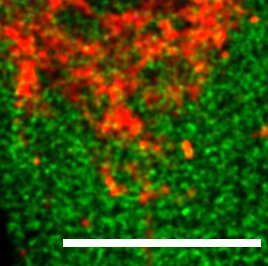

Supplement: Supplementary file 13 — Appendix Figures Source Data [file 44319_2024_58_MOESM13_ESM.zip › Appendix source data/Fig S3 Source data/Fig S3C/Fig S3C MLS-TFEB FLAG - Inset Merged.jpg]

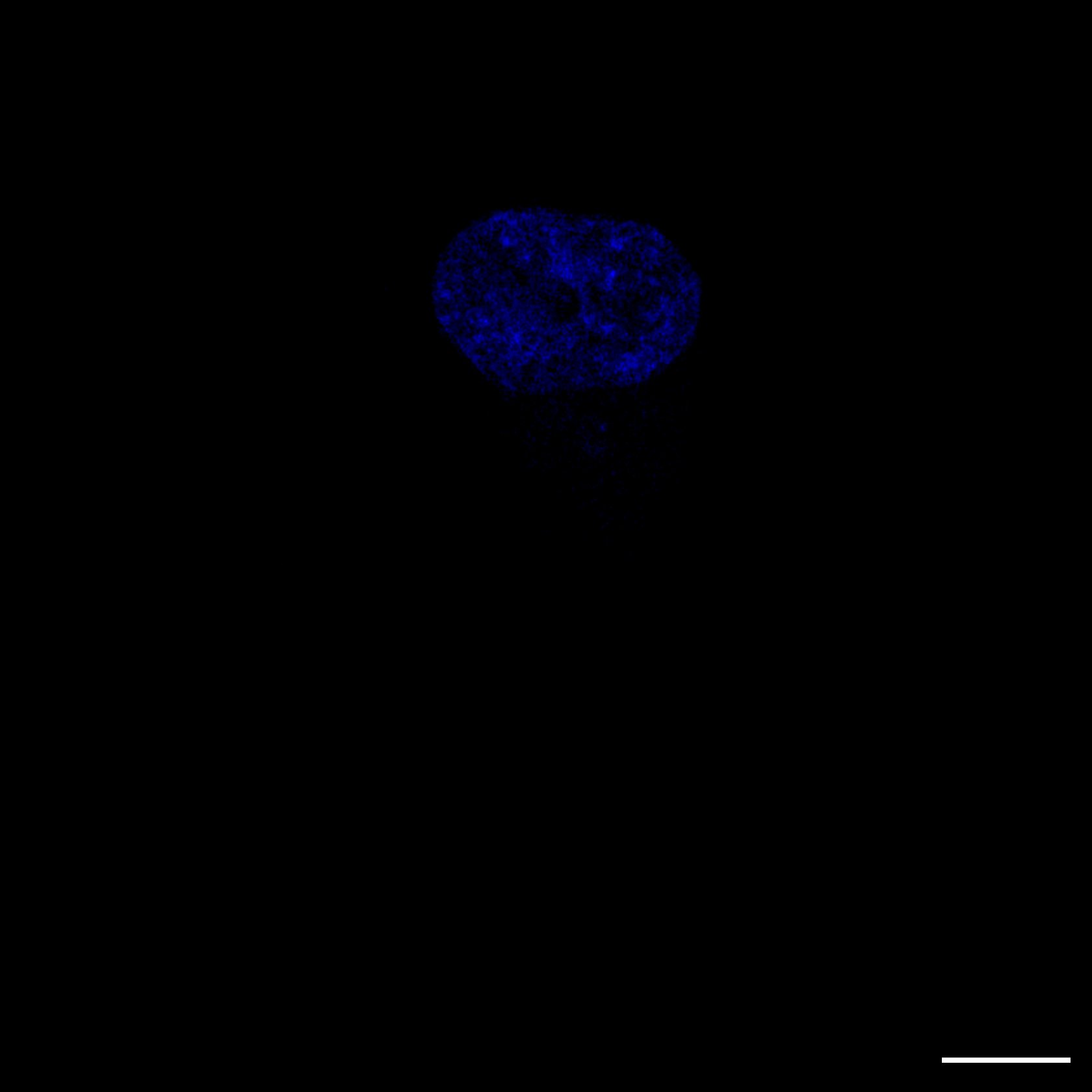

Supplement: Supplementary file 13 — Appendix Figures Source Data [file 44319_2024_58_MOESM13_ESM.zip › Appendix source data/Fig S3 Source data/Fig S3C/Fig S3C MLS-TFEB FLAG - DAPI.jpg]

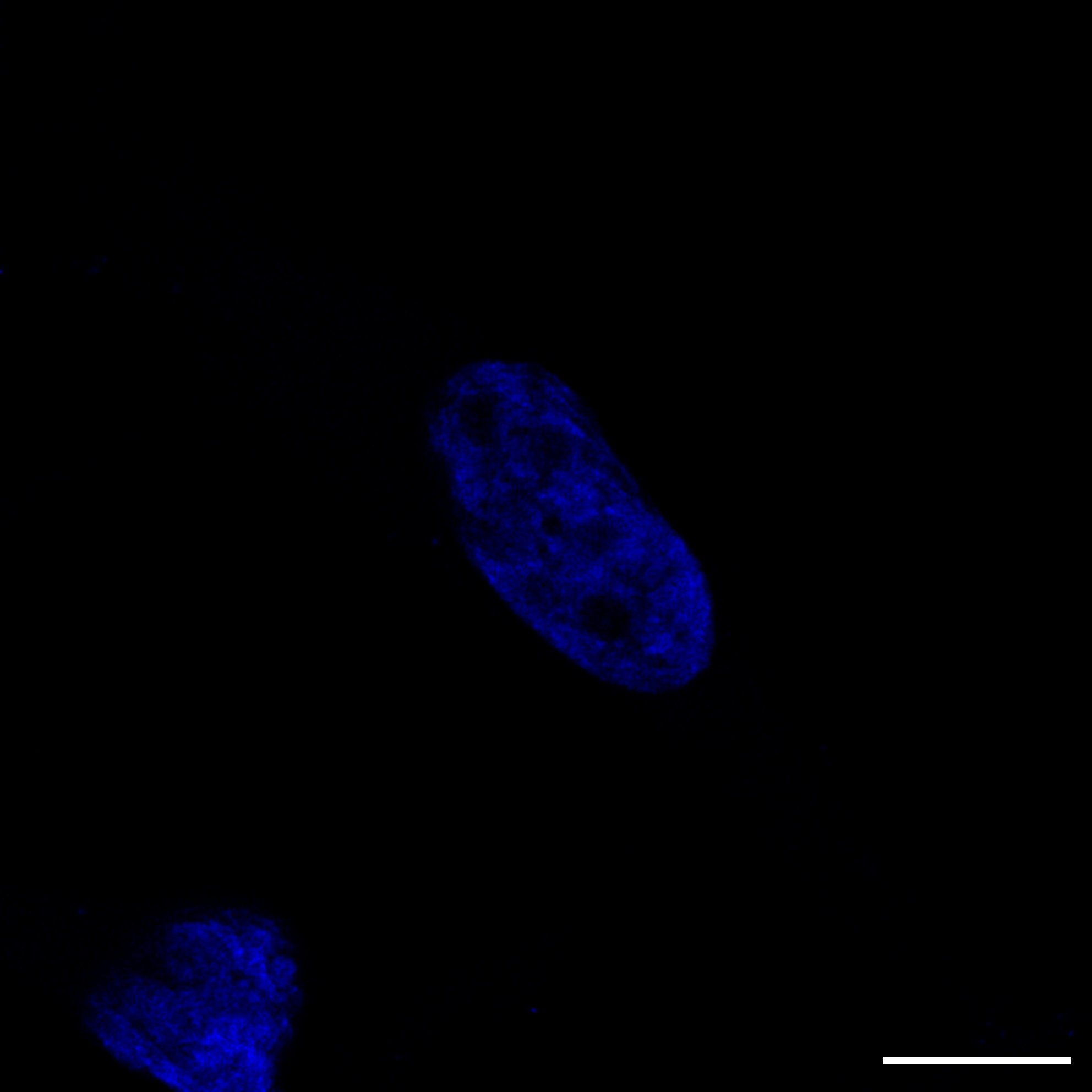

Supplement: Supplementary file 13 — Appendix Figures Source Data [file 44319_2024_58_MOESM13_ESM.zip › Appendix source data/Fig S3 Source data/Fig S3C/Fig S3C WT-TFEB FLAG - DAPI.jpg]

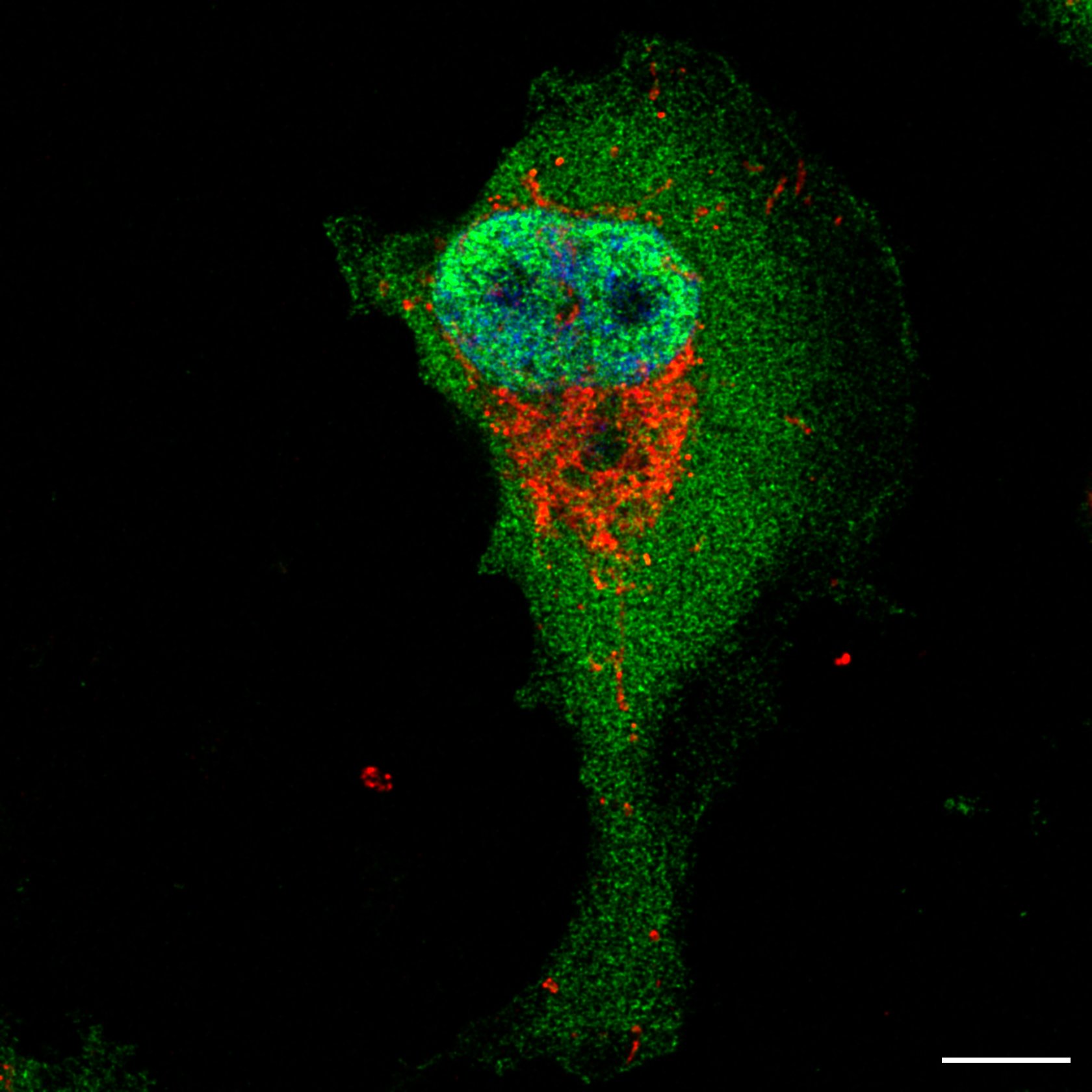

Supplement: Supplementary file 13 — Appendix Figures Source Data [file 44319_2024_58_MOESM13_ESM.zip › Appendix source data/Fig S3 Source data/Fig S3C/Fig S3C MLS-TFEB FLAG - Merged.jpg]

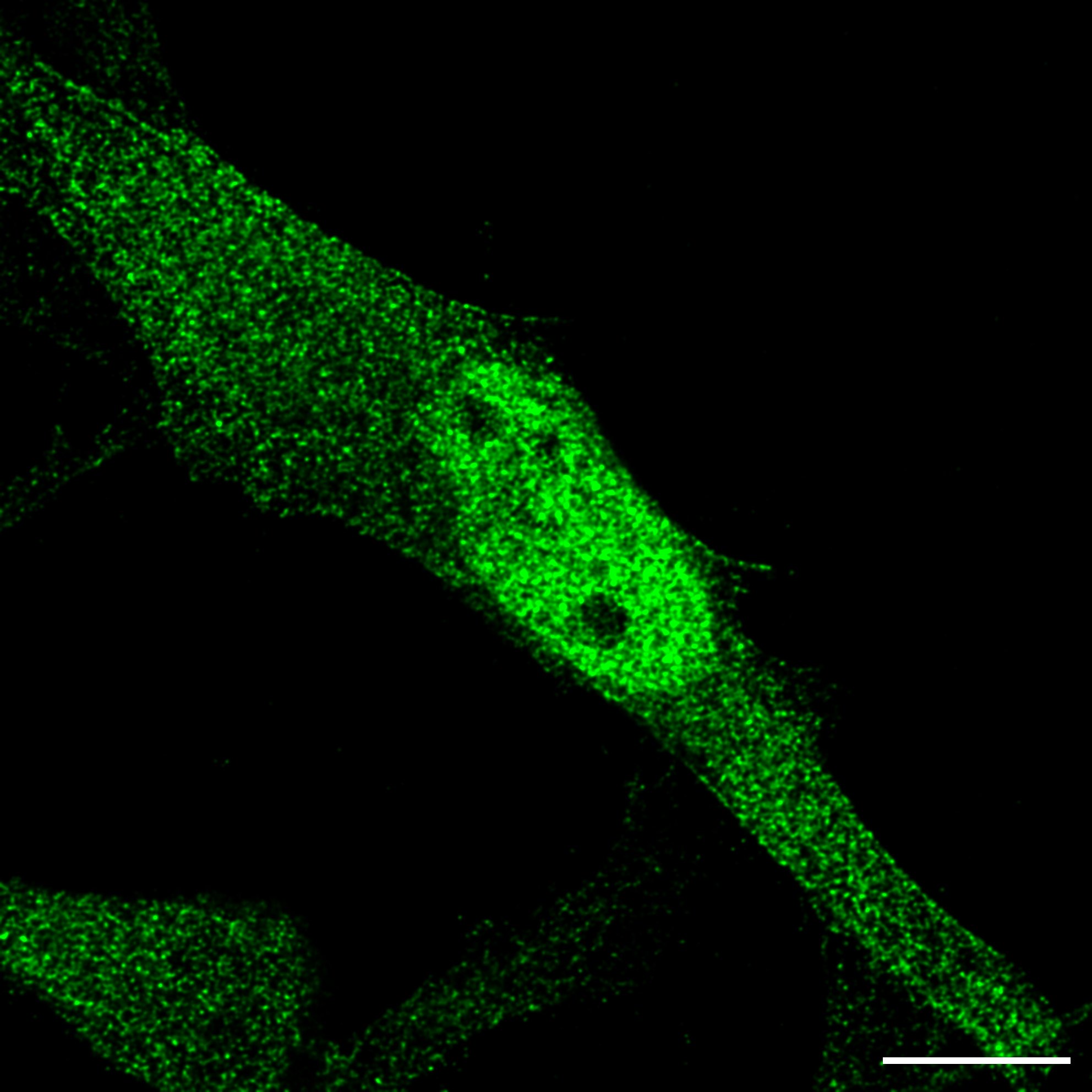

Supplement: Supplementary file 13 — Appendix Figures Source Data [file 44319_2024_58_MOESM13_ESM.zip › Appendix source data/Fig S3 Source data/Fig S3C/Fig S3C WT-TFEB FLAG - TFEB.jpg]

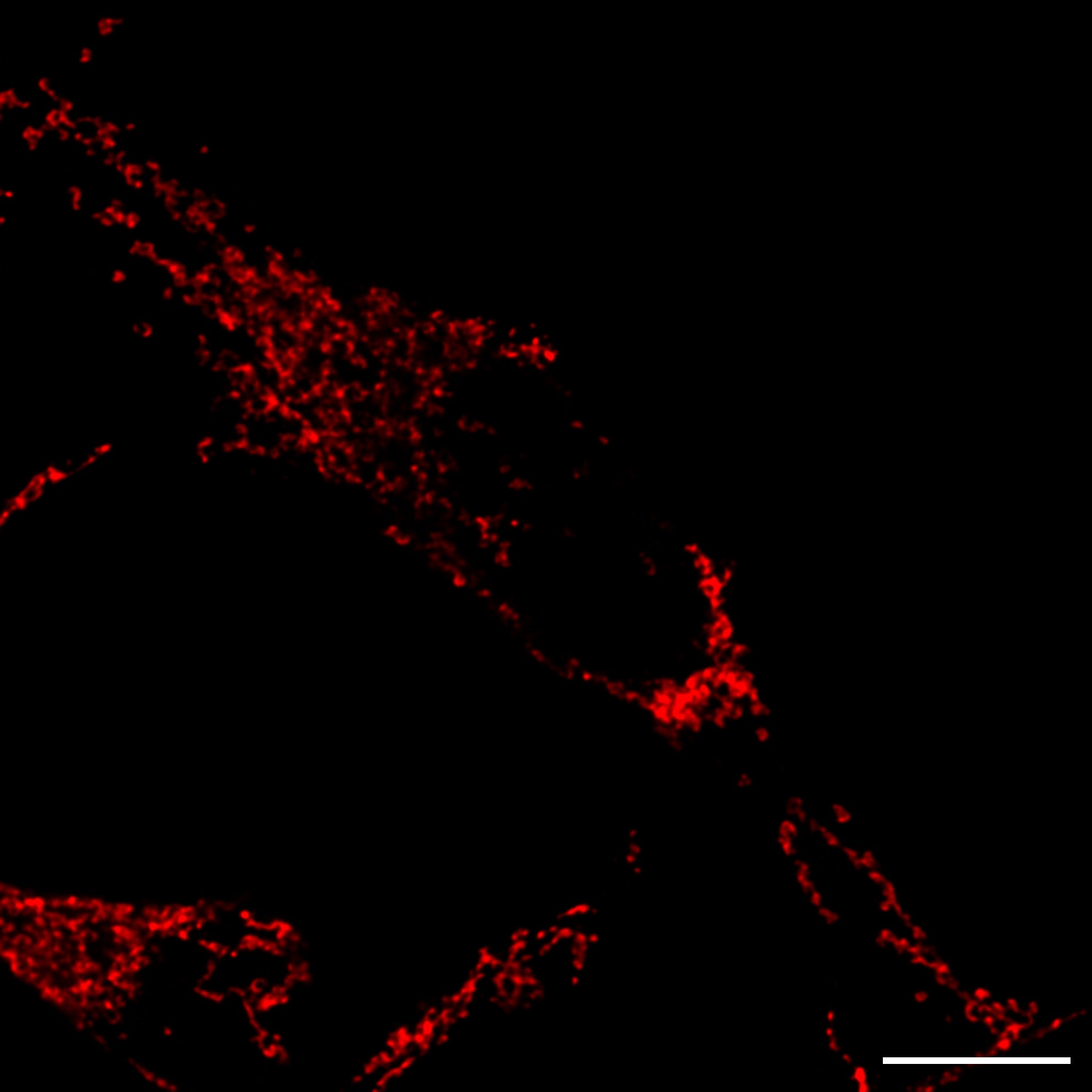

Supplement: Supplementary file 13 — Appendix Figures Source Data [file 44319_2024_58_MOESM13_ESM.zip › Appendix source data/Fig S3 Source data/Fig S3C/Fig S3C WT-TFEB FLAG - TOMM20.jpg]

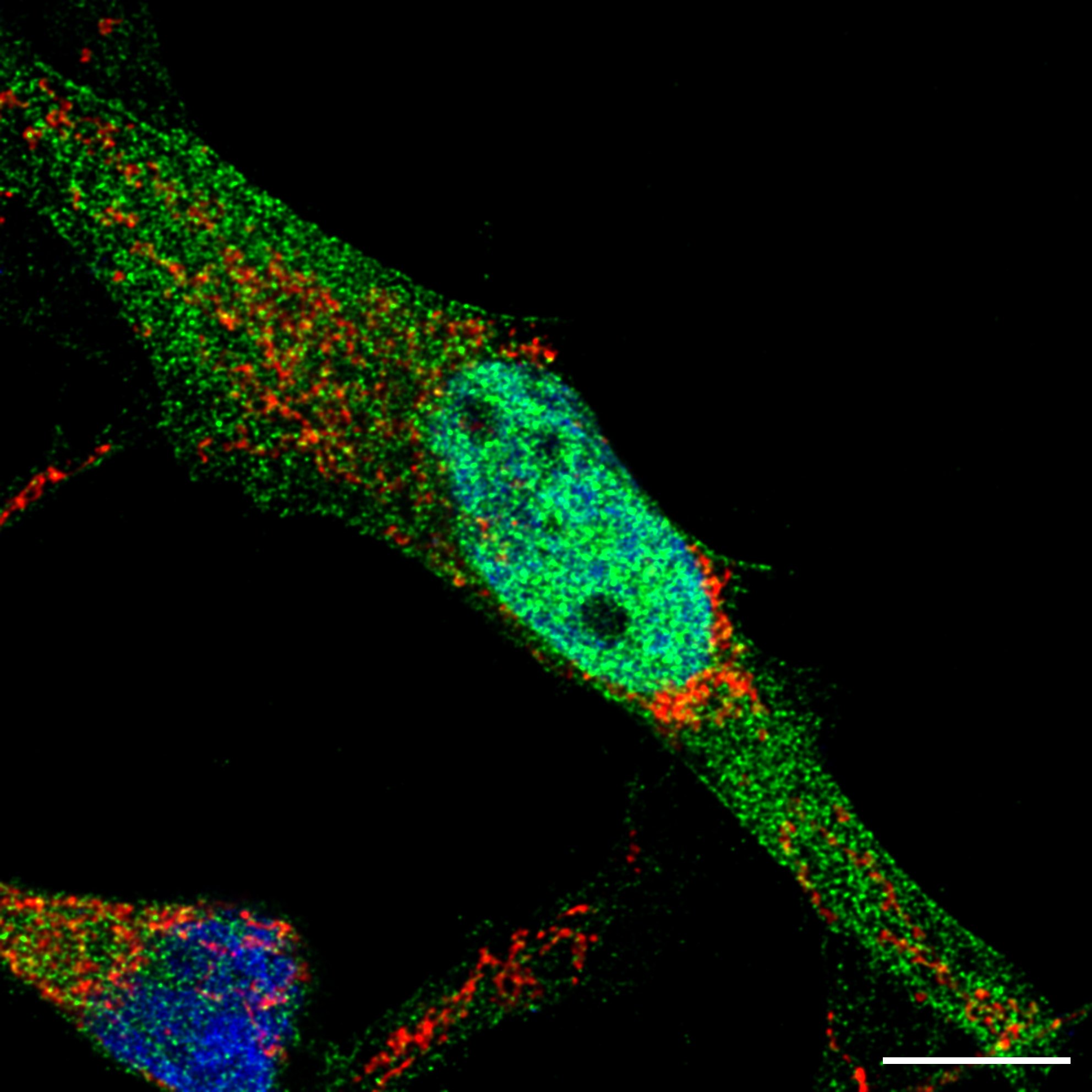

Supplement: Supplementary file 13 — Appendix Figures Source Data [file 44319_2024_58_MOESM13_ESM.zip › Appendix source data/Fig S3 Source data/Fig S3C/Fig S3C WT-TFEB FLAG - Merged.jpg]

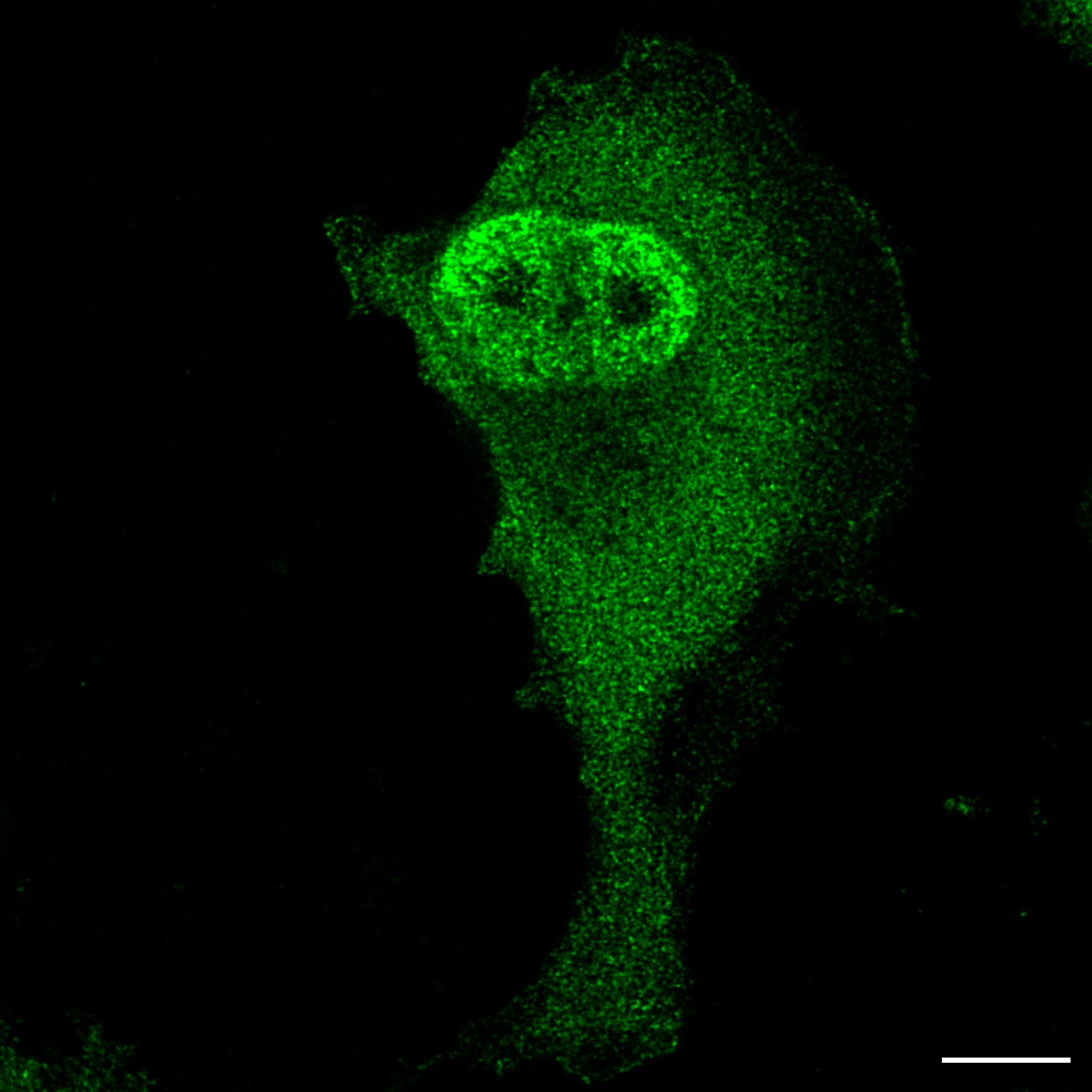

Supplement: Supplementary file 13 — Appendix Figures Source Data [file 44319_2024_58_MOESM13_ESM.zip › Appendix source data/Fig S3 Source data/Fig S3C/Fig S3C MLS-TFEB FLAG - TFEB.jpg]

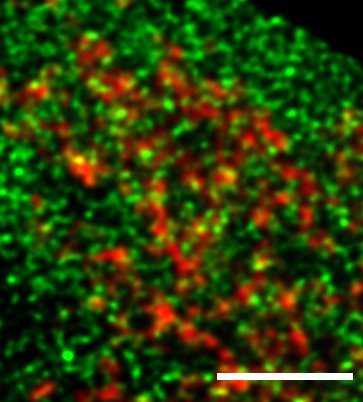

Supplement: Supplementary file 13 — Appendix Figures Source Data [file 44319_2024_58_MOESM13_ESM.zip › Appendix source data/Fig S3 Source data/Fig S3C/Fig S3C WT-TFEB FLAG - Inset Merged.jpg]

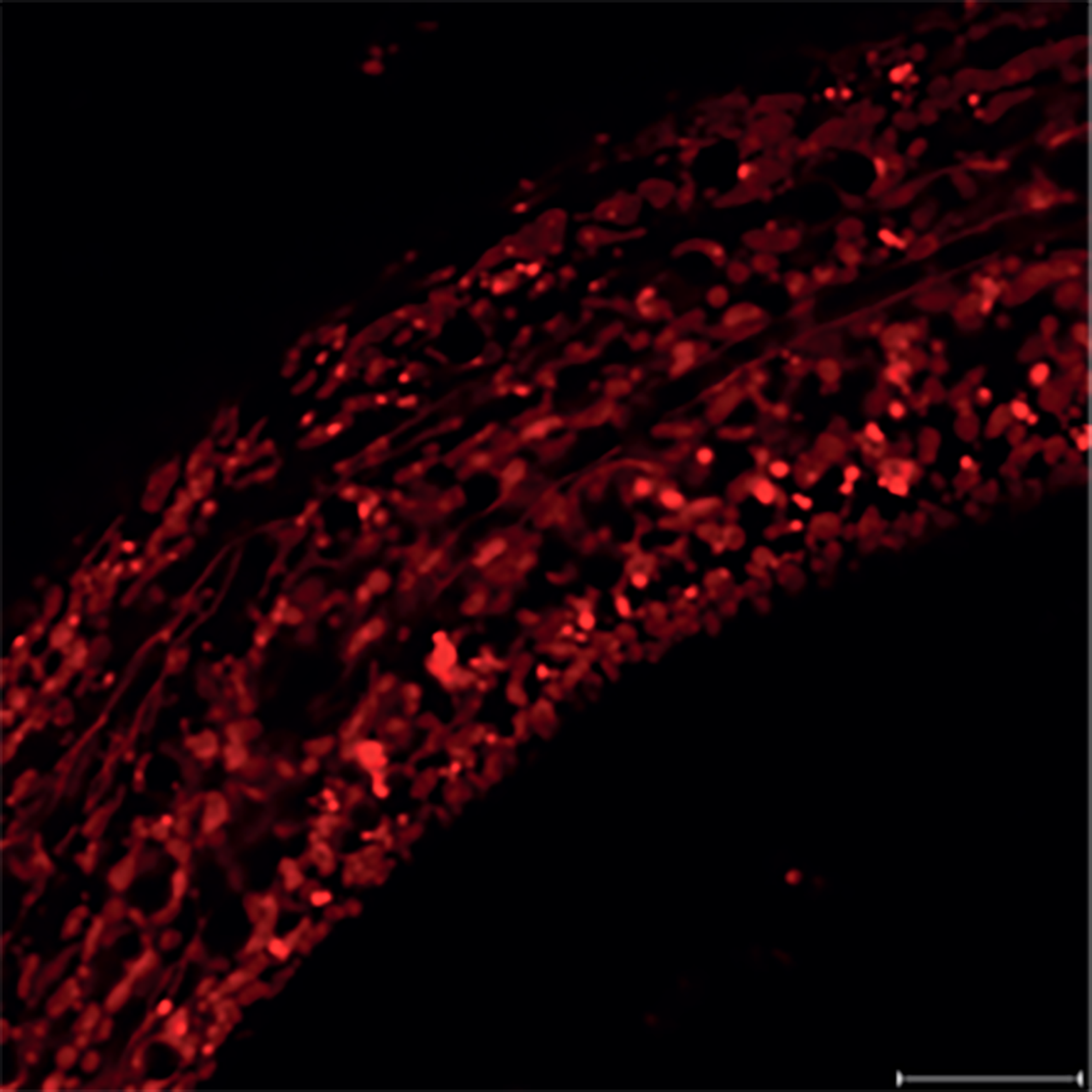

Supplement: Supplementary file 13 — Appendix Figures Source Data [file 44319_2024_58_MOESM13_ESM.zip › Appendix source data/Fig S3 Source data/Fig S3E/Fig S3E -Mitotracket_2.tif]

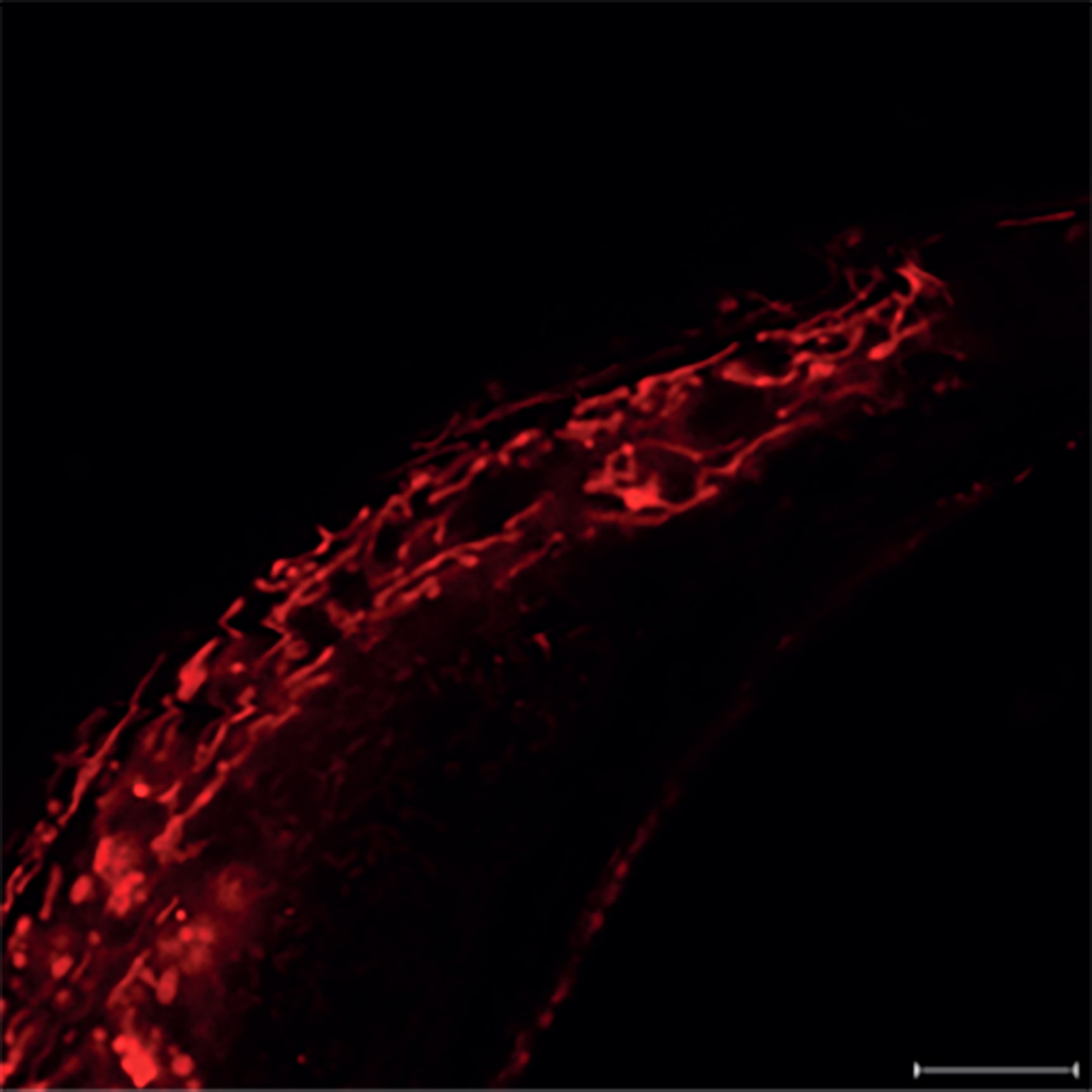

Supplement: Supplementary file 13 — Appendix Figures Source Data [file 44319_2024_58_MOESM13_ESM.zip › Appendix source data/Fig S3 Source data/Fig S3E/Fig S3E -Mitotracker_1.tif]

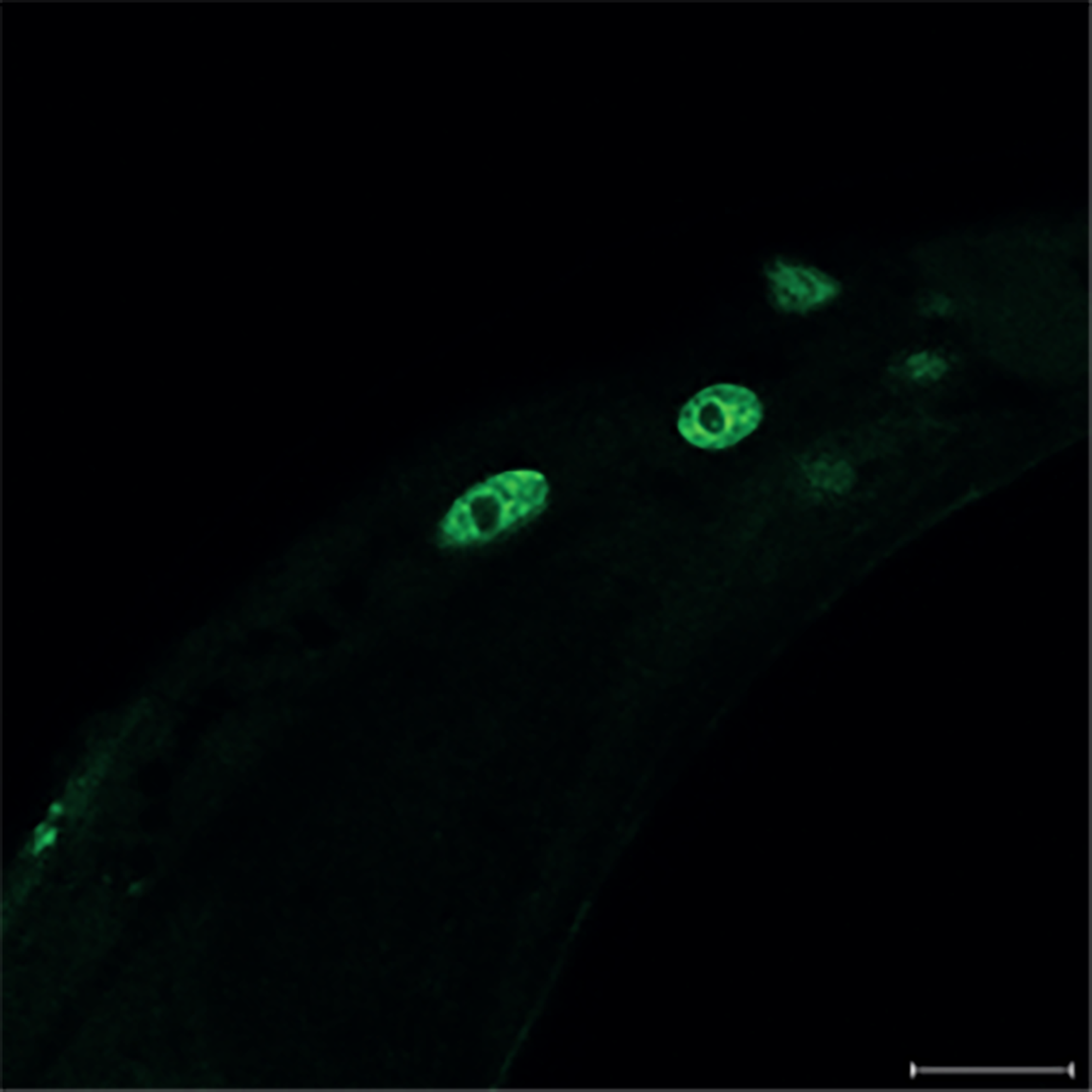

Supplement: Supplementary file 13 — Appendix Figures Source Data [file 44319_2024_58_MOESM13_ESM.zip › Appendix source data/Fig S3 Source data/Fig S3E/Fig S3E - HLH30_1.tif]

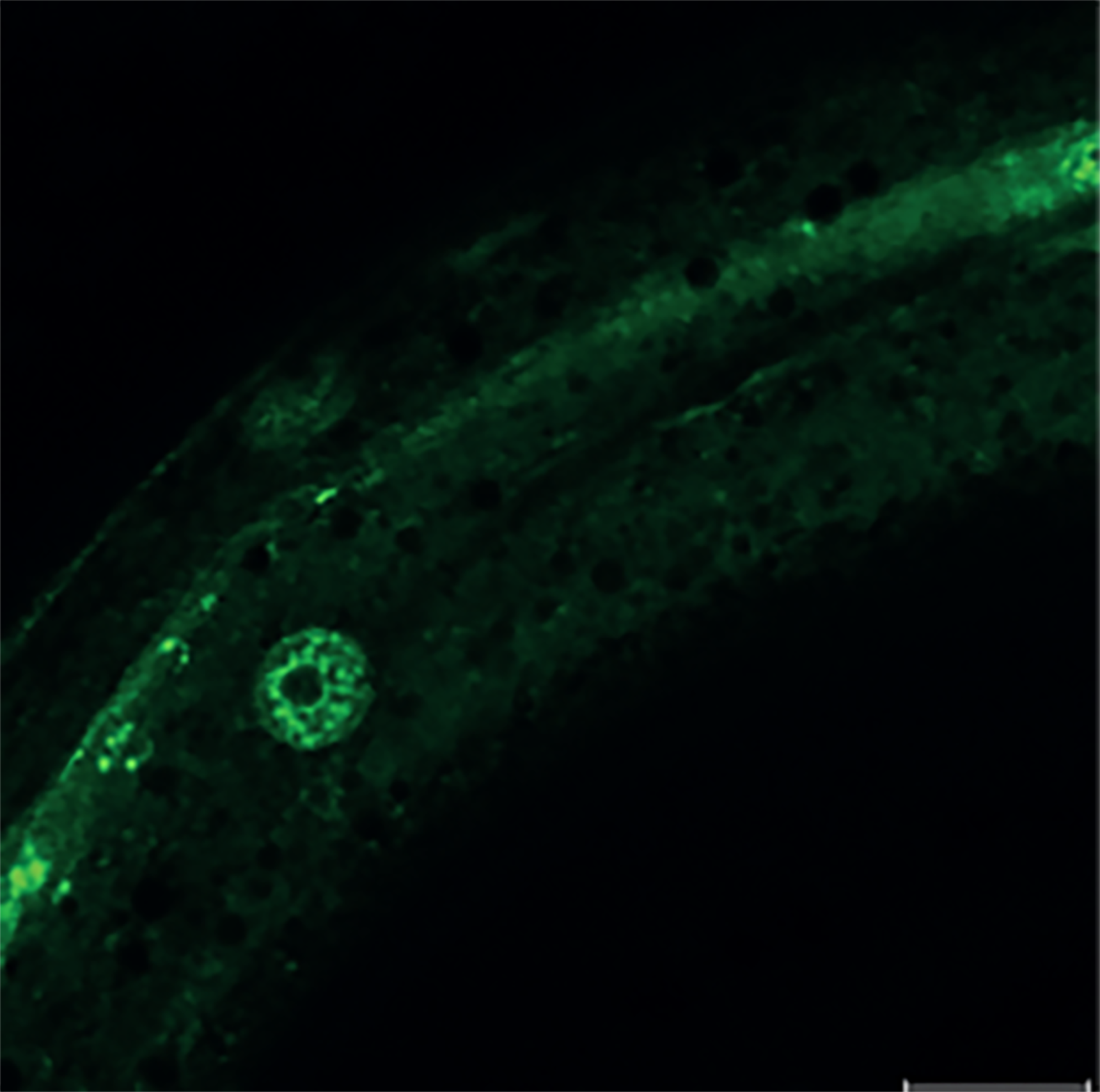

Supplement: Supplementary file 13 — Appendix Figures Source Data [file 44319_2024_58_MOESM13_ESM.zip › Appendix source data/Fig S3 Source data/Fig S3E/Fig S3E - HLH30_2.tif]

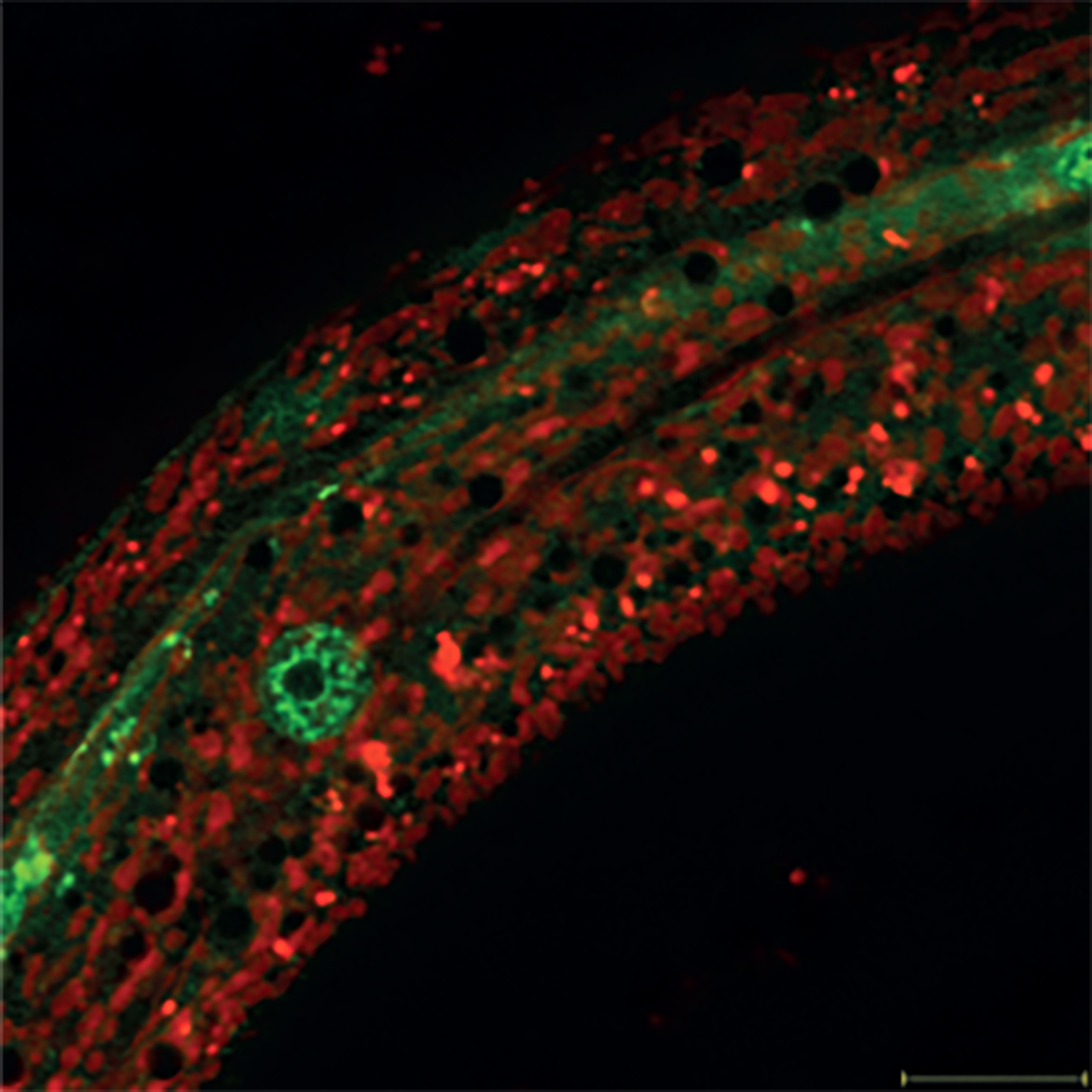

Supplement: Supplementary file 13 — Appendix Figures Source Data [file 44319_2024_58_MOESM13_ESM.zip › Appendix source data/Fig S3 Source data/Fig S3E/Fig S3E -Merged_2.tif]

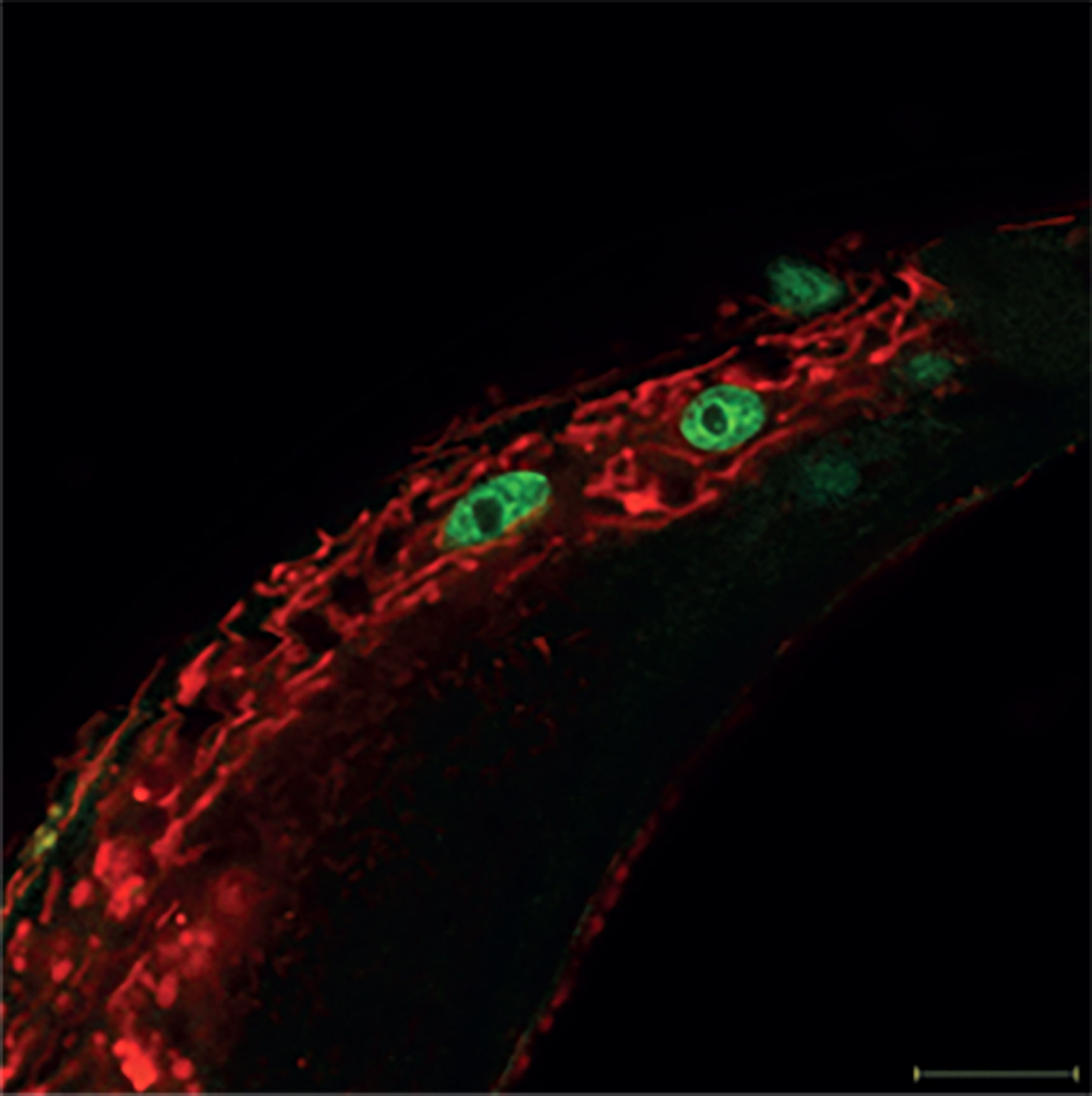

Supplement: Supplementary file 13 — Appendix Figures Source Data [file 44319_2024_58_MOESM13_ESM.zip › Appendix source data/Fig S3 Source data/Fig S3E/Fig S3E -Merged_1.tif]

Fig. S3A

IB:FLAG

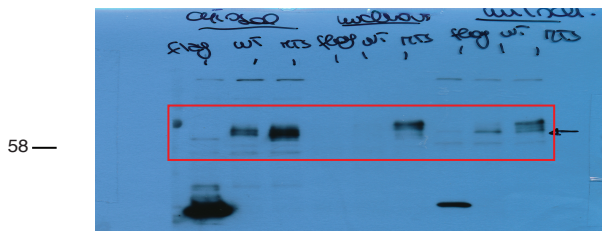

IB:GAPDH

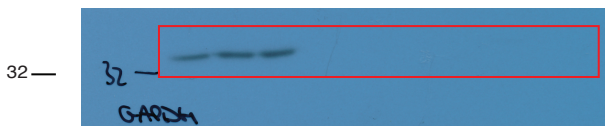

IB:LAMINB

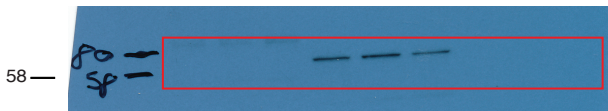

IB:TOMM20

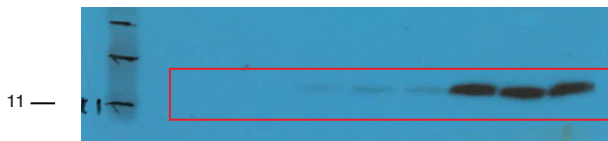

Supplement: Supplementary file 13 — Appendix Figures Source Data [file 44319_2024_58_MOESM13_ESM.zip › Appendix source data/Fig S3 Source data/Fig S3A/Fig S3A Unprocessed blots.pdf]

**Fig. S6A**

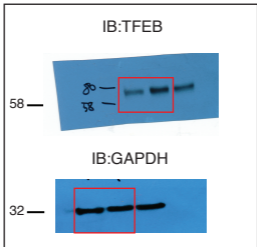

Supplement: Supplementary file 13 — Appendix Figures Source Data [file 44319_2024_58_MOESM13_ESM.zip › Appendix source data/Fig S6 Source data/Fig S6A/Fig S6A Unprocessed blots.pdf]

**Fig. S6F**

IB:CTRL/TFEB BEADS

IB:LONP1

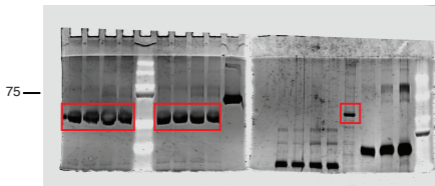

Supplement: Supplementary file 13 — Appendix Figures Source Data [file 44319_2024_58_MOESM13_ESM.zip › Appendix source data/Fig S6 Source data/Fig S6F/Fig S6F unprocessed blots.pdf]

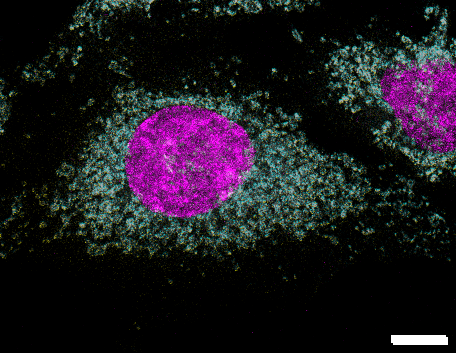

Supplement: Supplementary file 13 — Appendix Figures Source Data [file 44319_2024_58_MOESM13_ESM.zip › Appendix source data/Fig S5 Source data/Fig S5F/TOMM20-PINK1_merge_MLS-TFEB-FLAG.tif]

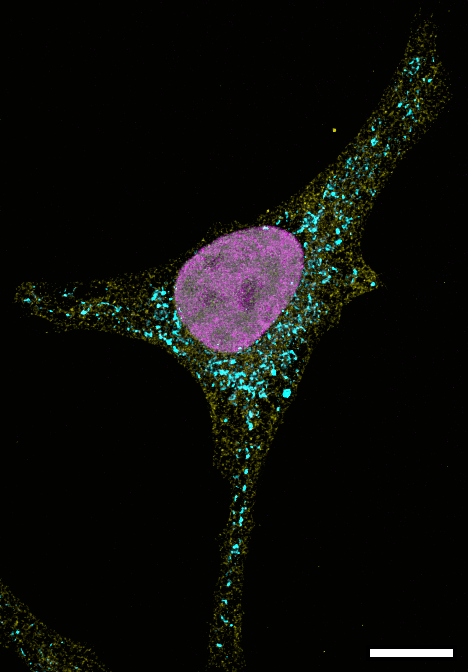

Supplement: Supplementary file 13 — Appendix Figures Source Data [file 44319_2024_58_MOESM13_ESM.zip › Appendix source data/Fig S5 Source data/Fig S5F/TOMM20-PINK1_merge_NLS-TFEB-FLAG.tif]

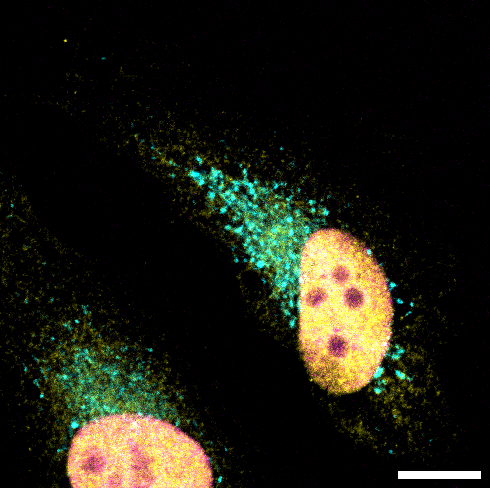

Supplement: Supplementary file 13 — Appendix Figures Source Data [file 44319_2024_58_MOESM13_ESM.zip › Appendix source data/Fig S5 Source data/Fig S5F/TOMM20-PINK1_merge_shTFEB-FLAG.tif]

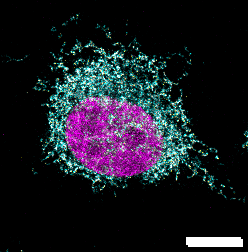

Supplement: Supplementary file 13 — Appendix Figures Source Data [file 44319_2024_58_MOESM13_ESM.zip › Appendix source data/Fig S5 Source data/Fig S5F/TOMM20-PINK1_merge_WT-TFEB-FLAG.tif]

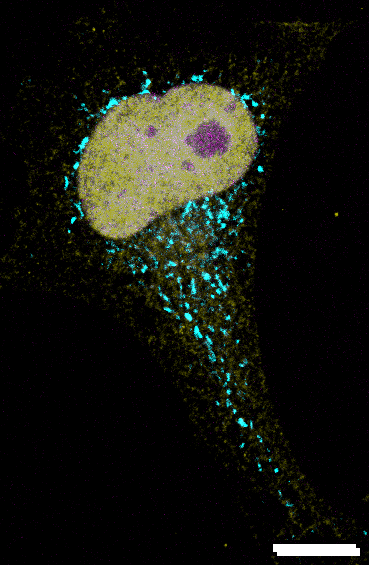

Supplement: Supplementary file 13 — Appendix Figures Source Data [file 44319_2024_58_MOESM13_ESM.zip › Appendix source data/Fig S5 Source data/Fig S5F/TOMM20-PINK1_merge_MTS-TFEB-FLAG.tif]

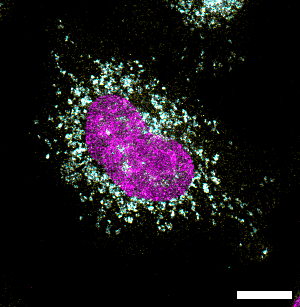

Supplement: Supplementary file 13 — Appendix Figures Source Data [file 44319_2024_58_MOESM13_ESM.zip › Appendix source data/Fig S5 Source data/Fig S5F/TOMM20-PINK1_merge_S142A:S211A TFEB-FLAG.tif]

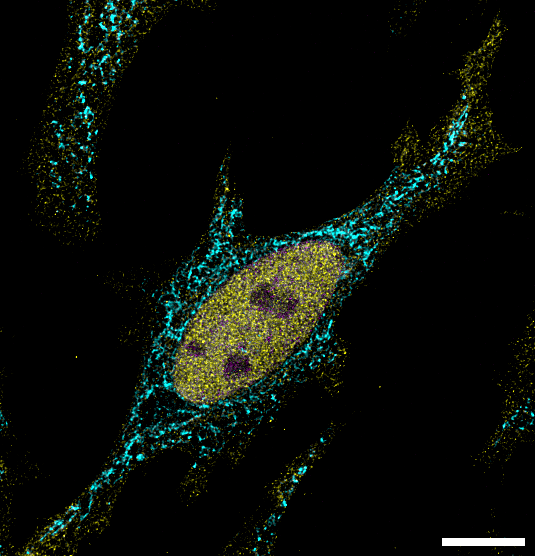

Supplement: Supplementary file 13 — Appendix Figures Source Data [file 44319_2024_58_MOESM13_ESM.zip › Appendix source data/Fig S5 Source data/Fig S5F/TOMM20-PINK1_sh.tif]

**Fig. S5D**

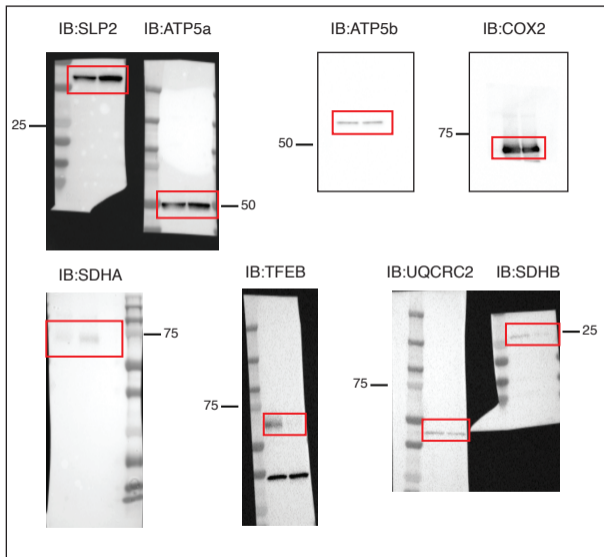

Supplement: Supplementary file 13 — Appendix Figures Source Data [file 44319_2024_58_MOESM13_ESM.zip › Appendix source data/Fig S5 Source data/Fig S5D/Fig S5D Unprocessed blots.pdf]

**Fig. S7D**

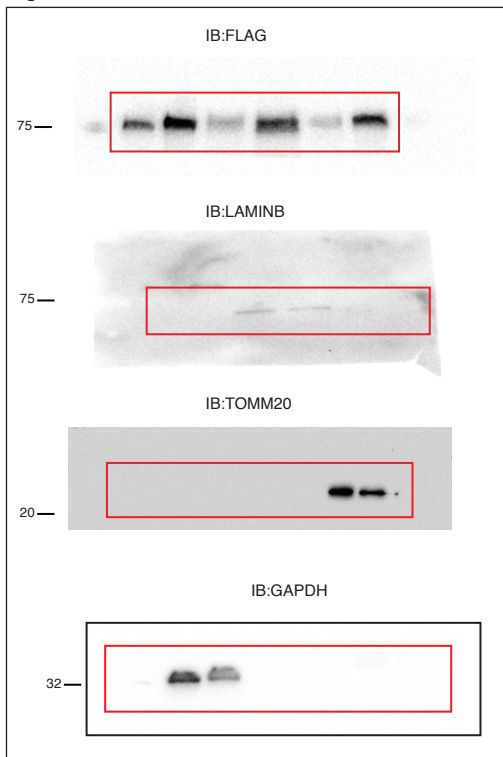

Supplement: Supplementary file 13 — Appendix Figures Source Data [file 44319_2024_58_MOESM13_ESM.zip › Appendix source data/Fig S7 Source data/Fig S7D/Fig S7D Unprocessed blots.pdf]

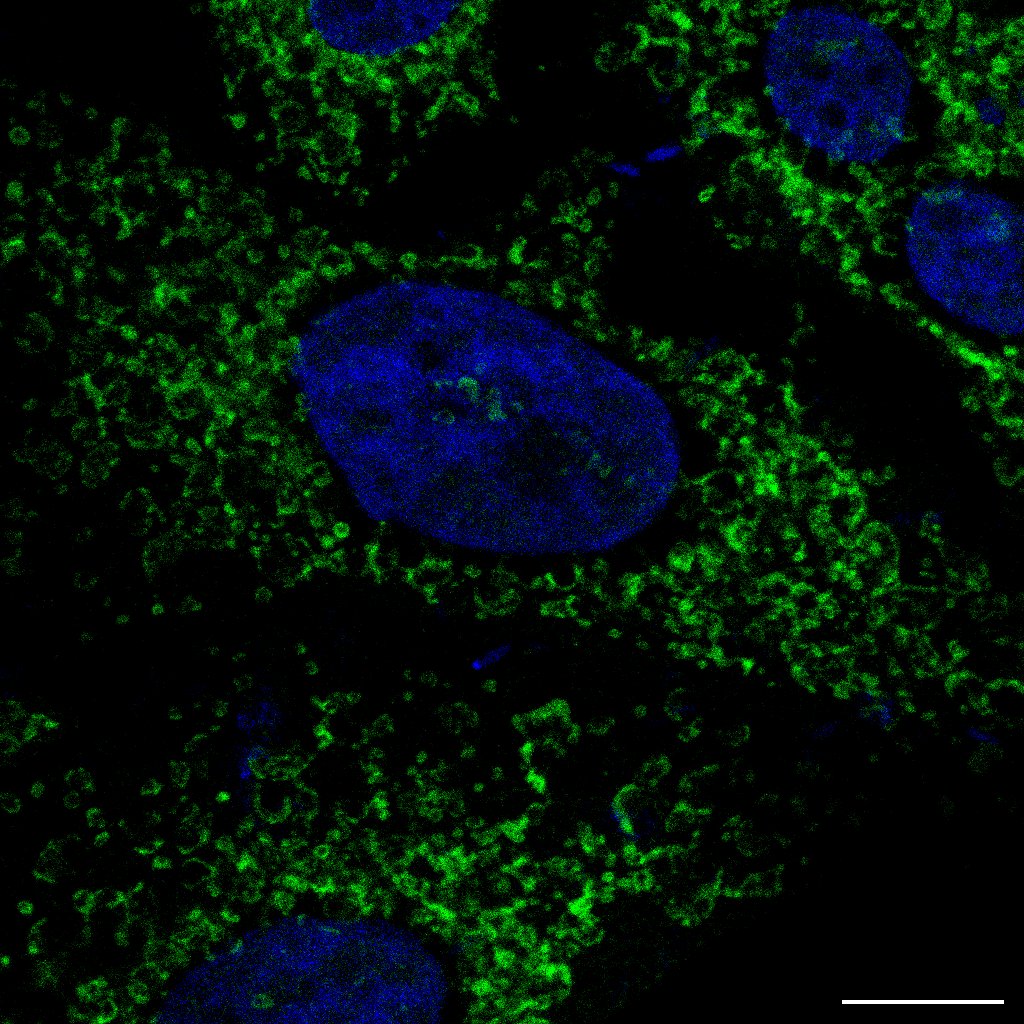

Supplement: Supplementary file 13 — Appendix Figures Source Data [file 44319_2024_58_MOESM13_ESM.zip › Appendix source data/Fig S7 Source data/Fig S7A/Fig S7A UT - Merged.jpg]

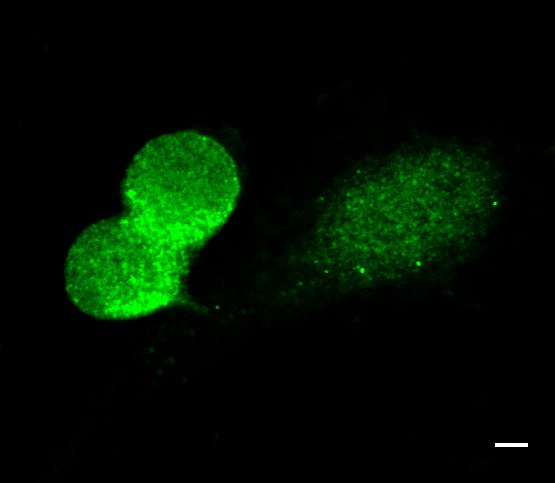

Supplement: Supplementary file 13 — Appendix Figures Source Data [file 44319_2024_58_MOESM13_ESM.zip › Appendix source data/Fig S7 Source data/Fig S7A/Fig S7A torin1 - TFEB.jpg]

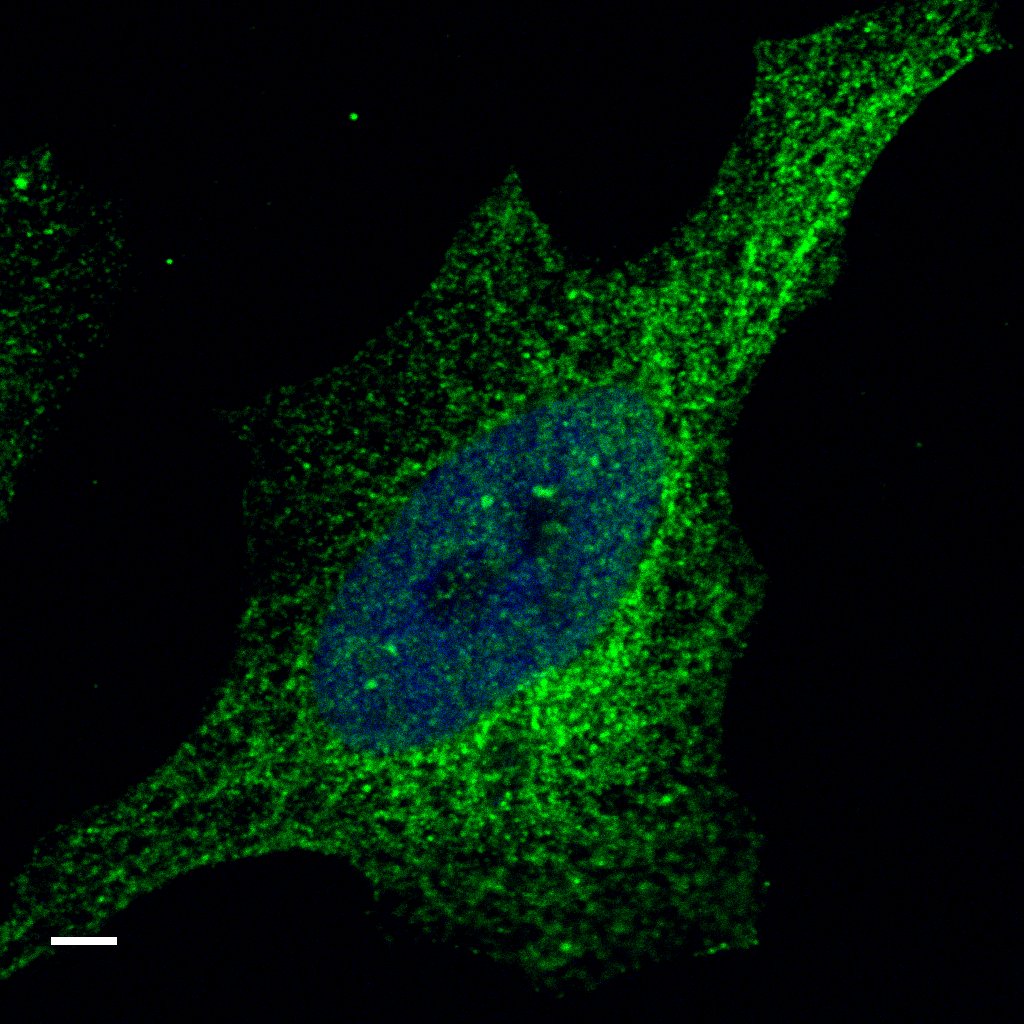

Supplement: Supplementary file 13 — Appendix Figures Source Data [file 44319_2024_58_MOESM13_ESM.zip › Appendix source data/Fig S7 Source data/Fig S7A/Fig S7A ST - TFEB.jpg]

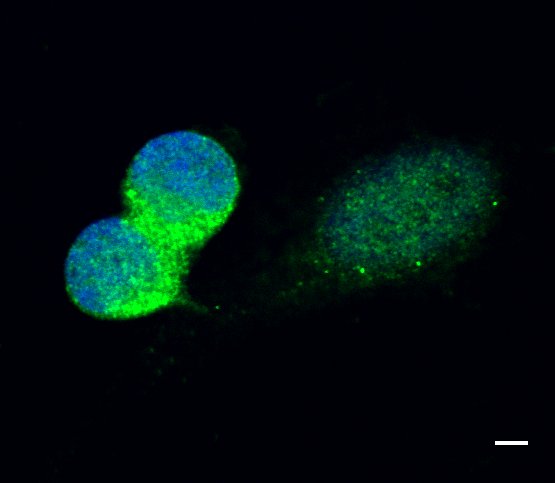

Supplement: Supplementary file 13 — Appendix Figures Source Data [file 44319_2024_58_MOESM13_ESM.zip › Appendix source data/Fig S7 Source data/Fig S7A/Fig S7A torin1 - Merged.jpg]

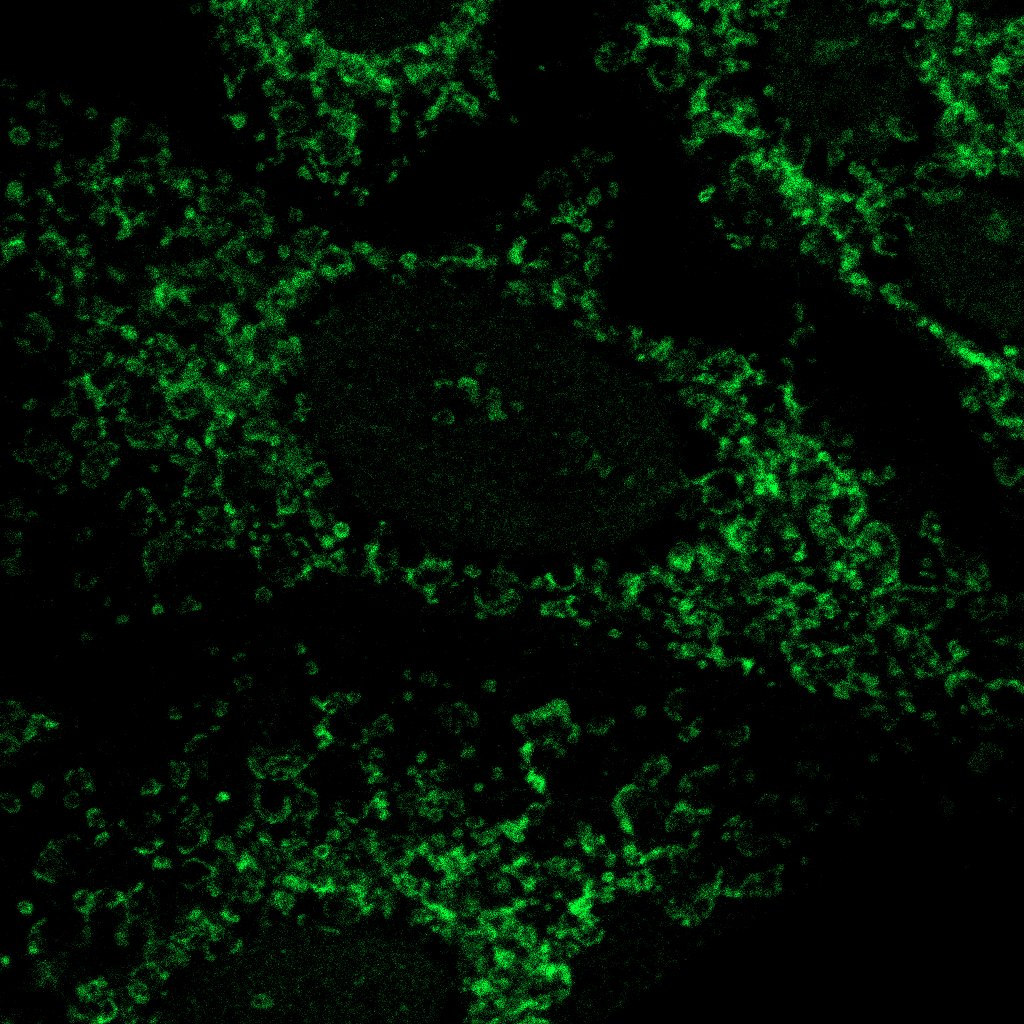

Supplement: Supplementary file 13 — Appendix Figures Source Data [file 44319_2024_58_MOESM13_ESM.zip › Appendix source data/Fig S7 Source data/Fig S7A/Fig S7A UT - TFEB.jpg]

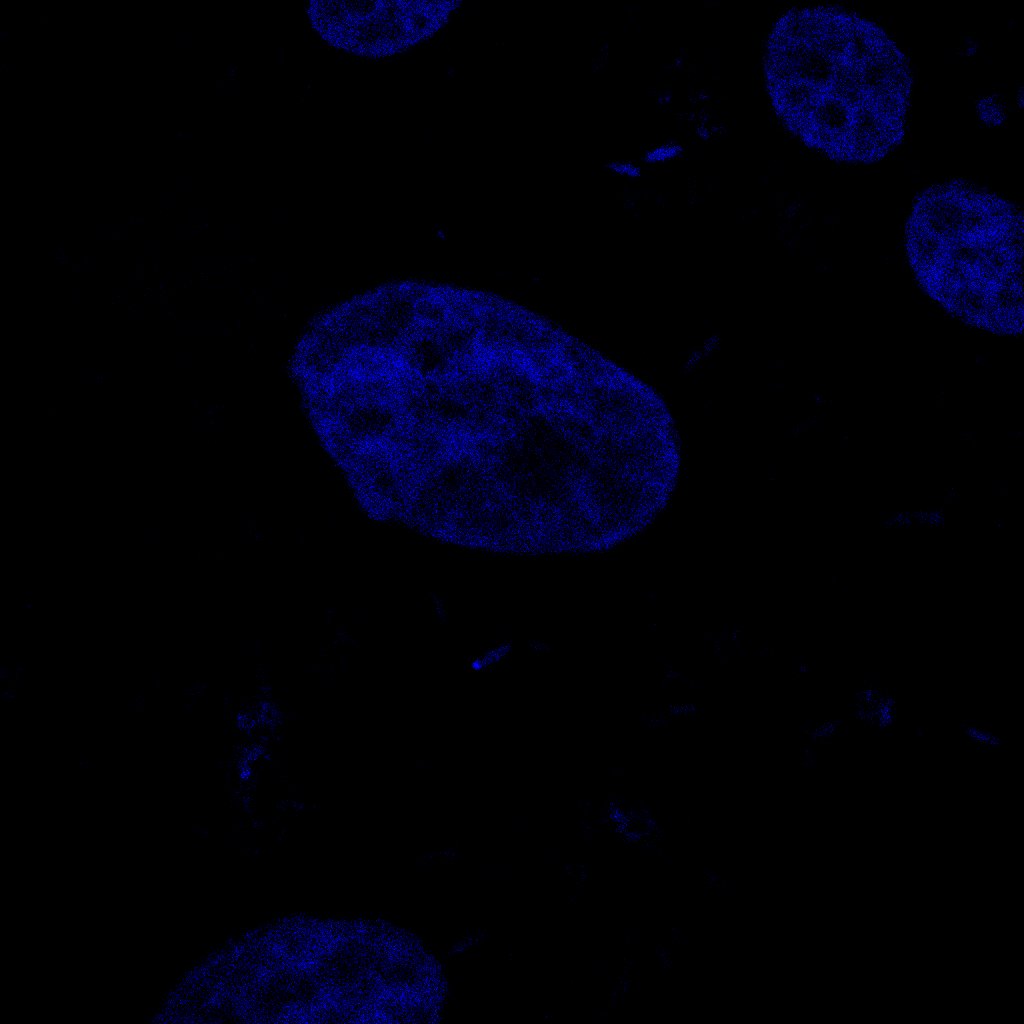

Supplement: Supplementary file 13 — Appendix Figures Source Data [file 44319_2024_58_MOESM13_ESM.zip › Appendix source data/Fig S7 Source data/Fig S7A/Fig S7A UT - DAPI.jpg]

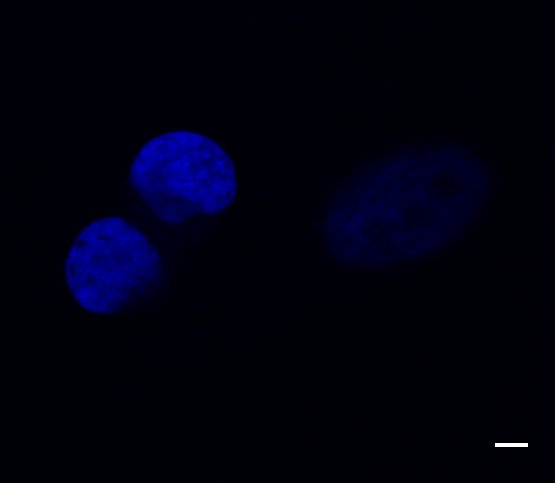

Supplement: Supplementary file 13 — Appendix Figures Source Data [file 44319_2024_58_MOESM13_ESM.zip › Appendix source data/Fig S7 Source data/Fig S7A/Fig S7A torin1 - DAPI.jpg]

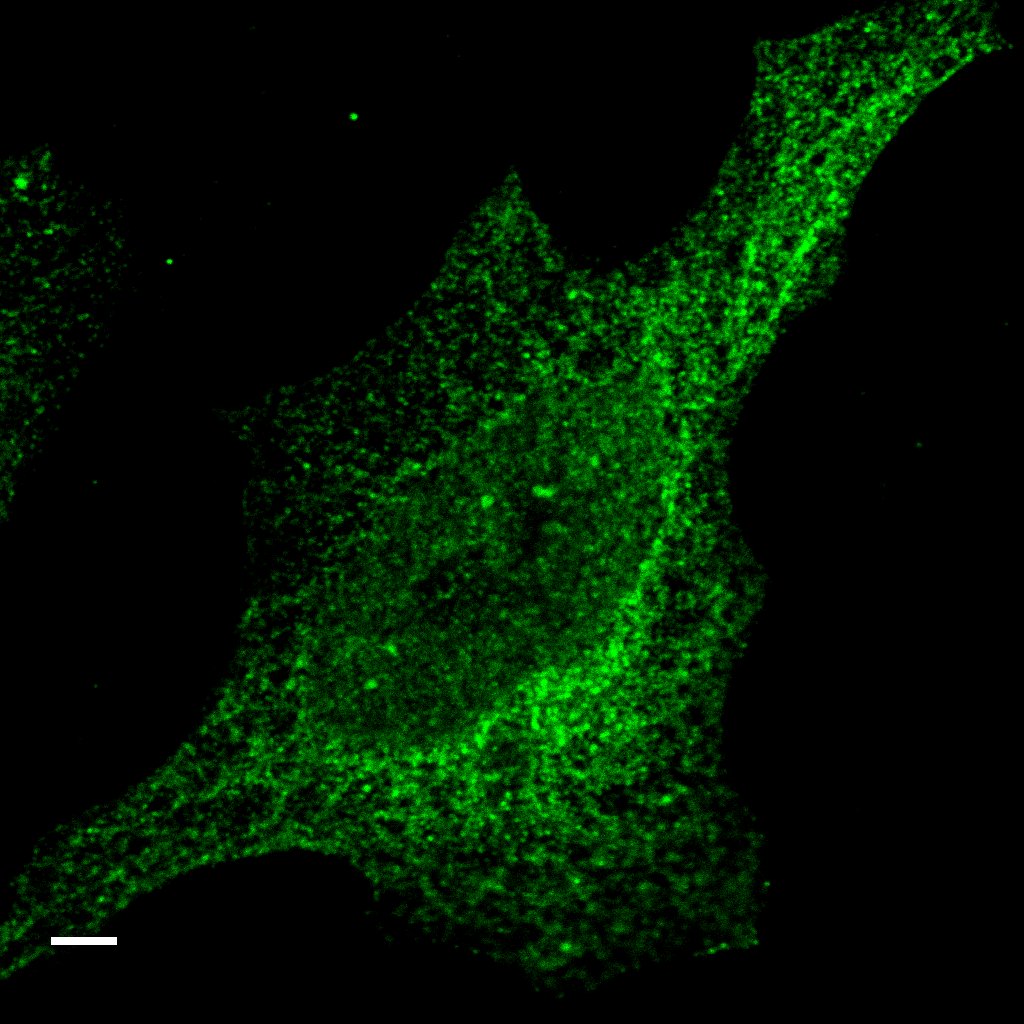

Supplement: Supplementary file 13 — Appendix Figures Source Data [file 44319_2024_58_MOESM13_ESM.zip › Appendix source data/Fig S7 Source data/Fig S7A/Fig S7A ST - Merged.jpg]

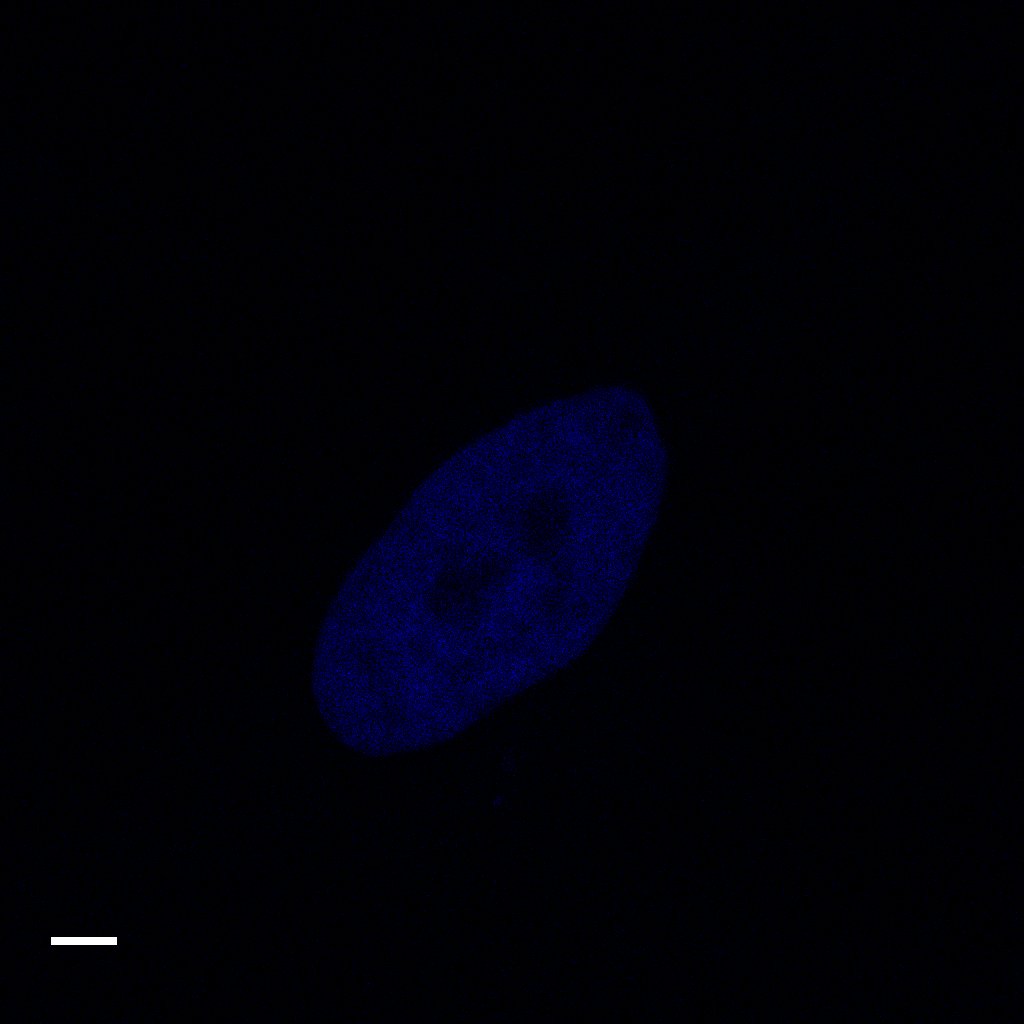

Supplement: Supplementary file 13 — Appendix Figures Source Data [file 44319_2024_58_MOESM13_ESM.zip › Appendix source data/Fig S7 Source data/Fig S7A/Fig S7A ST - DAPI.jpg]

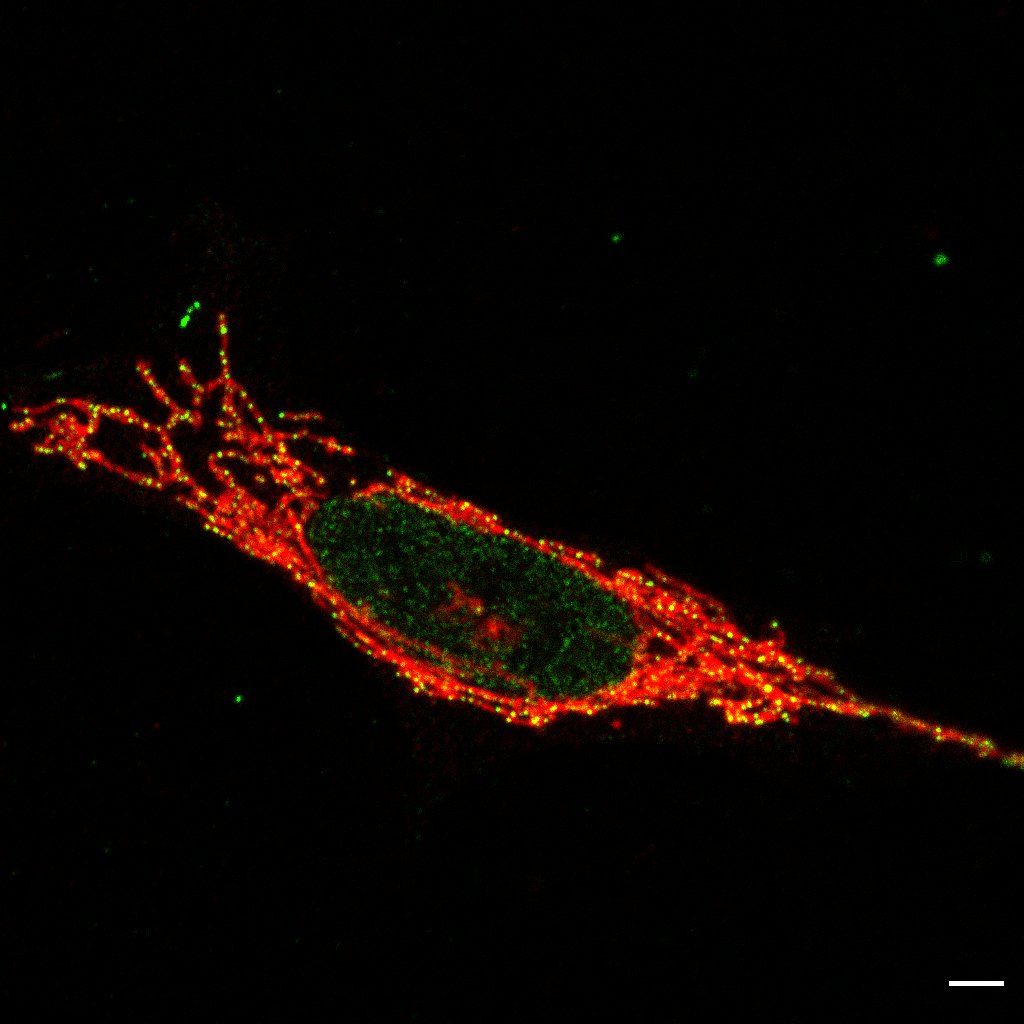

Supplement: Supplementary file 13 — Appendix Figures Source Data [file 44319_2024_58_MOESM13_ESM.zip › Appendix source data/Fig S2 Source data/Fig S2A/Fig S2A - Merged.jpg]

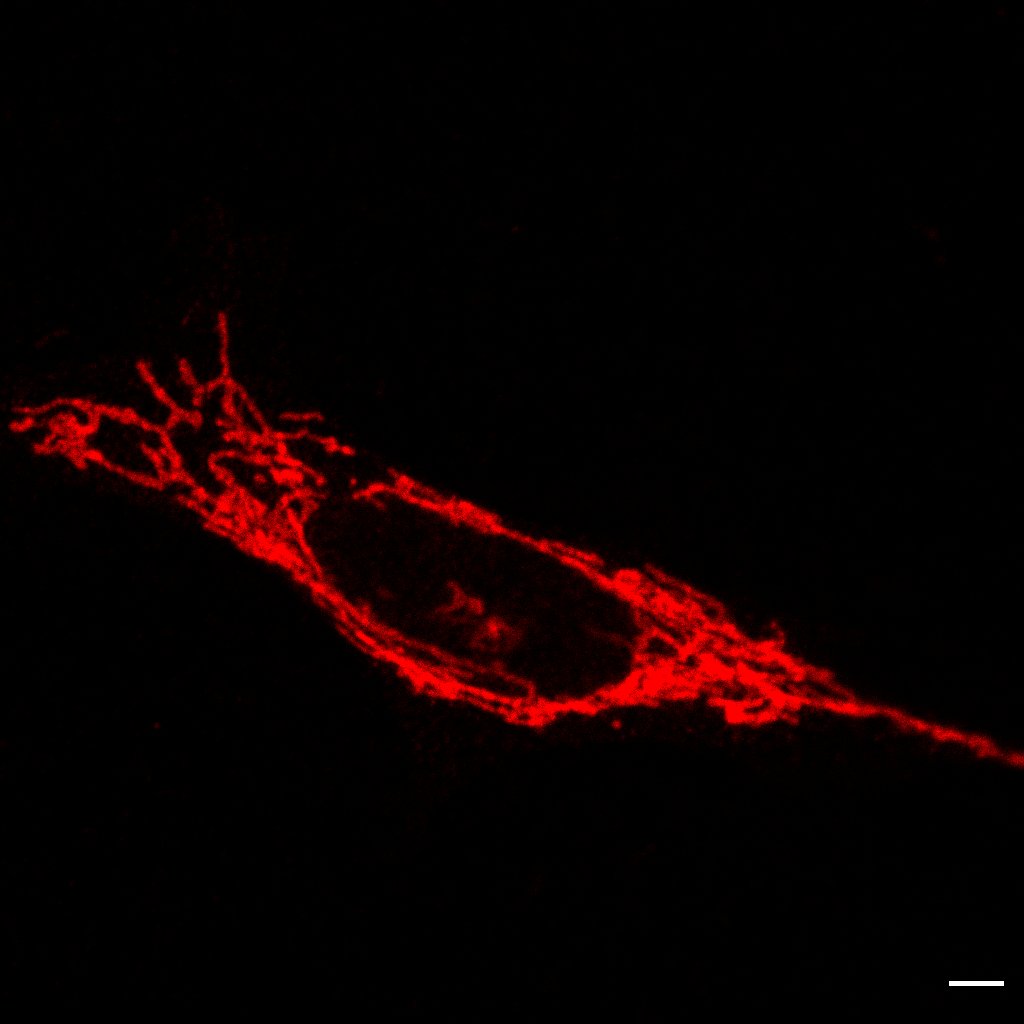

Supplement: Supplementary file 13 — Appendix Figures Source Data [file 44319_2024_58_MOESM13_ESM.zip › Appendix source data/Fig S2 Source data/Fig S2A/Fig S2A - TOMM20.jpg]

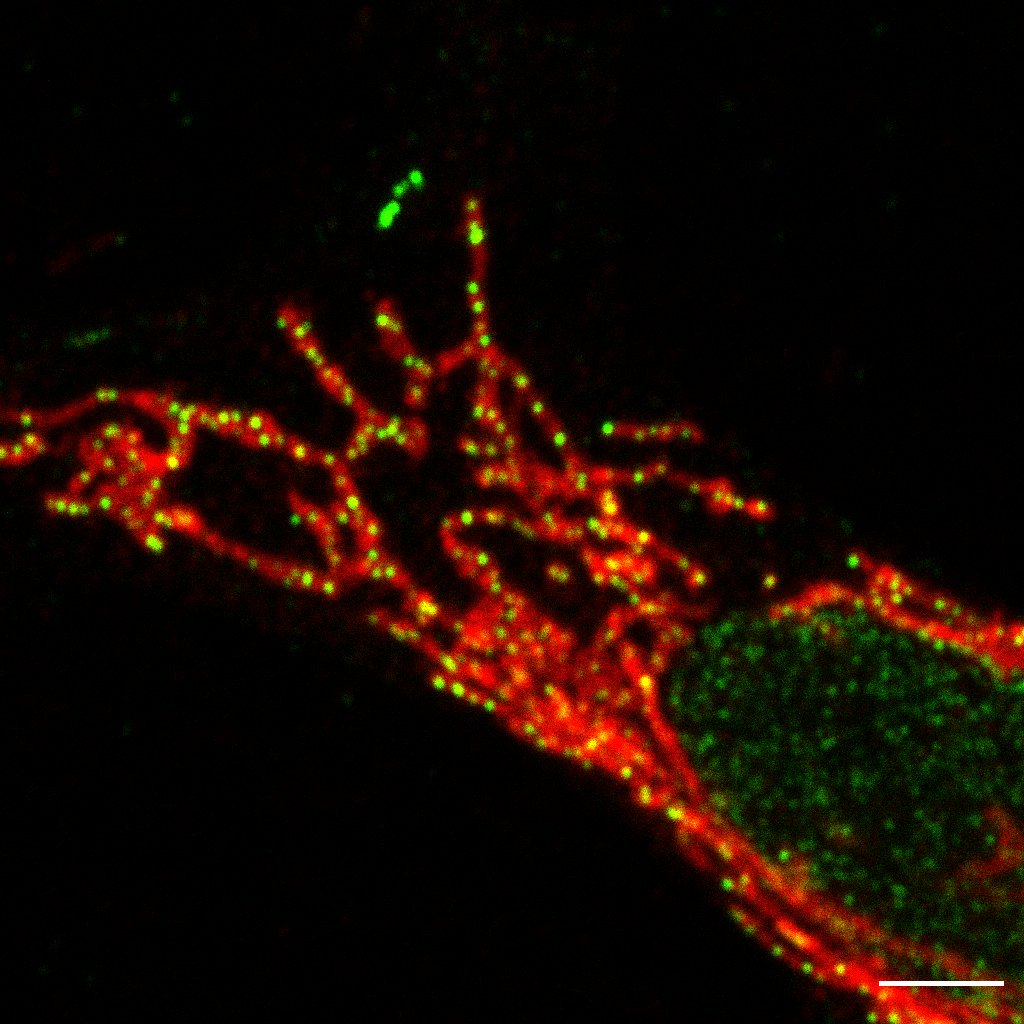

Supplement: Supplementary file 13 — Appendix Figures Source Data [file 44319_2024_58_MOESM13_ESM.zip › Appendix source data/Fig S2 Source data/Fig S2A/Fig S2A - Inset merged.jpg]

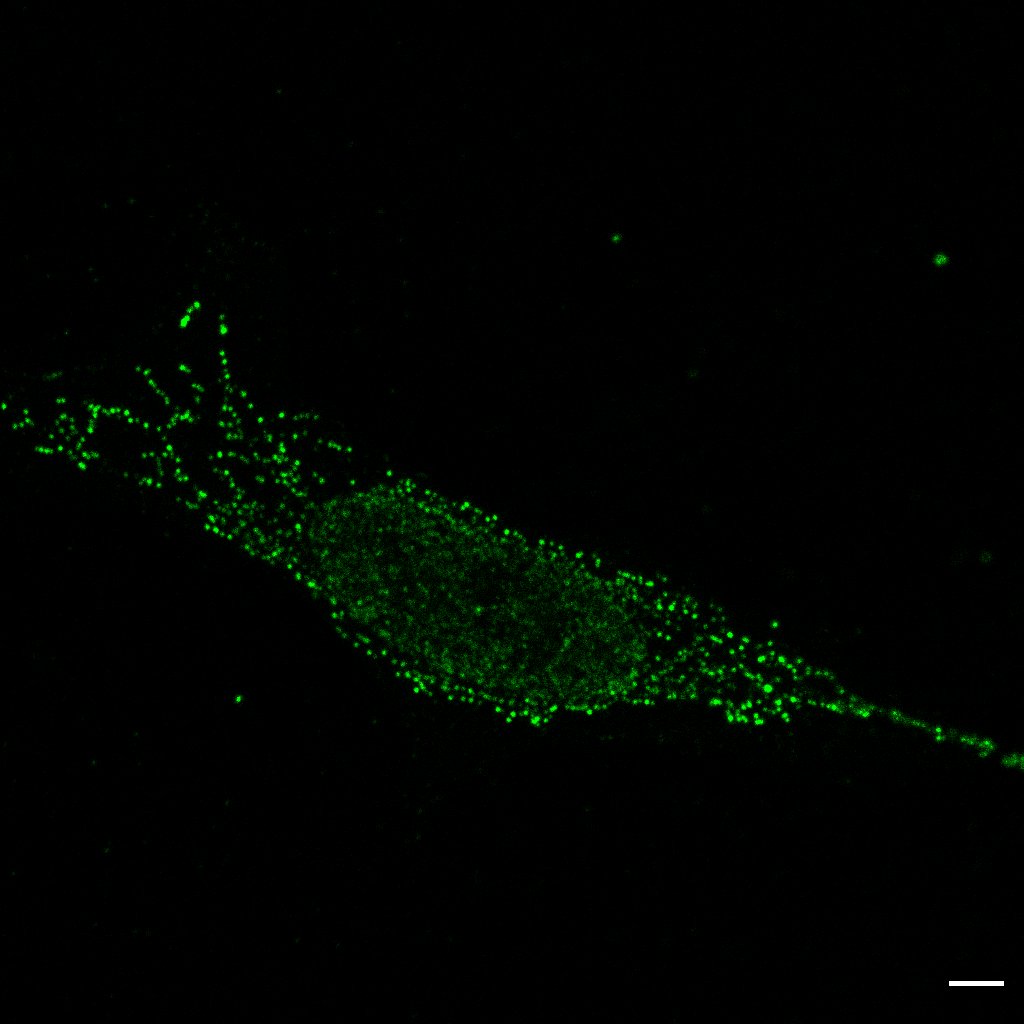

Supplement: Supplementary file 13 — Appendix Figures Source Data [file 44319_2024_58_MOESM13_ESM.zip › Appendix source data/Fig S2 Source data/Fig S2A/Fig S2A - mtDNA.jpg]

**Fig. S2B**

IB:TFEB

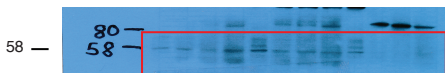

IB:MRPL12

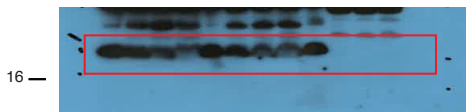

IB:TIMM23

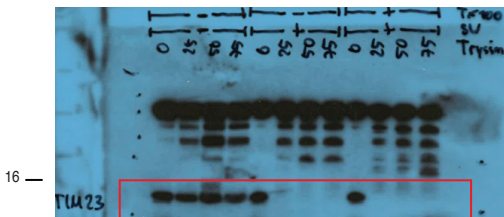

IB:TOMM20

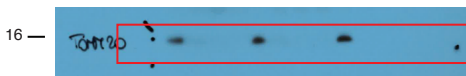

**Fig. S2B**

IB:TFEB

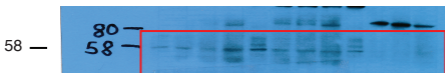

IB:MRPL12

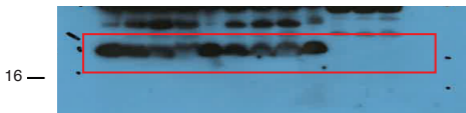

IB:TIMM23

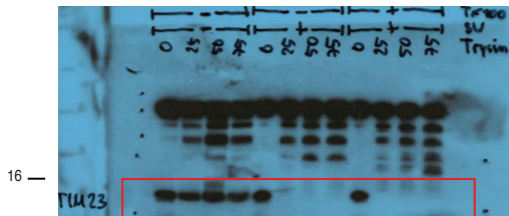

IB:TOMM20

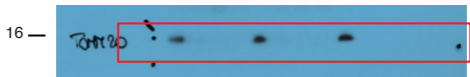

Supplement: Supplementary file 13 — Appendix Figures Source Data [file 44319_2024_58_MOESM13_ESM.zip › Appendix source data/Fig S2 Source data/Fig S2B/Fig S2B Unprocessed blots.pdf]

**Fig. S1G**

IB: MITF

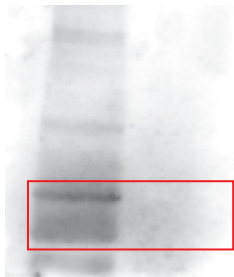

IB: TFE3

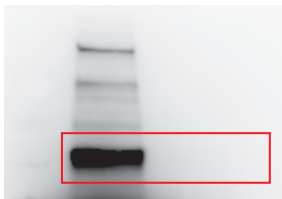

IB: TOMM20

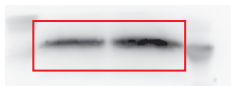

IB: GAPDH

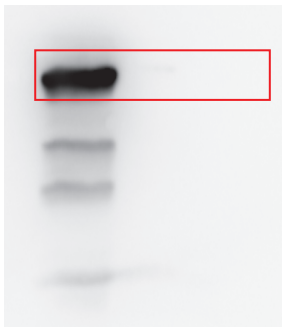

Supplement: Supplementary file 13 — Appendix Figures Source Data [file 44319_2024_58_MOESM13_ESM.zip › Appendix source data/Fig S1 Source data/Fig S1G/Fig S1G unprocessed blots.pdf]

**Fig. S1F**

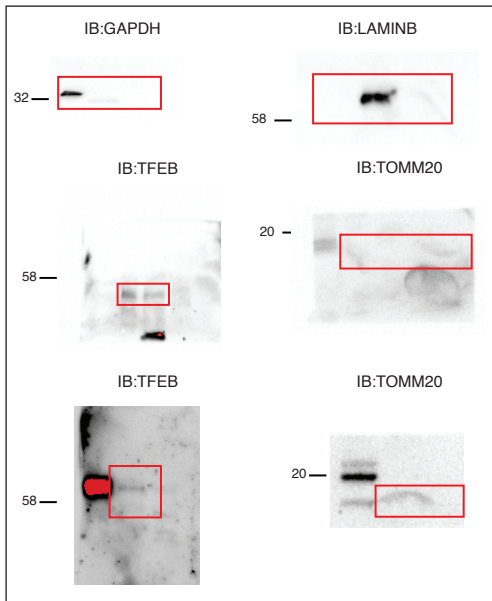

Supplement: Supplementary file 13 — Appendix Figures Source Data [file 44319_2024_58_MOESM13_ESM.zip › Appendix source data/Fig S1 Source data/Fig S1F/Fig S1F unprocessed blots.pdf]

**Fig. S1C**

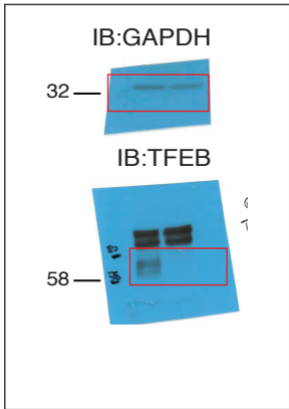

Supplement: Supplementary file 13 — Appendix Figures Source Data [file 44319_2024_58_MOESM13_ESM.zip › Appendix source data/Fig S1 Source data/Fig S1C/Fig S1C unprocessed blots.pdf]

**Fig. S1E**

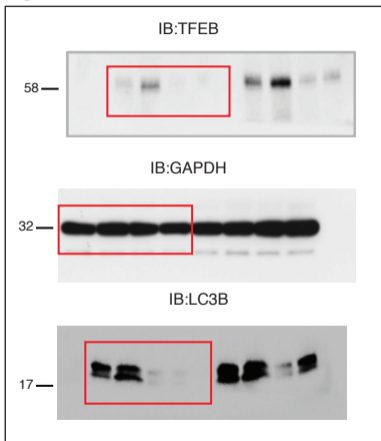

Supplement: Supplementary file 13 — Appendix Figures Source Data [file 44319_2024_58_MOESM13_ESM.zip › Appendix source data/Fig S1 Source data/Fig S1E/Fig S1E unprocessed blots.pdf]

**Fig. S4D**

IB:FLAG

75—

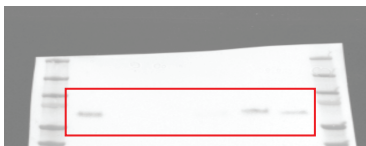

IB:GAPDH

32—

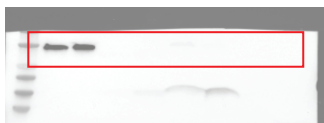

IB:LAMINB

50—

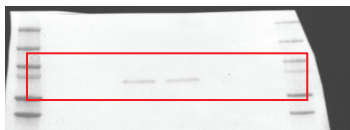

IB:TOMM20

11—

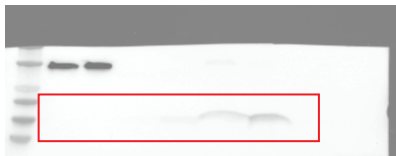

Supplement: Supplementary file 13 — Appendix Figures Source Data [file 44319_2024_58_MOESM13_ESM.zip › Appendix source data/Fig S4 Source data/Fig S4D/Fig S4D unprocessed blots.pdf]

**Fig. S4C**

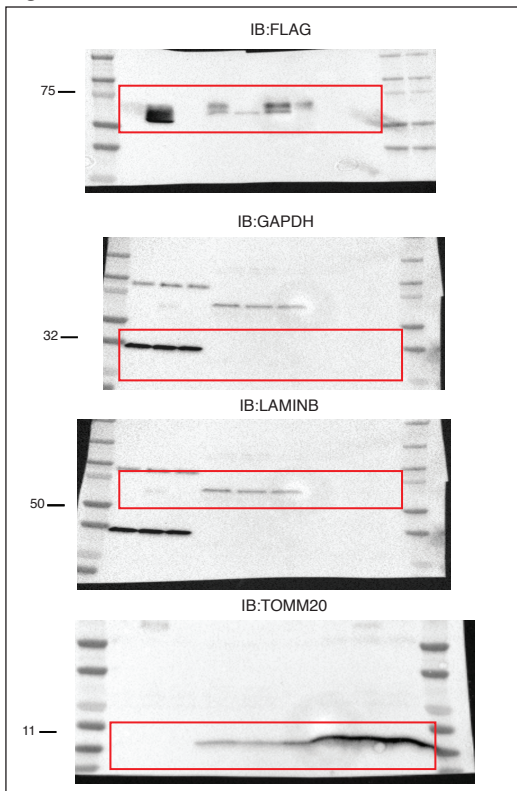

Supplement: Supplementary file 13 — Appendix Figures Source Data [file 44319_2024_58_MOESM13_ESM.zip › Appendix source data/Fig S4 Source data/Fig S4C/Fig S4C Unprocessed blots.pdf]

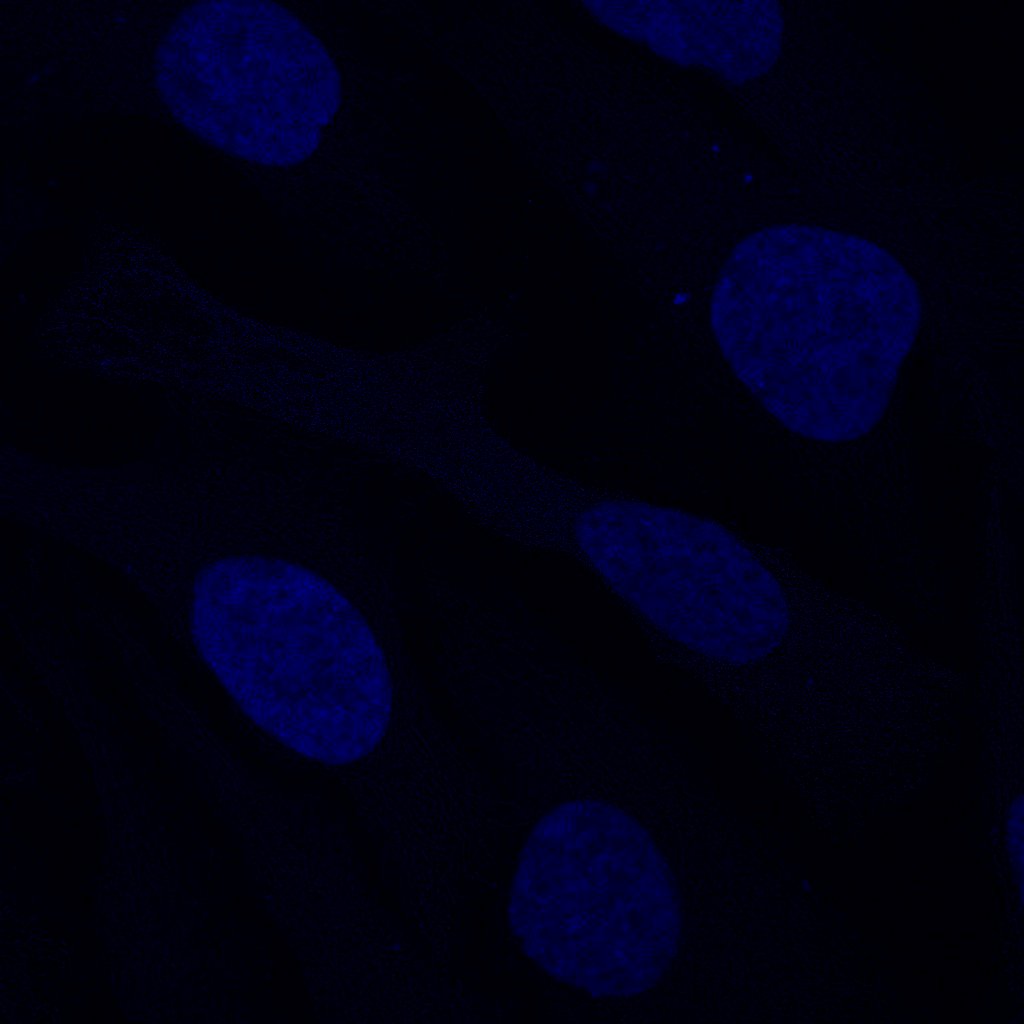

Supplement: Supplementary file 13 — Appendix Figures Source Data [file 44319_2024_58_MOESM13_ESM.zip › Appendix source data/Fig S4 Source data/Fig S4E/Fig S4E MTS-TFEB_DAPI.jpg]

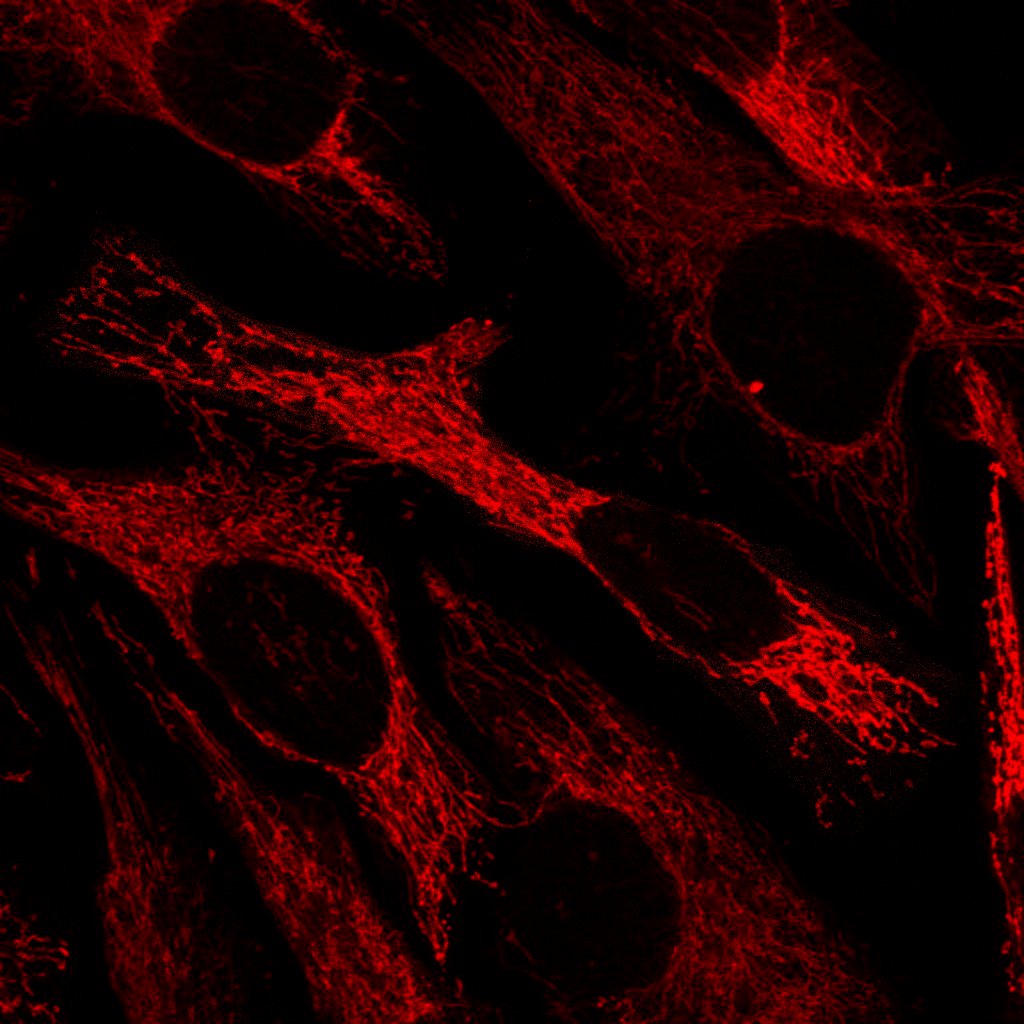

Supplement: Supplementary file 13 — Appendix Figures Source Data [file 44319_2024_58_MOESM13_ESM.zip › Appendix source data/Fig S4 Source data/Fig S4E/Fig S4E MTS-TFEB_Mitotracker.jpg (red).jpg]

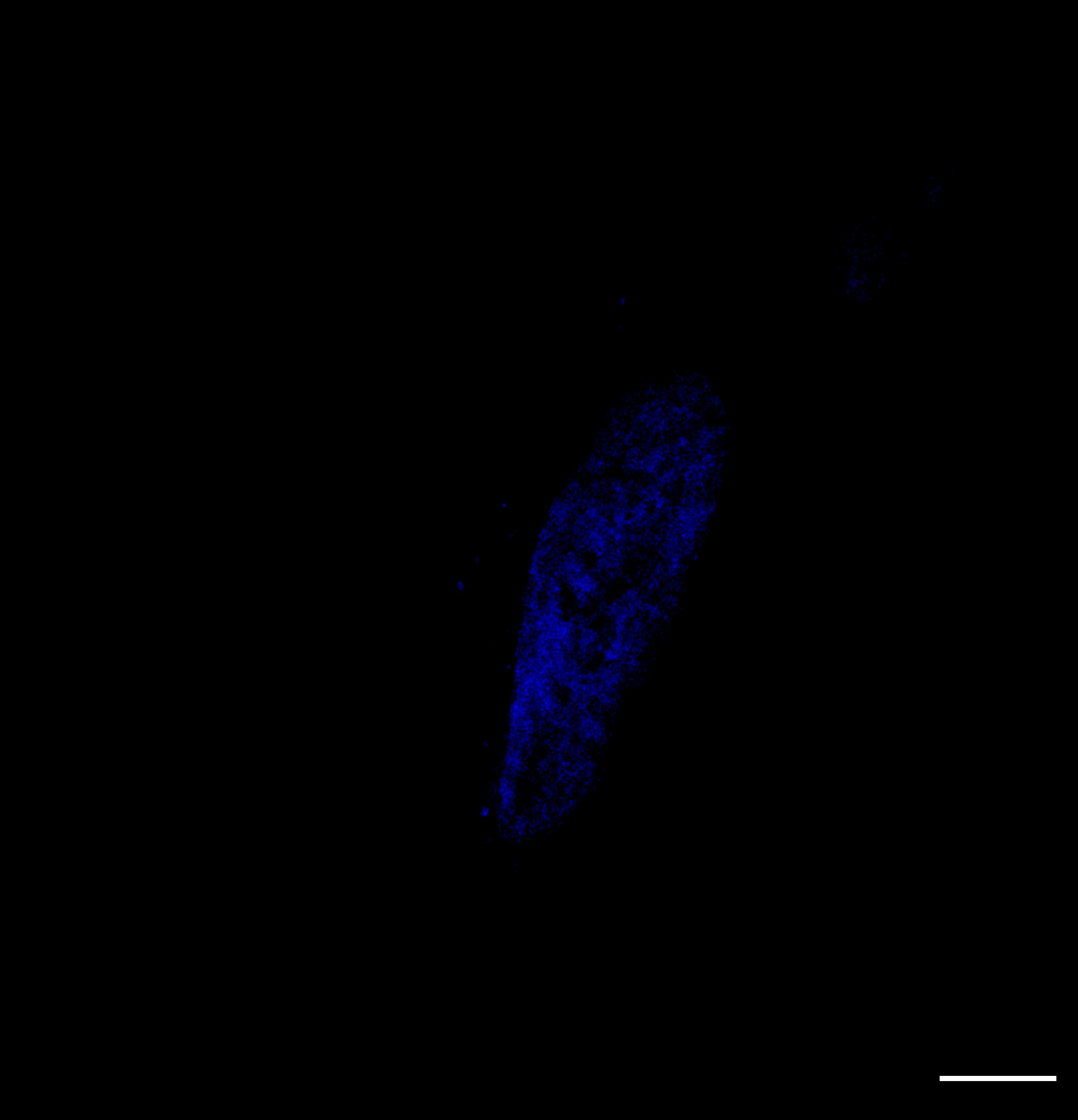

Supplement: Supplementary file 13 — Appendix Figures Source Data [file 44319_2024_58_MOESM13_ESM.zip › Appendix source data/Fig S4 Source data/Fig S4E/Fig S4E S142A:S211A-TFEB_DAPI.jpg]

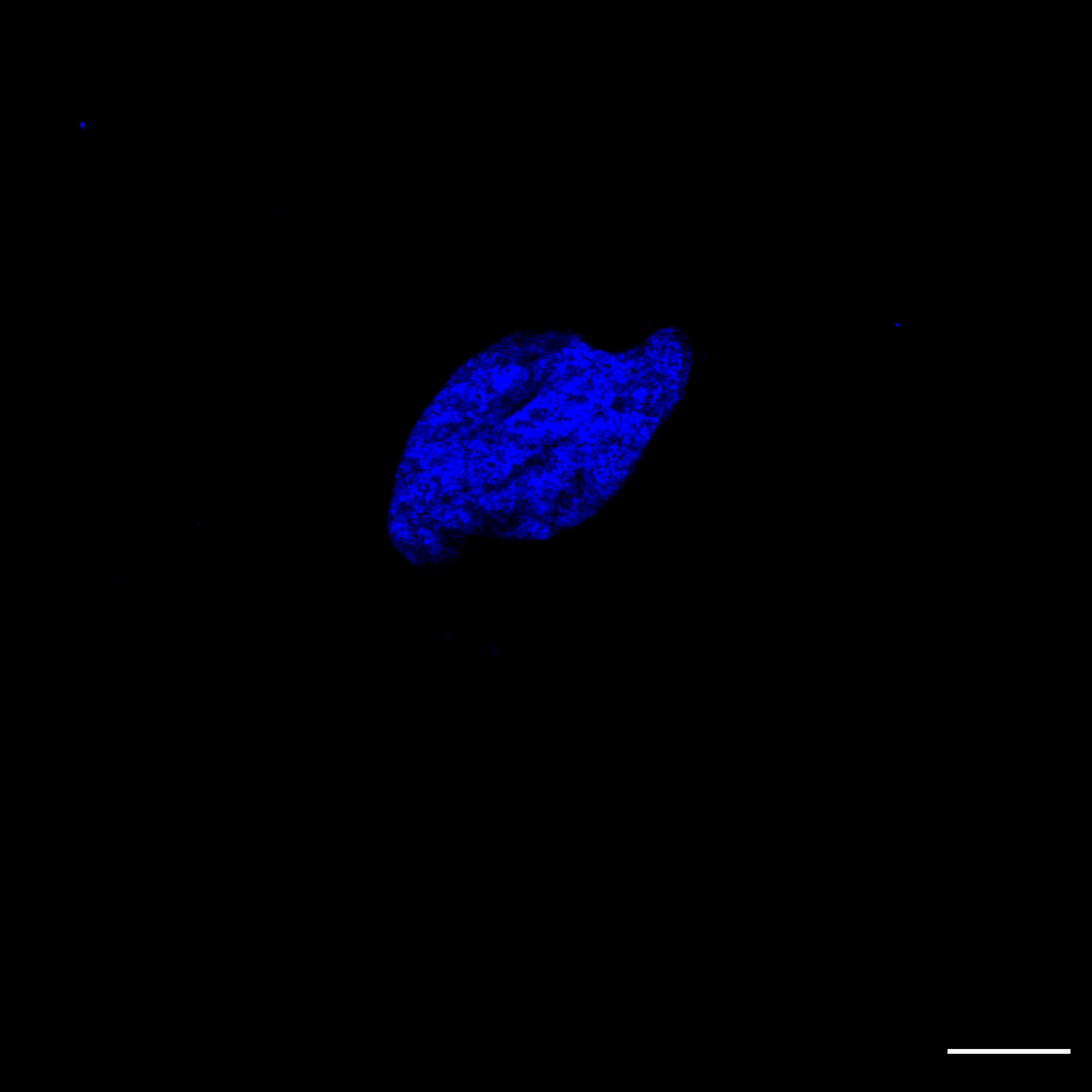

Supplement: Supplementary file 13 — Appendix Figures Source Data [file 44319_2024_58_MOESM13_ESM.zip › Appendix source data/Fig S4 Source data/Fig S4E/Fig S4E NLS-TFEB_DAPI.jpg]
