# Supplementary figures and images for: Mitochondrial translocation of TFEB regulates complex I and inflammation (part 2 of 2)
Source: EMBO Rep. 2024 Jan 23;25(2):704–24. doi: 10.1038/s44319-024-00058-0 (PMC10897448; doi:10.1038/s44319-024-00058-0)

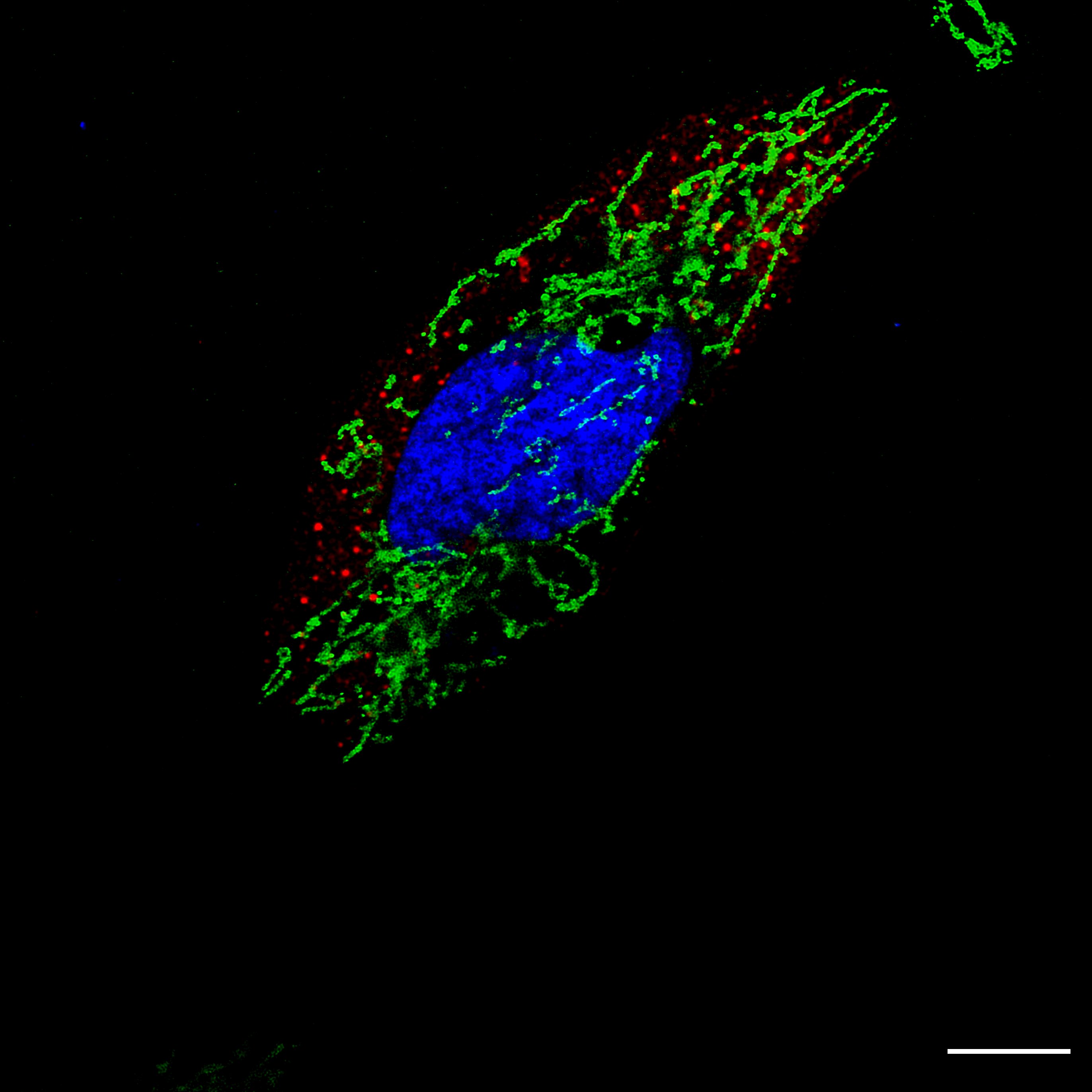

Supplement: Supplementary file 13 — Appendix Figures Source Data [file 44319_2024_58_MOESM13_ESM.zip › Appendix source data/Fig S4 Source data/Fig S4E/Fig S4E NLS-TFEB_Merged.jpg]

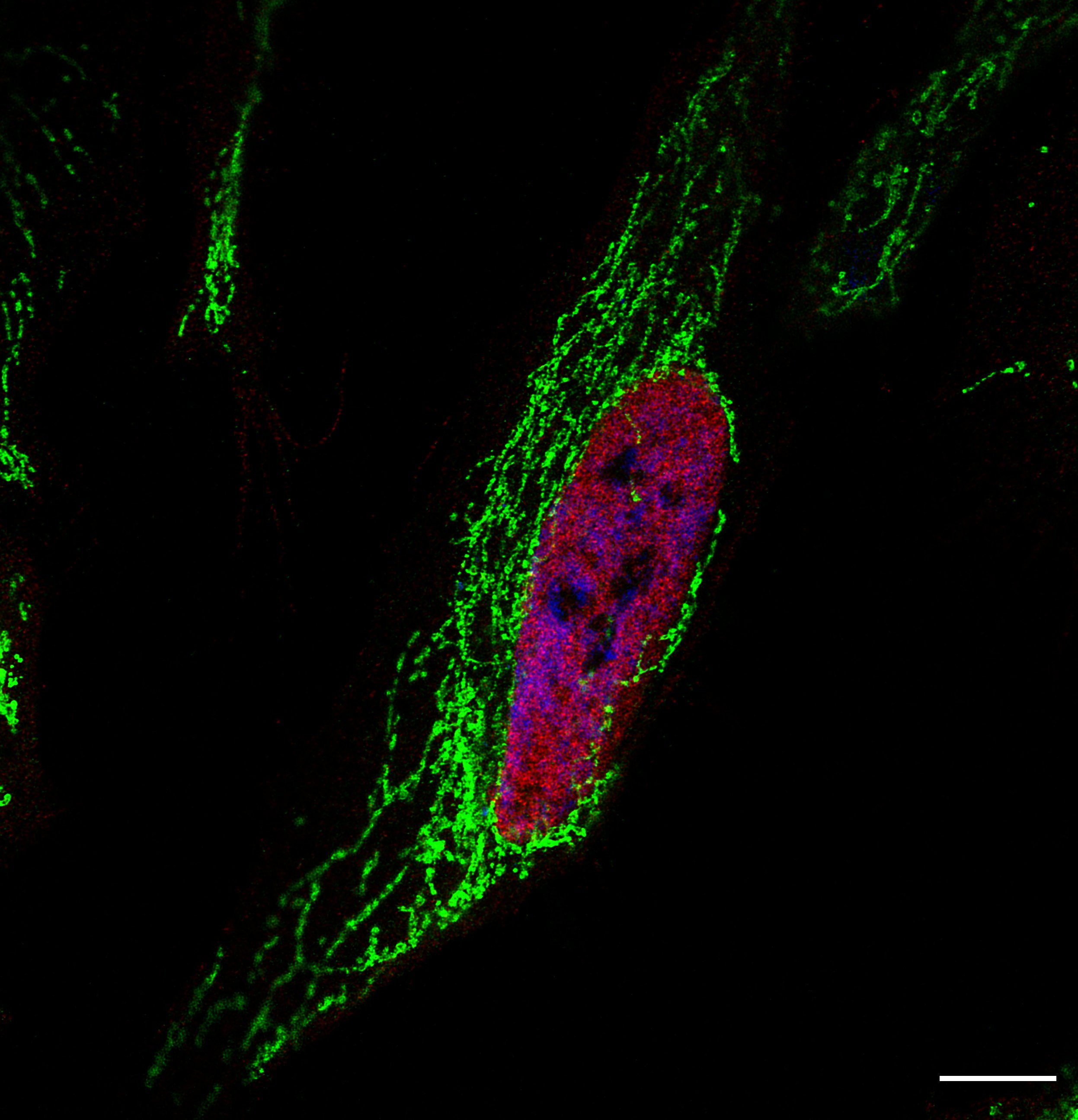

Supplement: Supplementary file 13 — Appendix Figures Source Data [file 44319_2024_58_MOESM13_ESM.zip › Appendix source data/Fig S4 Source data/Fig S4E/Fig S4E S142A:S211A-TFEB_Merged.jpg]

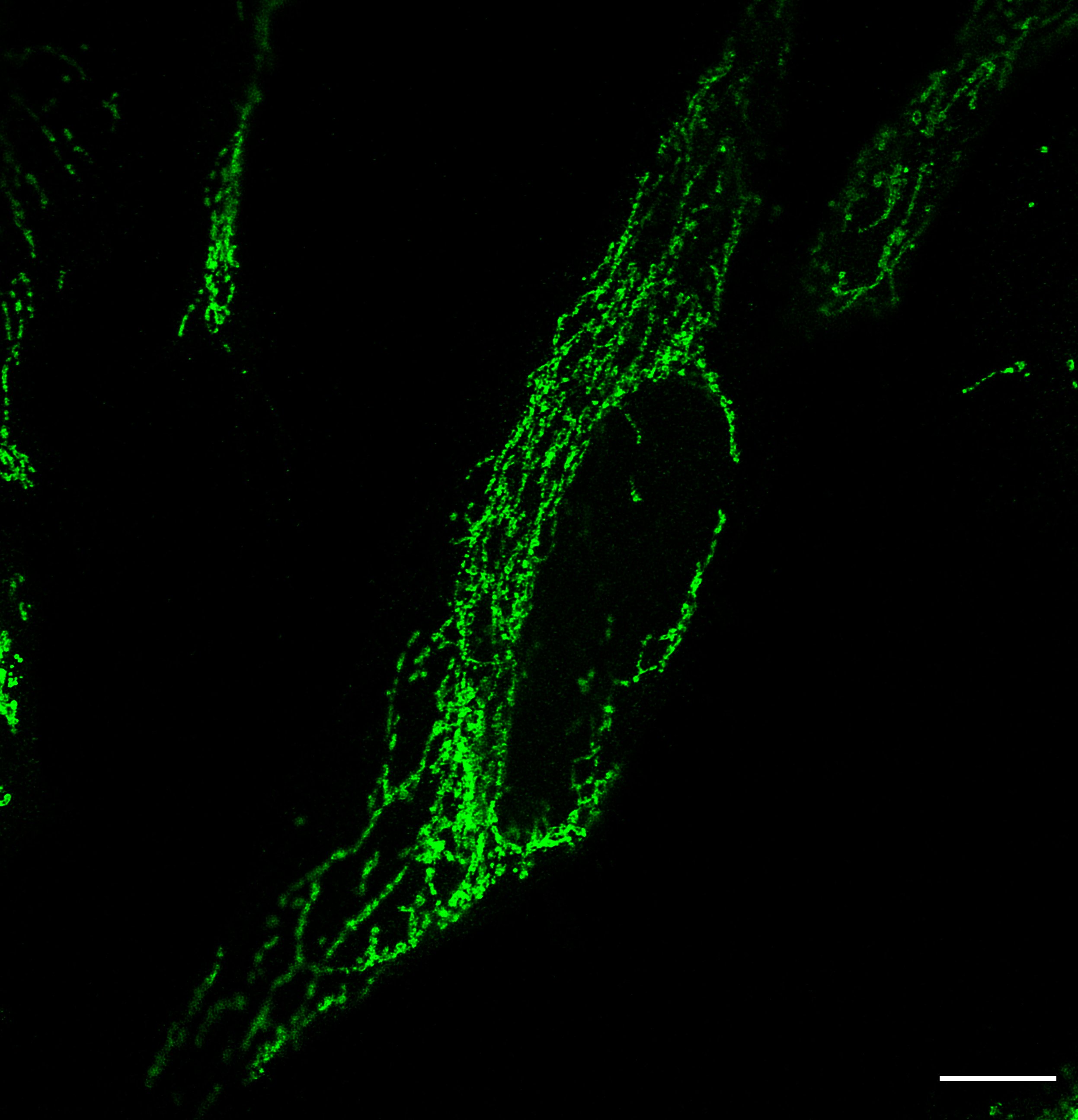

Supplement: Supplementary file 13 — Appendix Figures Source Data [file 44319_2024_58_MOESM13_ESM.zip › Appendix source data/Fig S4 Source data/Fig S4E/Fig S4E S142A:S211A-TFEB_Mitotracker.jpg]

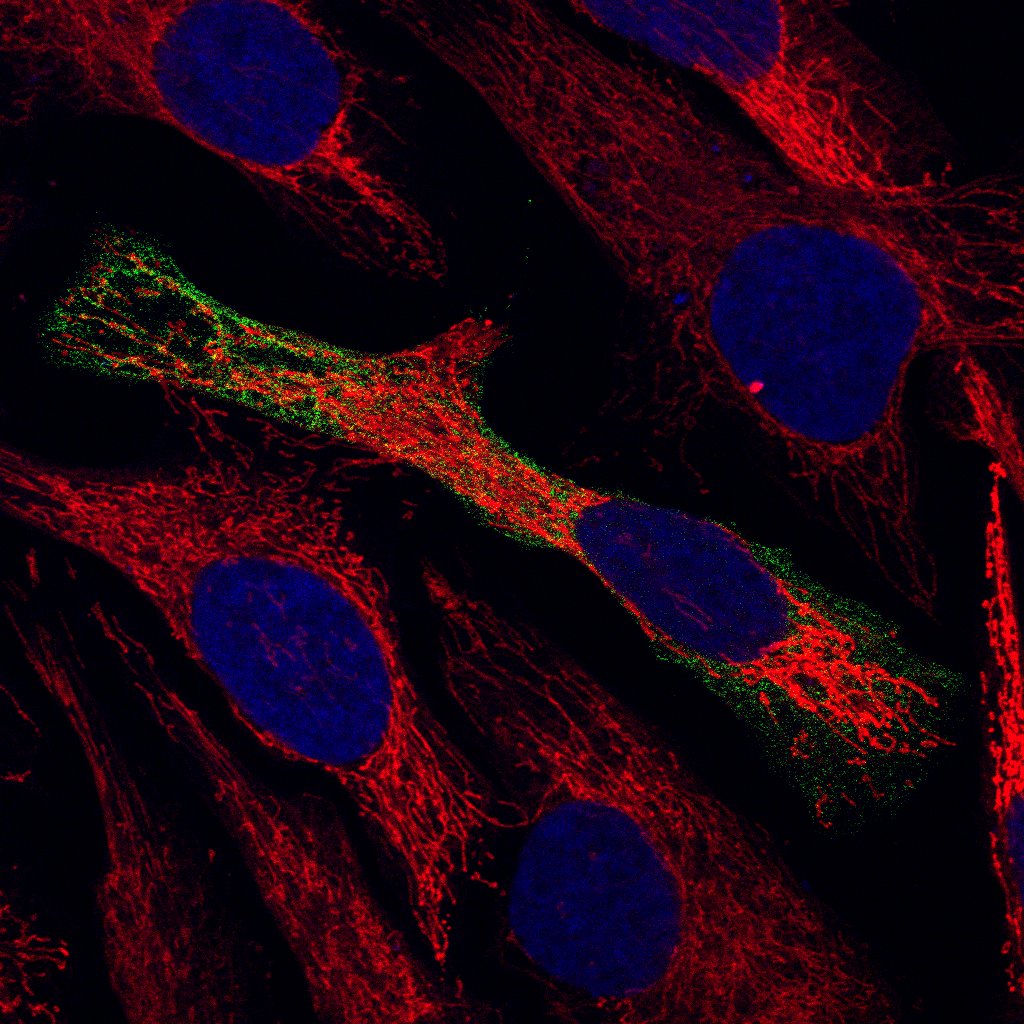

Supplement: Supplementary file 13 — Appendix Figures Source Data [file 44319_2024_58_MOESM13_ESM.zip › Appendix source data/Fig S4 Source data/Fig S4E/Fig S4E MTS-TFEB_Merged.jpg]

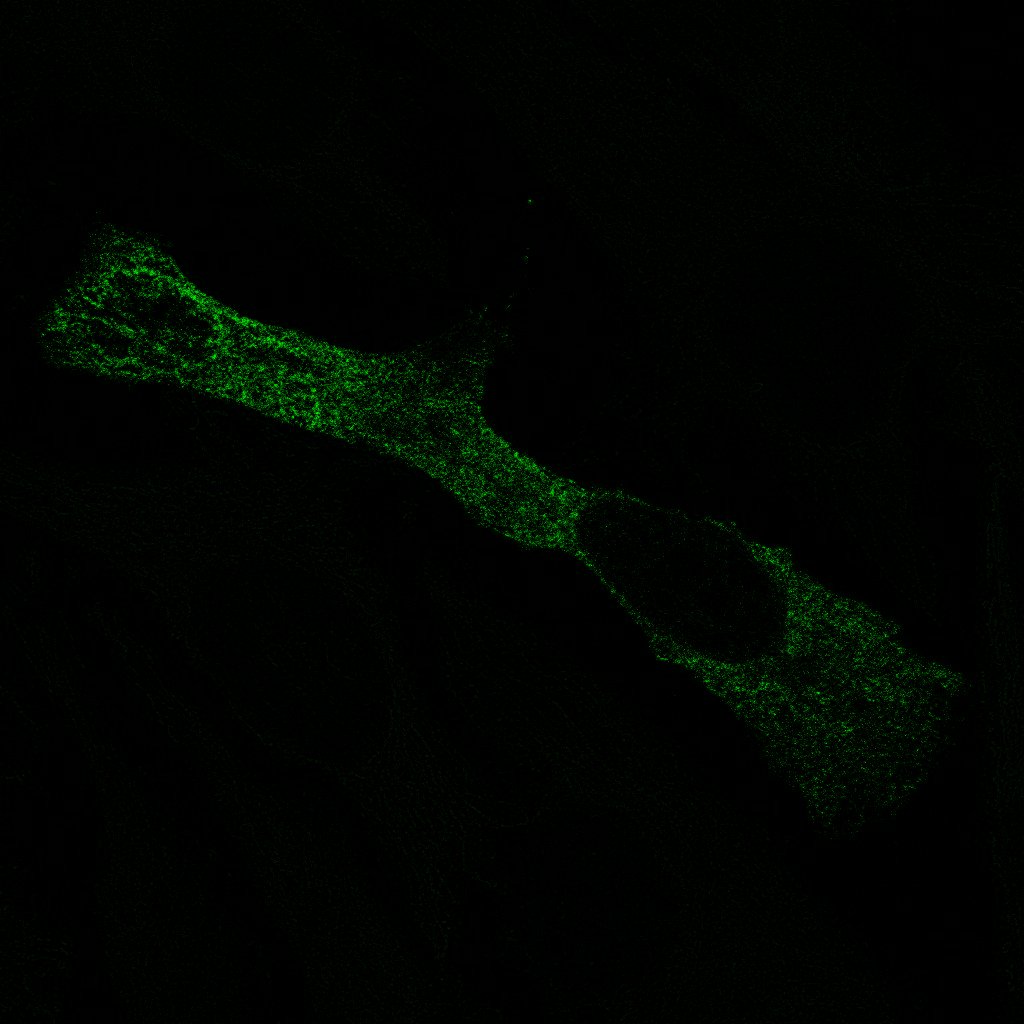

Supplement: Supplementary file 13 — Appendix Figures Source Data [file 44319_2024_58_MOESM13_ESM.zip › Appendix source data/Fig S4 Source data/Fig S4E/Fig S4E MTS-TFEB_FLAG.jpg]

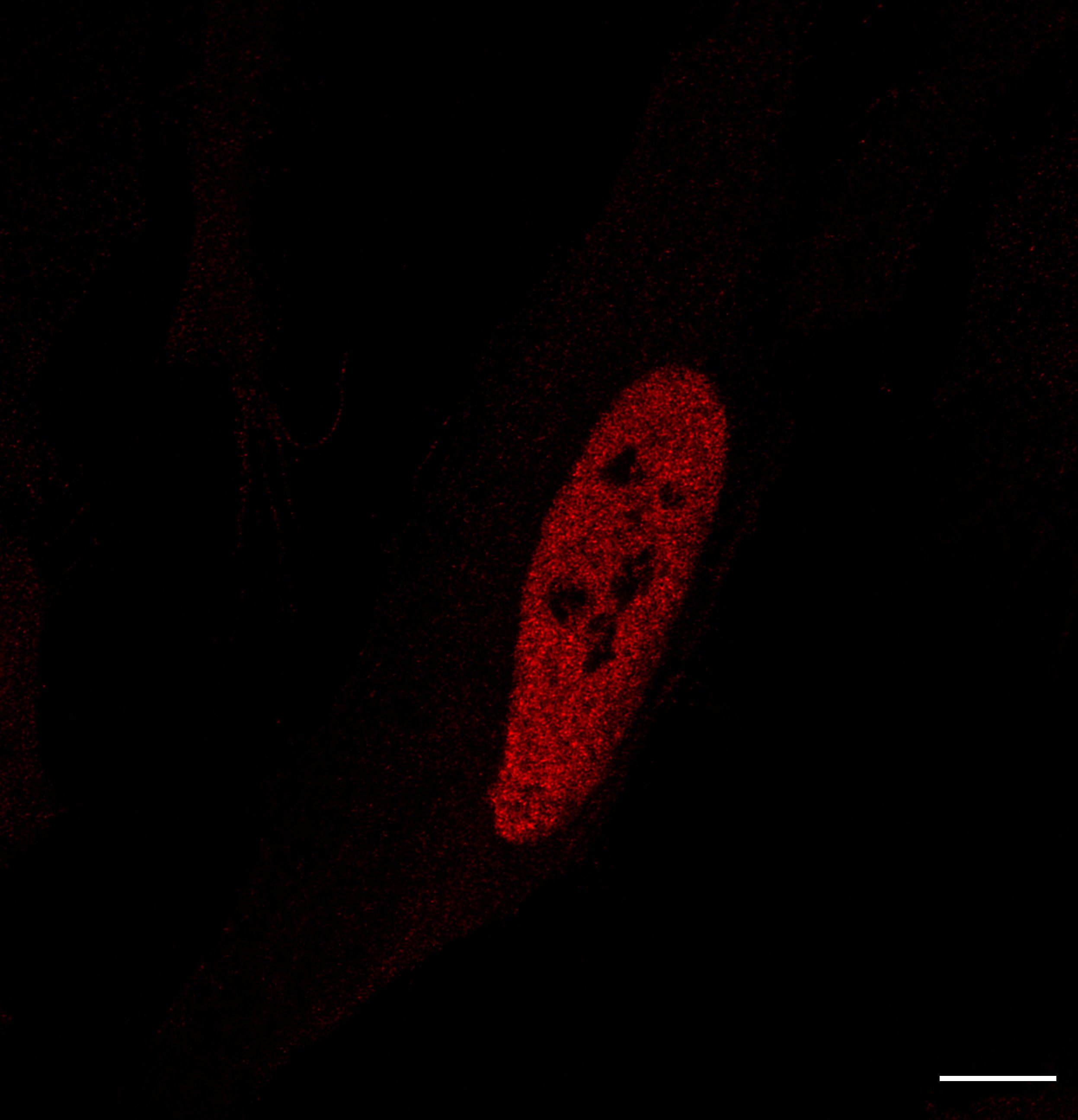

Supplement: Supplementary file 13 — Appendix Figures Source Data [file 44319_2024_58_MOESM13_ESM.zip › Appendix source data/Fig S4 Source data/Fig S4E/Fig S4E S142A:S211A-TFEB_FLAG.jpg]

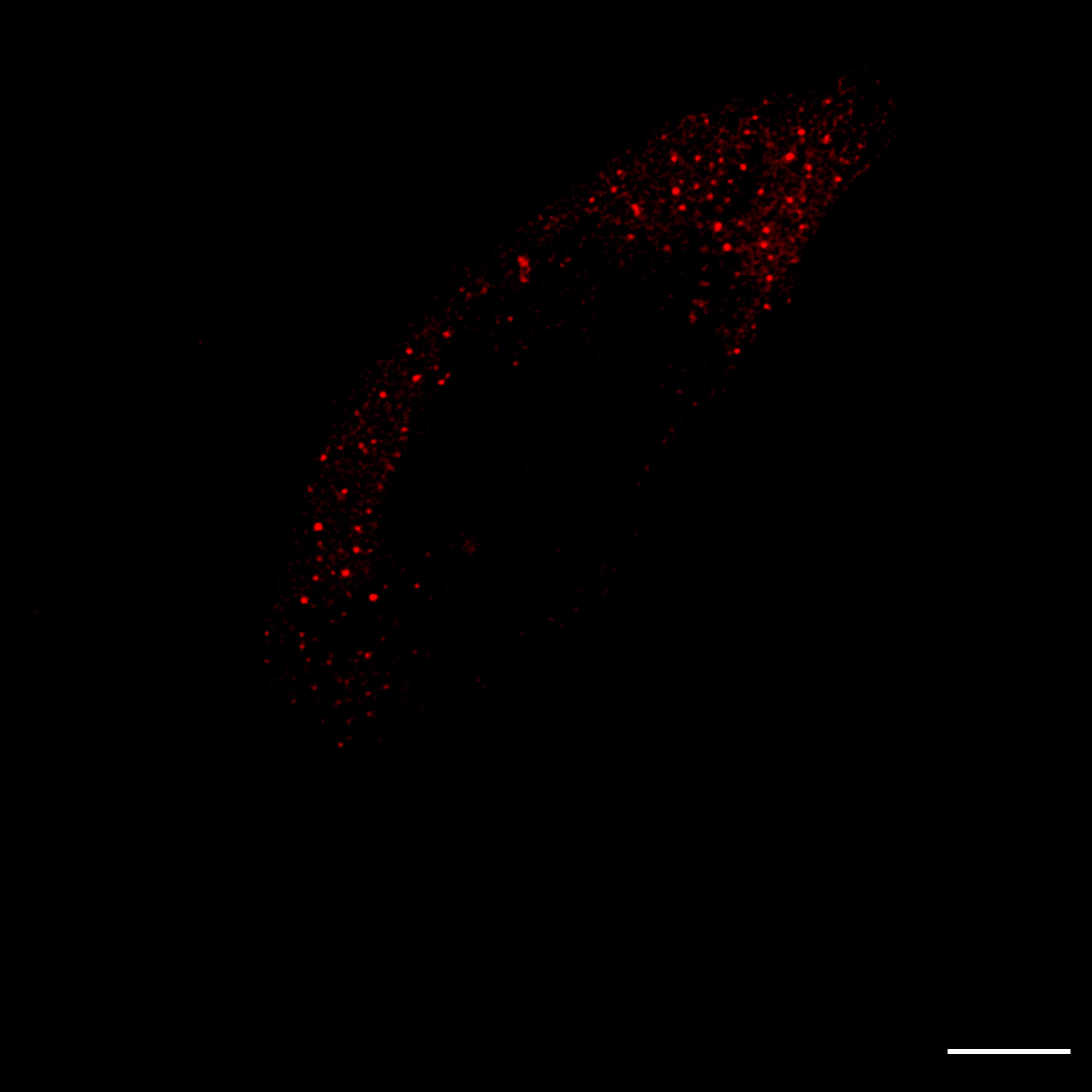

Supplement: Supplementary file 13 — Appendix Figures Source Data [file 44319_2024_58_MOESM13_ESM.zip › Appendix source data/Fig S4 Source data/Fig S4E/Fig S4E NLS-TFEB_FLAG.jpg]

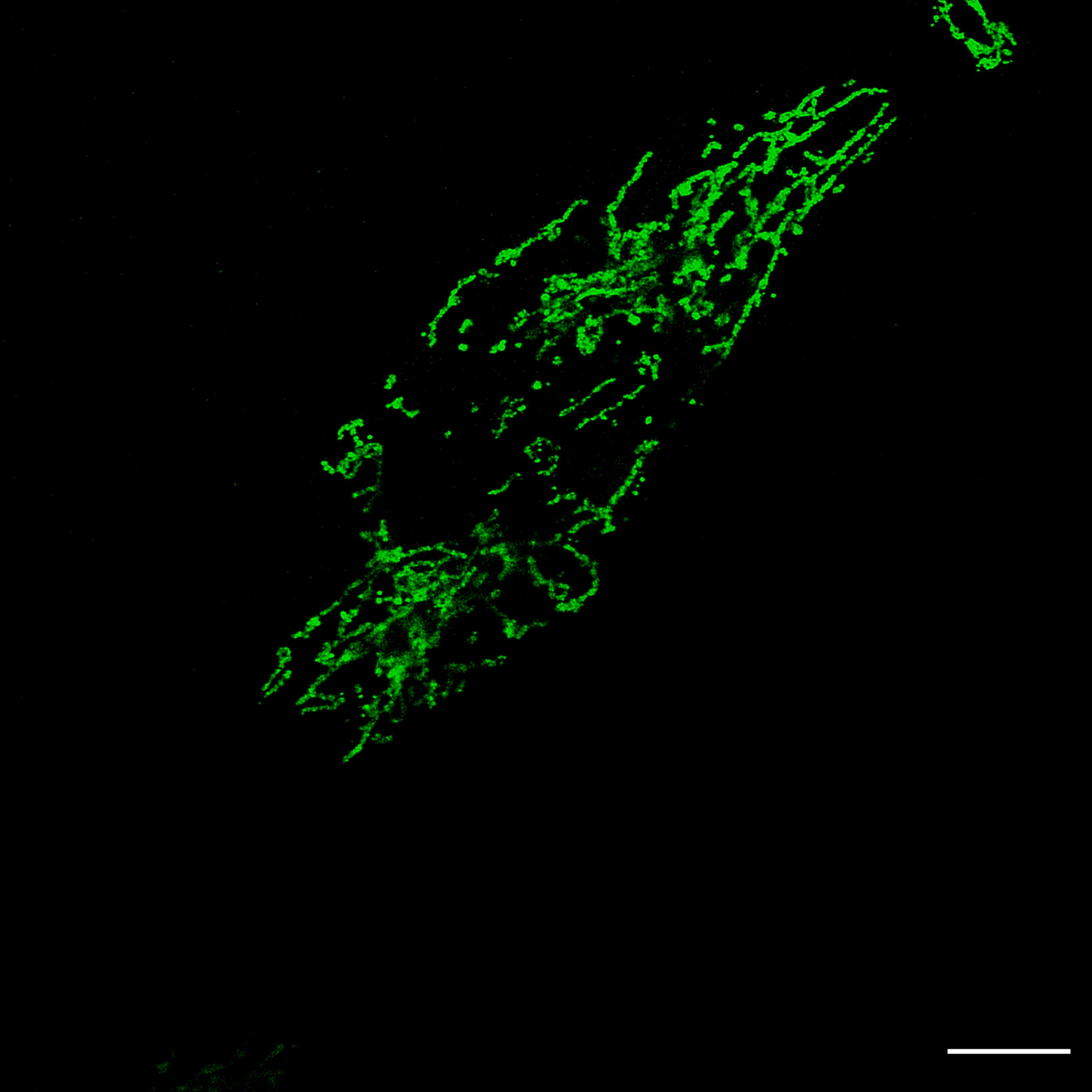

Supplement: Supplementary file 13 — Appendix Figures Source Data [file 44319_2024_58_MOESM13_ESM.zip › Appendix source data/Fig S4 Source data/Fig S4E/Fig S4E NLS-TFEB_Mitotracker.jpg]
